# Characterization of Exosome-like Nanoparticles from Saffron Tepals and Their Immunostimulatory Activity

**DOI:** 10.3390/biology14020215

**Published:** 2025-02-18

**Authors:** Cristian Martínez Fajardo, Alberto J. López-Jiménez, Susana López-López, Lucía Morote, Elena Moreno-Giménez, Gianfranco Diretto, María José M. Díaz-Guerra, Ángela Rubio-Moraga, Oussama Ahrazem, Lourdes Gómez-Gómez

**Affiliations:** 1 Instituto Botánico, Departamento de Ciencia y Tecnología Agroforestal y Genética, Universidad de Castilla-La Mancha, Campus Universitario s/n, 02071 Albacete, Spain; cristian.martinez@uclm.es (C.M.F.); albertojose.lopez@uclm.es (A.J.L.-J.); lucia.morote@uclm.es (L.M.); elena.morenogimenez@uclm.es (E.M.-G.); angela.rubio@uclm.es (Á.R.-M.); oussama.arhazem@uclm.es (O.A.); 2 Escuela Técnica Superior de Ingenieros Agrónomos, Montes y Biotecnología, Departamento de Ciencia y Tecnología Agroforestal y Genética, Universidad de Castilla-La Mancha, Campus Universitario s/n, 02071 Albacete, Spain; 3 Unidad de Investigación, Complejo Hospitalario Universitario de Albacete, C/Laurel, s/n, 02008 Albacete, Spain; susana.lopezlopez@uclm.es; 4 Facultad de Medicina, Departamento de Química Inorgánica, Orgánica y Bioquímica, Universidad de Castilla-La Mancha, Campus Universitario s/n, 02071 Albacete, Spain; mariajose.martinez@uclm.es; 5 Italian National Agency for New Technologies, Energy, and Sustainable Development, Casaccia Research Centre, 00123 Rome, Italy; gianfranco.diretto@enea.it; 6 Facultad de Farmacia, Departamento de Ciencia y Tecnología Agroforestal y Genética, Universidad de Castilla-La Mancha, Campus Universitario s/n, 02071 Albacete, Spain

**Keywords:** exosomes, inflammation, metabolites, miRNA, saffron, tepals

## Abstract

Plant exosomes exhibit high stability and easy absorption, and have emerged as promising bioactive tools due to their potential health benefits and biomedical applications. Saffron tepals contain abundant metabolites with potential therapeutic properties and were used for exosome extraction by ultracentrifugation and gradient purification. The exosomes showed an average particle size of 151.5 ± 79.6 nm and exhibited a spherical morphology. Five well-conserved miRNAs—miR157, miR166, miR168, miR396, and miR398—were identified in the exosomes, which are involved in the coordination of growth and physiological plant responses with endogenous and environmental abiotic and biotic signals, and their potential targets in mammals are upregulated in specific cancer types and associated with inflammation. Proteome analysis revealed an enrichment of proteasome proteins, ribosomal proteins, and proteins involved in the cytoskeleton, transport across the membrane (ABC transporters), and vesicle trafficking (RAB GTPases, TM9SF and Coatomer subunits). Metabolite analyses showed mainly anthocyanins. The exosomes have selective stimulatory activity on macrophages, increasing the expression of surface molecules (*CD80* and *CD86*), and cytokines (*IL-1β*, *IL-6*, and *TNF-α*), but not the levels of *IL-10*. Overall, these results indicated that saffron flowers are an effective and abundant source of exosomes as new nanomedicines for human health.

## Simple Summary

Saffron tepals constitute a rich source of bioactive metabolites, but are considered waste material after saffron processing. Herein, we obtained exosomes from this material and explored their potential for biomedical applications. Exosomes were isolated through ultracentrifugation following gradient purification, revealing an average particle size of 151.5 ± 79.6 nm with an exosome-like morphology. Detailed analysis identified five well-conserved plant miRNAs—miR157, miR166, miR168, miR396, and miR398—which are involved in regulating plant growth and responses to both biotic and abiotic stress and have potential mammalian targets that are upregulated in certain cancer types and are associated with inflammation. Proteomic analysis of the exosomes revealed an enrichment in proteasome components, ribosomal proteins, and proteins involved in cellular processes such as cytoskeletal organization, membrane transport, and vesicle trafficking. Furthermore, metabolite profiling identified anthocyanins as the predominant metabolites in the exosomes, which are known for their antioxidant properties. Functional assays demonstrated that saffron-derived exosomes selectively activate macrophages, increasing the expression of surface markers and pro-inflammatory cytokines. These findings suggest that saffron tepals are a promising and abundant source of exosomes with potential applications in nanomedicine and immune modulation for therapeutic purposes.

## 1. Introduction

Food and agricultural industries produce millions of tons of by-products and wastes each year, imposing a substantial financial burden on processors and contributing to significant environmental challenges. These wastes and by-products are generated across the entire supply chain. Specifically, vegetable and fruit wastes and/or by-products generated by these agro-industries typically consist of seeds, peels, and pomaces [1]. Irrespective of their origin, these underexploited residual biomasses are rich in various bioactive-functional ingredients, including dietary fiber, polyunsaturated fatty acids, carotenoids, and phenolic compounds [2]. This approach unlocks new possibilities by using underutilized local raw materials and by-products rich in anthocyanins and flavonoids. These can add value and offer innovative solutions for food companies and producers. As a result, food and agricultural waste can be transformed into bioactive compounds for functional foods, nutraceuticals, and cosmetics.

Exosomes, a subtype of extracellular cell-released vesicles smaller than 200 nm in diameter, are found in animals and plants, playing a crucial role in cell-to-cell communication and the transport of bioactive molecules and have emerged as versatile bioactive materials [3]. Their applications extend across drug delivery, disease treatment, and cell communication [4].

Bioactive compounds often face challenges in complete absorption and utilization by the body [5]. The solubility issues arise because many bioactive compounds are insoluble in water and susceptible to stress conditions, leading to a reduction in their stability and bioactivity. For instance, during food-waste processing, storage, and transportation, exposition to light, heat, and oxygen can result in the degradation of certain bioactive compounds [6]. In contrast, edible exosomes offer a more straightforward utility, the protective phospholipid bilayer shields them from the harsh conditions of the digestive tract, including the stomach’s acidic environment, protecting the bioactive substances and ensuring their stability [7]. Once absorbed in the intestine, edible exosomes can modulate local cellular functions and travel through the bloodstream to distant organs, where they exert effects on host tissues and contribute to the body’s overall systemic condition [8]. Therefore, utilizing exosomes as a functional component from food waste is a promising strategy for maximizing health benefits [9].

*Crocus sativus* is an autumnal geophyte belonging to the Iridaceae family, whose desiccated stigmas constitute the spice saffron [10]. The color, flavor, and the therapeutic properties of the spice are due to the presence of the apocarotenoids picrocrocin, safranal, and crocins [11]. The flowers consist of three yellow stamens and six purple tepals, and both tissues represent the most abundant bio-residue generated after flower processing ~98% of total flower mass [12]. Several studies have identified the secondary metabolites present in tepals and stamens [13,14,15]. Tepals are mainly characterized by the accumulation of anthocyanins, responsible for the purple color of the flower and flavonoids [16]. Modern pharmacological research has uncovered the remarkable therapeutic potential of saffron tepals, demonstrating powerful antioxidant, antidepressant, and immunomodulatory effects. These benefits stem from their potent anti-inflammatory and antioxidant properties, positioning saffron tepals as a promising natural resource for innovative medical and wellness applications [17,18,19,20]. The saffron crop’s low profitability in terms of biomass raises interest in the valorization of the generated flower waste [21]. Current research in saffron-processing waste is focused on the production of extracts rich in antioxidant polyphenols [22,23] and as antimicrobial agents [24,25].

This study focuses on transforming the waste produced during saffron dissection into a valuable source of bioactive exosomes. It represents the first comprehensive isolation, characterization, and assessment of the biological activities of saffron flower exosomes. By uncovering their potential, this research paves the way for the better utilization of saffron flower waste, offering new opportunities for its valorization in various therapeutic and biotechnological applications.

## 2. Materials and Methods

### 2.1. Isolation and Purification of Exosomes

Saffron (*Crocus sativus* L.)-processing waste comprising tepals and stamens was gathered from a processing plant in Tarazona de la Mancha (Albacete, Spain) immediately after the manual dissection of flowers. Phosphate-buffered saline (PBS) solution was added to the saffron tepals (1:2 g/mL) and chopped at high speed for 5 min with a blender, followed by filtration through four layers of cheesecloth. The filtrate was subjected to centrifugation at 2000 g for 20 min, followed by 5000 g for 40 min, and 10,000 g for 60 min to eliminate large plant tissues and cell debris. Exosomes were then isolated through ultracentrifugation at 180,000× *g* for 90 min and re-suspended in PBS (1×). For further purification, the suspension was placed on a discontinuous sucrose gradient (15%, 30%, 45%, and 60%, *w*/*v*) and ultracentrifuged again at 180,000× *g* for 90 min. Exosomes from the 45–60% layer were collected and washed three times. The exosome count was determined by quantifying their protein content using a BCA protein assay kit (Thermo-Fisher Scientific, Waltham, MA, USA).

### 2.2. Exosomes Characterization by Transmission Electron Microscopy (TEM) and Nanoparticle Tracking Analysis (NTA)

The morphology of the isolated exosomes was analyzed by transmission electron microscope (TEM) using a standardized procedure and observed using a FEI Tecnai G2 Spirit Biotwin 120 kw TEM at the CIPF (Centro de Investigación Principe Felipe, Valencia, Spain) electron microscopy facility.

The exosomes obtained were used to analyze the size distribution and concentration with a NanoSight LM-10 (Malvern, UK), version NTA 3.4 Build 3.4.4. Samples were diluted 1:100 in PBS (1×) and analyzed, with measurements recorded in triplicate over 60 s video intervals with Detect Threshold 3.

### 2.3. Small-RNA Sequencing and miRNA Analyses of Exosomes

RNA extraction was carried out by using Total Exosome RNA & Protein Isolation Kit (Thermo-Fisher Scientific). Sequencing libraries were prepared using the NEBNext Multiplex Small RNA Library Prep Set for Illumina (NEB) and sequenced on an Illumina Hiseq. 2500/2000 platform (Macrogen). Library quality was validated on an Agilent Bioanalyzer 2100 system. The RAW reads were processed, and Illumina adaptors removed, with “Trim Galore!” with the “remove poly-A” option (https://www.bioinformatics.babraham.ac.uk/projects/trim_galore/, accessed on 13 February 2025). The clean reads were mapped against the *C. sativus* genome (Chinese Academy of Sciences, accession number CRA007742) using MiRDeep2 Mapper, and miRdeep2 was used to identify miRNAs [26]. The annotation of miRNAs was performed by local alignment using nhmmer against the miRBase database (http://mirbase.org/), as well as a comparison with previous characterizations of miRNAs in saffron. Furthermore, the expression levels were quantified. Finally, potential targets of the identified miRNAs were searched with TargetFinder [27] against the CDS compilation of the *C. sativus* genome, and the hits annotated using BLASTX (https://blast.ncbi.nlm.nih.gov/Blast.cgi?LINK_LOC=blasthome&PAGE_TYPE=BlastSearch&PROGRAM=blastx, accessed on 13 February 2025).

### 2.4. Proteomic Analyses

#### 2.4.1. SDS-PAGE Analyses and In-Gel Digestion

SDS-PAGE (10% polyacrylamide) was run until the entire sample had migrated into the resolving gel, approximately 1 cm. The gel was then stained using the Colloidal Blue Staining Kit (Invitrogen). The entire proteome was excised, cut into small pieces, and subjected to in-gel digestion with trypsin (1). The gel was destained using 50 mM ammonium bicarbonate (ABC) (Sigma-Aldrich, Darmstadt, Germany) and 50% acetonitrile (ACN) (Fisher Chemical, Waltham, MA, USA). The sample was reduced with 10 mM dithiothreitol (Bio-Rad, Hercules, CA, USA) in 50 mM ABC and alkylated with 55 mM iodoacetamide (GE Healthcare Life Sciences, Pittsburgh, PA, USA) in 50 mM ABC. Gel pieces were then digested with porcine trypsin (Thermo-Fisher Scientific) at a final concentration of 12.5 ng/mL in 50 mM ABC overnight at 37 °C. Peptides were extracted using 100% ACN and 0.5% trifluoroacetic acid (Sigma-Aldrich), purified with a Zip Tip (Millipore, Sigma-Aldrich), and dried. The sample was reconstituted in 20 μL of 0.1% formic acid prior to analysis by nanoscale liquid chromatography–tandem mass spectrometry (nLC-MS/MS).

#### 2.4.2. LC-MS Analysis

Peptide separations were conducted using an Easy-nLC 1000 nano system (Thermo-Fisher Scientific), equipped with a pre-column, Acclaim PepMap 100 (Thermo-Fisher Scientific), attached to a RSLC PepMap C18 column (50 cm, 75 µm inner diameter, 2 µm; Thermo-Fisher Scientific). The flow rate was 300 µL/min, with solvent A consisting of 0.1% formic acid in water and solvent B containing 0.1% formic acid and 100% acetonitrile. Four microliters (1 µg) were injected. The gradient used for separation was as follows: 5–35% B for 100 min, 35–45% B for 20 min, 45–100% B for 5 min, and 100% B for 15 min. Mass spectrometry (MS) analysis was carried out using a Q-Exactive mass spectrometer (Thermo-Fisher Scientific). Ionization was achieved with a liquid junction voltage of 1900 V and a capillary temperature of 300 °C. The full-scan method covered a *m*/*z* range of 300–1800, with an Orbitrap resolution of 70,000 (at *m*/*z* 200), a target automatic gain control (AGC) value of 3 × 10^6^, and a maximum injection time of 100 ms. Following the survey scan, the 15 most intense precursor ions were selected for MS/MS fragmentation, using a normalized collision energy of 27 eV, and MS/MS scans were acquired with an *m*/*z* range starting at 200, AGC target of 23 × 10^5^, a resolution of 17,500 (at *m*/*z* 200), intensity threshold of 83 × 10^4^, an isolation window of 2.0 *m*/*z* units, and a maximum injection time of 100 ms. Charge-state screening was enabled to exclude unassigned, singly charged, or ions with seven or more protons. A dynamic exclusion time of 20 s was implemented to prevent previously selected ions from being analyzed again.

#### 2.4.3. MS Data Analysis

MS data were analyzed with Proteome Discoverer (version 1.4.1.14) (Thermo-Fischer Scientific) using standardized workflows. Mass spectra *.RAW files were searched against the UniProtKB/TrEMBL (unreviewed) and *Crocus sativus* (1773 sequences). Data analysis was performed using the Mascot search engine (version 2.6, Matrix Science). Precursor and fragment mass tolerances were set to 10 ppm and 0.02 Da, respectively, with allowances for up to 2 missed cleavages. The carbamidomethylation of cysteines was specified as a fixed modification, while the phosphorylation of serine, threonine, and tyrosine, methionine oxidation, and N-terminal acetylation were set as variable modifications. Identified peptides were filtered using the Percolator algorithm with a q-value threshold of 0.01. Data analysis for GO, KEGG pathways, and the Protein–Protein Interaction networks was carried out.

The exosomal proteins were analyzed by applying Gene Ontology (GO) and the KEGG pathway of G:profiler (https://biit.cs.ut.ee/gprofiler/gost, accessed on 13 February 2025). The relative annotations were categorized, and a high percentage value represents a greater functional enrichment. STRING 11.0 (https://string-db.org/) was applied for Protein–Protein Interaction (PPI) analysis. The PPI enrichment *p*-value was <1.0–16.

### 2.5. Metabolomics

For the analysis of carotenoids, anthocyanins, and flavonoids in the exosomes, the samples were extracted by the addition of chloroform and methanol (2:1) (with 50 mg/L α-toc as internal standard). Samples were shacked in Mixer Mill for 10 min at 20 Hz and then 50 mM Tris-HCl buffer (pH 7.5, containing 1 M NaCl) was added to them. Hereafter, the samples were mixed and centrifuged for 10 min at 12,000× *g* in a microcentrifuge. After that, the chloroform phases were collected and subjected to drying via a Speedvac. Once dried, the samples were re-suspended in 100 μL of HPLC-grade ethyl acetate and centrifuged to eliminate any residual impurities. The supernatant (50 μL) was then carefully transferred to HPLC sample analysis tubes. All analyses were performed in triplicate to ensure reliability. The HPLC-HRMS protocols used for the detection and quantification of flavonoids, anthocyanins, carotenoids, and other apolar metabolites were previously outlined in the literature [28]. Tentative identification was carried out according to their *m*/*z* accurate masses as included in the PubChem database for monoisotopic masses or by using the Mass Spectrometry Adduct Calculator from the Metabolomics Fiehn Lab for adduct ions. Analyses were performed in triplicate.

### 2.6. Cell-Culture Model and Treatment with Exosomes

Macrophage-line RAW 264.7 cells (ATCC No. TIB-71) were subcultured at 6–8 × 10^4^ cells/cm^2^ in DMEM medium (GIBCO BRL) with 10% FBS, 4 mM L-glutamine, and 1% penicillin–streptomycin, and incubated overnight in complete DMEM supplemented with 5% FBS, preceding activation with LPS (100 ng/mL for 8 h) and exosomes. To determine the immunostimulatory potential of saffron tepal exosomes, a treatment was carried out at a protein concentration of 25 µg/mL of exosomes. As a control of the inflammatory response, a treatment with LPS (100 ng/mL) was performed and the cells were incubated with the different treatments for 2, 4, 8, 12 and 24 h. Cell activation was evaluated for each experiment by determining the production of nitrites (Griess reaction) after pro-inflammatory stimuli.

### 2.7. RNA Isolation and Quantitative Real-Time Polymerase Chain Reaction (qPCR)

Real-time reverse transcriptional polymerase chain reaction was carried out as follows. Total RNA was obtained from control and treated cells with TRIzol reagent and then reverse-transcribed into cDNA with the RevertAid First Strand cDNA Synthesis Kit (Thermo-Fischer Scientific). Real-time PCRs were carried out using the Fast SYBR Green Protocol, with the StepOne real-time PCR system (Applied Biosystems, Waltham, MA, USA). For normalization, we used the housekeeping zero myelin gene (P0). The primers used are listed in Table S1.

### 2.8. Statistical Analysis

The results are presented as mean ± SD. Statistical analysis was performed using GraphPad Prism 8.0.2. Comparisons were made using either Student’s t-test, the Kruskal–Wallis test, or one-way analysis of variance (ANOVA), followed by Dunnett’s post hoc test, depending on the nature of the data.

## 3. Results

### 3.1. Characterization of Exosomes from Flower Waste of Saffron

**Figure 1:**
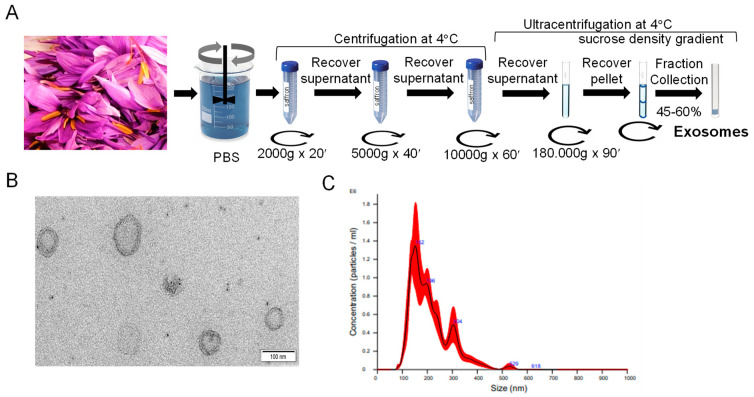
A scheme of the harvesting of exosomes from saffron-waste flowers. (**A**) A schematic representation of the protocol used to isolate and purify exosomes from saffron tepals. (**B**) Transmission electron microscopy image of nanovesicles isolated from saffron tepals. Scale bar is 100 nm. (**C**) Nanoparticle tracking analysis (NTA) of saffron-waste flowers.

Flower waste of saffron, consisting of floral remains resulting from the process of dissecting the flower to separate the stigmas, were used for exosome isolation by differential centrifugation and ultracentrifugation combined with gradient purification (Figure 1A). The exosomes were recovered in the 45–60% interphase. Isolated vesicles were characterized as exosome-like nanoparticles based on electron microscopic examination (Figure 1B). The exosomes displayed a typical cup-shaped membrane structures, resembling the well-established characteristics of exosomes as reported in previous studies [29]. Nanotracking analysis (NTA) showed particles with a major peak intensity of 152 nm (Figure 1C). The mean was 210.1 ± 5.8 nm, and mode 143.7 ± 6.5 nm. These parameters are valuable for defining the size range of exosomes and their frequency distribution. The concentration was 1.70 × 10^8^ ± 1.45 × 10^7^ particles/mL. The protein concentration obtained, 5 mg/mL, was analyzed by BCA.

### 3.2. Small-RNAs’ Results and Analyses of Target Genes in Saffron and Humans

According to our sequencing results, there were 32,603,060 raw reads, with a 49.5 % GC value. We first mapped the filtered reads to the saffron genome, and then the obtained reads were mapped onto known mature miRNAs in the plants miRbase. A total of 48 miRNAs were identified (Table 1), which groups in five families of miRNAs: miR157, miR166, miR168, miR398, and miR396. Among the five identified miRNA families, miR166 showed the major expression levels, with 60.97% of the total levels, followed by miR398 with 23.39%, miR396 with 10.87%, miR168 with 3.29%, and miR157 with 1.48%. A target-gene prediction analysis was conducted for the identified miRNAs using the saffron genome (Table S2). For miR157, miR166, and miR396, genes encoding for transcriptional factors were identified as potential targets including Squamosa promoter-binding-like protein, WRKY, zinc-finger-proteins, and Homeobox-leucine-zipper (Table S2). miRNAs regulate gene expression by binding to specific sites on target mRNAs, either inducing mRNA cleavage or inhibiting translation [30]. MiR166 ian evolutionarily conserved microRNA in plants whose primary targets include HD-ZIP III transcription factors, encompassing diverse gene members. MiR166 significantly influences fundamental plant processes, serving as a regulatory tool in various signaling pathways related to both abiotic and biotic stresses [31]. Notably, miR166 plays pivotal roles in plant defense mechanisms, exhibiting both upregulation and negative regulation under diverse abiotic stresses. In addition to miR166, miR398 also functions as a master regulator of plant responses to various environmental stresses and of plant growth and development [32]. miR396 serves as a critical hub, coordinating various growth and physiological responses with both endogenous and environmental signals [33].

**Figure 2:**
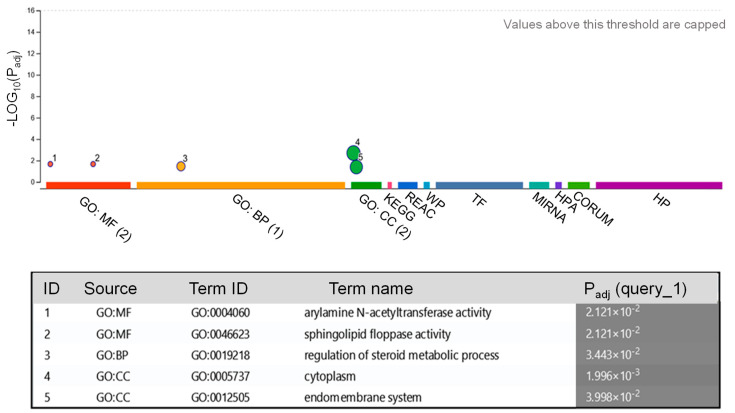
GO enrichment analyses of possible targets for the miRNAs from saffron-waste exosomes (Table S1). The ClusterProfiler analysis of significantly enriched GO terms within the molecular function (MF), biological processes (BP), and cell component (CC) categories.

Next, we predicted the target genes of the five identified miRNAs in the human genome (Table 2) and a total of 50 targets were found for miR396, while 5 were found for miR156, 2 for miR392, and only 1 for miR168 and miR166 (Figure 2). To achieve a deeper functional understanding of the miRNAs present in the tepal exosomes and the function of their possible targets, we proceeded with the Gene Ontology (GO) annotation (https://www.geneontology.org/) (Figure 2). This analysis revealed that the predicted target genes were linked to several functions (Figure 2). Specifically, within the cellular component category, they were primarily associated with the cell membrane and endomembrane system. In the molecular function category, the two identified targets were related to drug-metabolizing activity [34] and lipid transport. Further, the GO term biological process was associated with the regulation of steroid metabolic processes (Figure 2).

In addition, we assessed the implications of the identified targets in our study (Table 2) in different diseases/pathologies (Table S2). A total of 11 genes have been shown to be related with specific pathological conditions (Table S3), with implications in leukemia, myeloma, kidney, liver, glioma, prostate, thyroid, adrenocorticoid, and renal cancer.

### 3.3. Proteomic Analyses of Saffron Flower Waste Exosomes

**Figure 3:**
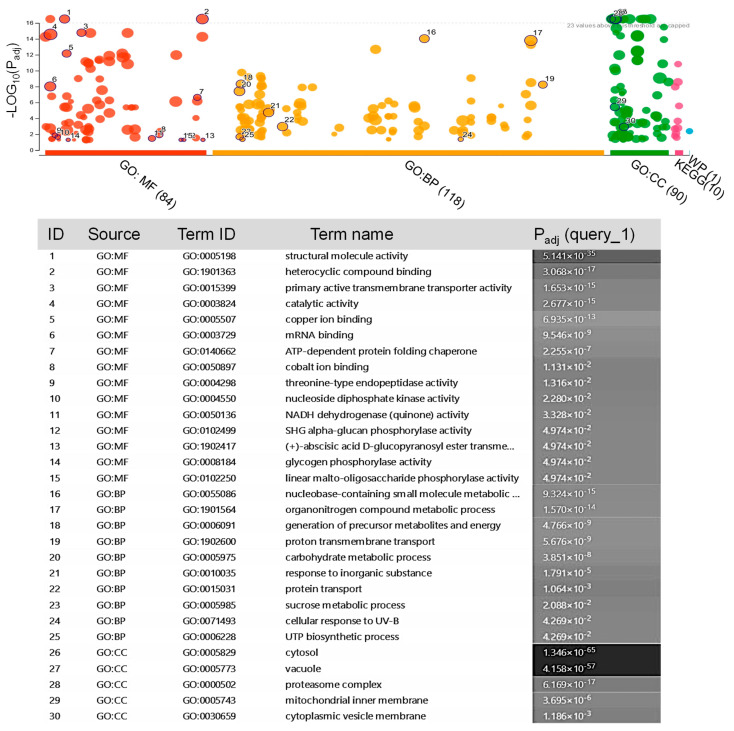
GO enrichment analysis of the proteins identified in the saffron-waste exosomes (Table S4). The ClusterProfiler analysis of significantly enriched GO terms within the molecular function (MF), biological processes (BP), and cell component (CC) categories.

In total, 27 proteins belonging to saffron were confidently identified with high accuracy (Table S4). However, the saffron proteome has not been extensively studied until the date. For this reason, many of the obtained peptides were not associated with any known protein. Therefore, the peptides were searched against UniProtKB/TrEMBL database and a total of 349 protein homologues to Arabidopsis were identified (Table S5). The potential role of these proteins in the exosomes was evaluated for GO functional classification based on their molecular function, biological processes, and cellular component. As shown in Figure 3, the most significant clusters of molecular function were related to processes of structural molecular activity, primary active transmembrane transporter, and catalytic activities. There was an enrichment of GO biological processes related to metabolism/catabolism/biosynthesis, and transport. In the cellular component, the terms that were enriched include the cytosol, vacuole, and proteasome complex (Figure 3).

The identified exosome proteins were classified into 10 KEGG pathways (Figure 3). The results indicated that the exosome proteins were linked to metabolic pathways, proteasomes, oxidative phosphorylation, ribosomes, and the regulation of actin cytoskeleton, as well as the biosynthesis of secondary metabolites, carbon metabolism, phagosome, glycolysis/gluconeogenesis, amino sugar and nucleotide sugar metabolism, and endocytosis.

### 3.4. Proteome Functional Analyses

To better understand the interaction of the exosome proteins, we constructed a PPI network through STRING (https://string-db.org/), which revealed a highly interconnected network. The interactions were divided in eight main clusters out of a total of fifteen (Figure S1 and Table S6), and >50% of the total proteins were present in just three clusters, containing ribosomal proteins, proteins involved in the proteosome machinery, and proteins involved in oxidative reactions (Tables S6 and S7). The obtained results are similar to the ones previously obtained from the proteome of exosomes of kiwi [35] and exosomes derived from the apoplastic fluid of tobacco [36]. An additional cluster contains chaperones and cytoskeletal proteins, which were closely related to proteins involved in membrane trafficking and exocyst complexes (Figure S1 and Tables S6 and S7).

### 3.5. Secondary Metabolites in Saffron Flower Waste Exosomes

The floral residues used for exosome purification showed the presence of flavonoids, anthocyanins, and carotenoids as major components (Figure S2). Therefore, we searched for these compounds in polar and non-polar extracts obtained from the isolated exosomes. HPLC-MS analyses revealed that non-polar extracts contained carotenoids like lutein, zeaxanthin, and α- and β-carotene (Table 3); plastoquinone and ubiquinone 10 were detected as well. The polar fraction revealed the presence of one flavonoid, kaempferol-3,7-di-*O*-glucoside, and only one anthocyanin class, delphinidin-3,5-di-*O*-glucoside (Table 3).

### 3.6. Exosomes Induction of Expression of Anti-Inflammatory Genes

**Figure 4:**
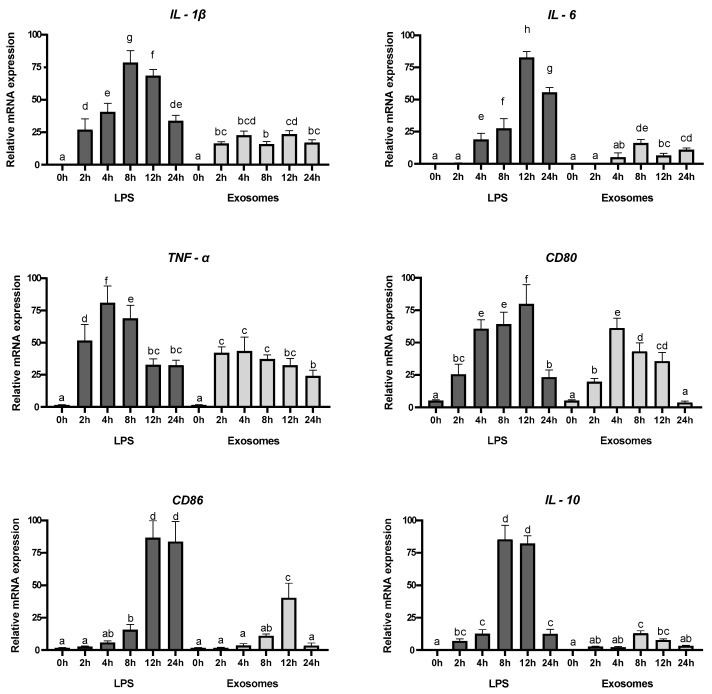
Relative expression levels of different pro-inflammatory genes in RAW 264.7 macrophages treated with exosomes or LPS as positive control. Error bars represent the average values ± SD (n = 9). Letters denote statistical significance between conditions in a one-way ANOVA (Tukey’s multiple-comparisons test, *p* ≤ 0.05).

Inflammatory diseases of the digestive system, like ulcerative colitis (UC) and Crohn’s disease (CD), are chronic and challenging to treat, often severely affecting quality of life. UC primarily affects the colon, causing persistent, superficial inflammation that extends from the mucosa to the submucosa. In contrast, CD causes irregular, deep inflammation (transmural) that has the potential to influence any part of the gastrointestinal tract. Macrophages are crucial in maintaining intestinal immune balance and contribute to the development of both UC and CD [37]. Nevertheless, the specific mechanisms through which macrophages exert a pathological impact in these conditions are not fully elucidated [38]. In addition, they exhibit pro- or anti-inflammatory properties in response to various cytokines and microbial products [39]. Understanding the biological effects mediated by exosomes in terms of inducing anti-inflammatory or repressing pro-inflammatory cytokines is crucial for unraveling the cellular and molecular pathways that underlie the beneficial impact of these vesicles in humans. Exosomes were tested for their biological activity using RAW 264.7 macrophages. LPS was used as positive control on inflammation induction through the increase expression levels of *IL-1β*, *IL-6*, and *TNF-α* pro-inflammatory cytokines, cluster differentiation 80 (*CD80*) and *CD86*, and *IL-10*. Cells were cultured in the presence of exosomes, and the expression levels analyzed. IL-1β fosters intestinal stem-cell proliferation and offers protection in conditions like IBD. In addition, commensal bacteria promote IL-1β secretion to support intestinal barrier repair [40]. IL-6 has been shown to provide important survival and proliferative signals to many leukocyte populations and orchestrates the development of the immune response. IL-6 plays a crucial role in the development of B cell and T cell responses. Many edible plants contribute to maintaining intestinal immune-cell homeostasis. We investigated whether tepal exosomes stimulate immune molecules such as IL-1β and IL-6 to support homeostatic balance. Real-time PCR (Figure 4) results demonstrated that RAW 264.7 macrophages treated with exosomes showed a significant increase in IL-1β and IL-6 expression (Figure 4) after 2 h and 6 h of incubation, respectively, but the induction was at lower levels compared with the LPS treatment, which induced the expression by more than three orders of magnitude and showed a major induction after 12 and 8 h, respectively. The level of the pro-inflammatory cytokine TNF-α was also evaluated, due to its fundamental role in maintaining intestinal homeostasis. It is thought to perform crucial functions during both physiological and pathophysiological conditions in the gut [41]. Depending on TNF-α levels, it exhibits a dual role in cellular processes, including cell survival, differentiation, and proliferation by activating NFκB signaling. However, when this signaling pathway is inappropriately or excessively activated, it becomes linked to chronic inflammation, which may ultimately contribute to the development of pathological conditions, including autoimmune diseases, and the potential to induce cell death, including caspase-dependent apoptosis and caspase-independent programmed necrosis [42,43]. The treatment of macrophages with the exosomes induced the expression of *TNF-α*, with major levels reached 2 h after treatment. However, these levels were lower compared with the induction of *IL-1β* and *IL-6* by exosomes (Figure 4) and compared with levels induced by the LPS treatment, which reached a maximum 4 h after treatment. Next, we determine the expression levels of *CD80* and *CD86*. The stimulation of RAW264.7 cells with the exosomes increased the expression levels of *CD80* and *CD86*, but with a different time response for each one (Figure 4). *CD80* reached the highest levels 4 h after treatment, while *CD86* reached the highest expression levels 12 h after treatment. In both cases, the transcript levels were lower compared with the ones reached after LPS treatment (Figure 4). Finally, the expression of *IL-10* was also tested. The transcript levels of *IL-10* were highly induced 8 h after the treatment with LPS (Figure 4), while the exosomes were unable to induce a similar response (Figure 4).

## 4. Discussion

According to the UNEP Food Waste Index 2021, approximately 931 million tons of food waste were generated in 2019. Of this total, 61% originated from households, 26% from food service establishments, and 13% from the retail sector. These figures suggest that approximately 17% of global food production may be wasted at the household, food service, and retail stages of the food supply chain (https://www.unep.org/resources/, accessed on 13 February 2025). The opportunities provided by food-waste reduction have remained largely untapped and under-exploited. Incorporating technologies to derive value-added products from food waste, is crucial to keep the industry interested in the valorization strategies [44].

Saffron, renowned as the world’s most expensive spice, demands approximately 300,000 flowers to yield just 1 kg of dried stigma. Given its high cost and the scientific interest in the therapeutic potential of its bioactivities, exploring alternative plant parts, specifically industrial by-products like stamens and tepals, which are more abundant and cost-effective, emerges as a promising avenue. In this study, we successfully purified exosomes from saffron flower processing, and miRNAs, secondary metabolites, and proteins were analyzed. All of these are essential components of the extracellular vesicles. The unique composition of exosomes from each plant determines their bioactivity and functionalities, including the uptake, targeting, and modulation of gene expression. Further, exosome cargos are of unique tissue and cellular origins and contain specific miRNAs, proteins, and secondary metabolites [45]. Five major families of miRNAs were identified in the analyzed exosomes, including miR157, miR166, miR168, miR396, and miR398, which coordinate various growth and physiological plant responses with endogenous and environmental abiotic and biotic signals. Additionally, the identified miRNAs were scrutinized for their cross-kingdom regulatory capabilities. Among them, miR396 showed fifty possible targets, and five of these targets are upregulated in specific cancer types as renal, thyroid, prostate, and adrenocortical. MiR398 and miR168 were shown as possible targets genes implicated in glioma, while two of the potential miR156 targets were associated with liver and kidney cancer. In addition, in a recent work, MiR156 from ginseng significantly enhanced the proliferation capacity of macrophages, enhancing their phagocytosis ability and their immunomodulatory effect [46]. A study with the plant miR159 showed that the concentration of orally ingested miRNA was inversely related to the chances and progression of the breast cancer [47]. Previous work has shown that miR168 regulates mammalian LDLRAP1 translation in a fashion of mammalian functional miRNA, increasing plasma LDL level [48]. In *Fragaria vesca*, miR168 can reduce the inflammatory response in mouse, by decreasing the expression of TRIF transcript, which is required for innate immune responses mediated by TLR3 [49]. Interestingly, detectable levels of plant miR168 has been found in normal gastric, human feces, and colon cancer mucosa [50,51], suggesting potential interspecies activity. MiR166 was predicted to target high-affinity BCL2, IL2RA, and VAV1, with all of them being involved in the apoptosis, cell cycle, and immune response [52]. By other hand, miR396 can affect the expression of the CMKLR1 gene, involved in cardiovascular diseases, and the SYVN1 and SCAMP5 genes, changes in the expression of which are associated with colon cancer and autism, respectively [53]. In addition, miR396, derived from the astragalus root, regulates GATA-3 expression, and suppressed STAT-6 and ROR-γt mRNA in an asthma model of mice [54], and the miR396 presence in garlic exosomes indicates that miR-396e, one of the most prevalent miRNAs of GDE, played a crucial role in promoting the metabolic reprogramming of macrophage through PFKFB3 [55]. Thus, these miRNAs, by dietary intake, could target human genes and influence a range of immune- and metabolic-related biological pathways, including those associated with cancer [56,57].

In standard global proteomic experiments focused on exosomes, the identification of proteins can range from several hundreds to several thousands. This variability is influenced by different factors such as the initial material employed and the sample preparation methodology, among others [58]. In this study, Uniprot of *A. thaliana* proteins was utilized for protein annotation in the isolated exosomes, and 349 proteins were identified. To further delineate the functional relevance of these proteins, we conducted GO and pathway analysis (KEGG). The analysis showed an enrichment of proteasome proteins, and ribosomal proteins. In addition, proteins involved in the cytoskeleton and transport across the membrane (ABC transporters), also observed in the proteome of *Catharanthus roseus* exosomes [59] and vesicle trafficking, were identified. Among these proteins, we found RAB GTPases, which influence various aspects of membrane traffic and intracellular trafficking, influencing many cellular functions and responses, including roles in autophagy, plant microbe interactions, and in biotic and abiotic stress responses [60]; transmembrane 9 superfamily (TM9SF) proteins, known as Nonaspanins, which are present in several eukaryotic species such as mammals, insects, yeast, and plants, are essential in regulating the functions of lysosomes and endosomes, cellular adhesion, and for the phagocytosis of bacteria [61,62] and Coatomer subunits, also identified in human exosomes [63] that help in the formation and release of exosomes [64]. Besides these proteins, other proteins involved in stress-response processes, such as heat shock proteins, HSP70 and HSP90, which are major proteins found in exosomes [65,66], were also identified.

The floral residues of saffron contain several phenolic compounds, including flavonoids and anthocyanins as significant constituents, with kaempferol-3-*O*-sophoroside and delphinidin-3,5-di-*O*-glucoside being the predominant compounds, respectively [67,68], and a residual presence of crocins [69]. The analyses of the metabolites present in the exosomes revealed the presence of lutein and α- and β-carotene, together with delphinidin-3,5-di-*O*-glucoside and kaempferol-3,5-*O*-diglucoside, which all showed antioxidant and anti-inflammatory activities [70,71]. Exosomes from different plants showed the presence of characteristic secondary metabolites, which is the case for ginger exosomes, which are characterized by the presence of ginsenosides [72]; *Curcuma longa* exosomes contained curcumoids [36], while flavonoids such as naringin and naringenin have been identified in grapefruit exosomes [73].

Exosomes have been purified from many different plant sources and have demonstrated their therapeutic properties through in vitro and in vivo studies [74]. The biological activities and pharmacological functions of exosomes are similar to those reported in the original plants from which they were isolated, and are often more effective to those of a single active compound in plants [75]. This fact is attributed to their higher oral bioavailability and barrier permeability, and to the synergistic effect of the different cargo. Exosomes include a series of different cargos, secondary metabolites, proteins, and nucleic acids, which are protected from damage, and external stress by the lipid membrane. Thus, they remain stable through digestive and other biological fluids, and act as a vehicle for the cellular uptake of their cargo by the endocytosis.

Macrophages work as sentinels in the human body and are crucial for promptly responding to foreign stimuli. In the present study, the activation of the exosomes on macrophages was investigated using RAW264.7 macrophage cells as a cellular model. The exosomes significantly upregulated the mRNA expression levels of *TNF-α*, *IL-1β*, *IL-6*, and costimulatory molecules *CD80* and *CD86*, but at lower levels to the ones induced by LPS treatment. Macrophages usually differentiate into two major opposite phenotypes: M1 and M2. M1 macrophages are generally induced by microbial products, as LPS, interferon-gamma (IFN-γ), and TNF-α, and are associated with high antigen presentation abilities, high levels of IL-6 and IL-1β, the positive expression of CD80 and CD86, and low IL-10 levels, and are implicated in microbicidal, pro-inflammatory, and tumoricidal activities [76]. The optimum stimulation time for CD86 to be considered as an M1 polarization marker is 12 h, and their expression levels on M1 macrophages decreases thereafter [77]. This behavior can be observed after the treatment of RAW264.7 macrophages with the exosomes. Therefore, the obtained data suggest that the isolated exosomes promoted M1 macrophage polarization. In addition, the expression levels of *CD80* were increased in RAW264.7 macrophages by the exosomes, and CD80 has been shown to have a role in the immunosurveillance agent colorectal cancer in the early stages of tumorigenesis [78]. Similarly, exosomes isolated from rice bran [79], corn [80], ginseng [81], ginger [82], and *C. roseus* strongly stimulated the secretion and expression of *TNF-α*, at levels similar to LPS treatment, and activated the NF-κB signal pathway [59]. The anti-inflammatory activity of aqueous and ethanolic extracts of saffron tepals has been previously reported and attributed to anthocyanins, flavonoids, and other secondary metabolites present in the extracts [83]. Metabolomic analyses of the tepals’ exosomes confirmed the presence of only two classes of these compounds, kaempferol 3,7-diglucoside and delphinidin 3,5-di-O-glucoside, that together with their reduced levels in the exosomes suggested that the biological activity of the other cargo molecules present in the isolated exosomes could contribute to the observed activities.

## 5. Conclusions

The global annual production of saffron exceeds 220 tons, and nearly 190 tons constitute waste residue after flower processing, a considerable, inexpensive amount of by-product and a good source of exosomes. The nature of the biomolecules identified in these exosomes suggests that they could exert beneficial effects on humans and could be used in the food and nutraceutical industries. This indicates a potential medical application for exosomes in regulating fundamental biological processes in the human body, including the regulation of immune responses and delivering bioactive molecules. To demonstrate the potential of the isolated exosomes as nutraceutical tools, it is essential to assess the stability in biological fluids and evaluate the resistance to conditions that simulate the acidic gastric environment. New studies will evaluate the functional properties of these exosomes, with the goal of conclusively supporting their application in the nutraceutical and food industries.

## Figures and Tables

**Table 1:** miRNAs identified as cargo molecules in the exosomes isolated from waste saffron flowers.

ID	miRNA Database Pre-miRNA	miRNA Mature
*chr5_794*	Aquilegia caerulea miR156b stem-loop (aqc-MIR156b)	Populus trichocarpa miR156i stem-loop (ptc-MIR156i)
*chr5_1128*	Aquilegia caerulea miR156b stem-loop (aqc-MIR156b)	Populus trichocarpa miR156i stem-loop (ptc-MIR156i)
*chr5_2157*	Aquilegia caerulea miR156b stem-loop (aqc-MIR156b)	Populus trichocarpa miR156i stem-loop (ptc-MIR156i)
*chr5_2600*	Malus domestica miR398b stem-loop (mdm-MIR398b)	Nicotiana tabacum miR398 stem-loop (nta-MIR398)
*chr5_1183*	Asparagus officinalis miR398 stem-loop (aof-MIR398)	Nicotiana tabacum miR398 stem-loop (nta-MIR398)
*chr5_1177*	Asparagus officinalis miR398 stem-loop (aof-MIR398)	Nicotiana tabacum miR398 stem-loop (nta-MIR398)
*chr5_2342*	Gallus gallus (chicken) microRNA gga-mir-1599 precursor	
*chr5_1649*	Gossypium raimondii miR8641 stem-loop (gra-MIR8641)	
*chr4_1395*	Fragaria vesca miR396a stem-loop (fve-MIR396a)	Citrus sinensis miR396a stem-loop (csi-MIR396a)
*chr4_765*	Fragaria vesca miR396a stem-loop (fve-MIR396a)	Citrus sinensis miR396a stem-loop (csi-MIR396a)
*chr4_2266*	Fragaria vesca miR396a stem-loop (fve-MIR396a)	Citrus sinensis miR396a stem-loop (csi-MIR396a)
*chr4_2262*	Fragaria vesca miR396a stem-loop (fve-MIR396a)	Citrus sinensis miR396a stem-loop (csi-MIR396a)
*chr4_2115*	Fragaria vesca miR396a stem-loop (fve-MIR396a)	Citrus sinensis miR396a stem-loop (csi-MIR396a)
*chr4_1744*	Vriesea carinata miR166a stem-loop (vca-MIR166a)	Arabidopsis thaliana (thale cress) ath-miR166d
*chr4_1845*	Medicago truncatula miR2590e stem-loop (mtr-MIR2590e)	
*chr4_1747*	Medicago truncatula miR2676f stem-loop (mtr-MIR2676f)	
*chr4_566*	Brachypodium distachyon miR5058 stem-loop (bdi-MIR5058)	
*chr4_1330*	Manihot esculenta miR169ab stem-loop (mes-MIR169ab)	
*chr8_2423*	Citrus sinensis miR166c stem-loop (csi-MIR166c)	Aquilegia coerulea aqc-miR166a
*chr8_3304*	Manihot esculenta miR166a stem-loop (mes-MIR166a)	Aquilegia coerulea aqc-miR166a
*chr8_3423*	Petromyzon marinus (sea lamprey) microRNA pma-mir-22a precursor	Cucumis melo miR160c stem-loop (cme-MIR160c)
*chr3_1145*	Schistosoma mansoni microRNA sma-mir-2f precursor	
*chr3_952*	Ornithorhynchus anatinus (platypus) microRNA oan-mir-1347 precursor	
*chr8_2319*	Gallus gallus (chicken) microRNA gga-mir-1599 precursor	
*chr3_1017*	Oryctolagus cuniculus (rabbit) microRNA ocu-mir-3613 precursor	Oryza sativa (rice) osa-miR2275c
*chr3_1337*	Solanum lycopersicum miR396a stem-loop (sly-MIR396a)	
*chr8_2773*	Medicago truncatula miR168c stem-loop (mtr-MIR168c)	Arabidopsis thaliana (thale cress) ath-miR168a-3p
*chr8_3410*	Medicago truncatula miR5266 stem-loop (mtr-MIR5266)	
*chr7_3949*	Oryza sativa miR166d stem-loop (osa-MIR166d)	Arabidopsis thaliana (thale cress) ath-miR165a-3p
*chr7_3533*	Mus musculus (house mouse) microRNA mmu-mir-8109 precursor	
*chr2_1178*	Triticum aestivum miR5085 stem-loop (tae-MIR5085)	
*chr7_2915*	Gallus gallus (chicken) microRNA gga-mir-1599 precursor	
*chr7_2991*	Fragaria vesca miR162 stem-loop (fve-MIR162)	
*chr2_1335*	Lotus japonicus miR7526c stem-loop (lja-MIR7526c)	Citrus sinensis (sweet orange) csi-miR9560–5p
*chr2_776*	Tetraodon nigroviridis let-7a-1 stem-loop (tni-let-7a-1)	
*chr7_3878*	Gallus gallus (chicken) microRNA gga-mir-1599 precursor	
*chr2_725*	Medicago truncatula miR2670b stem-loop (mtr-MIR2670b)	
*chr2_2497*	Medicago truncatula miR2676f stem-loop (mtr-MIR2676f)	
*chr1_994*	Vriesea carinata miR396 stem-loop (vca-MIR396)	Arabidopsis thaliana (thale cress) ath-miR396b-5p
*chr1_2091*	Medicago truncatula miR168c stem-loop (mtr-MIR168c)	Cynara cardunculus miR168a stem-loop (cca-MIR168a)
*chr1_2089*	Medicago truncatula miR168c stem-loop (mtr-MIR168c)	Cynara cardunculus miR168a stem-loop (cca-MIR168a)
*chr1_636*	Medicago truncatula miR168c stem-loop (mtr-MIR168c)	Arabidopsis thaliana (thale cress) ath-miR168a-3p
*chr1_1326*	Brachypodium distachyon miR5058 stem-loop (bdi-MIR5058)	
*chr1_468*	Pan troglodytes (chimpanzee) microRNA ptr-mir-320e precursor	
*chr6_3204*	Mus musculus (house mouse) microRNA mmu-mir-7116 precursor	
*chr1_1961*	Mus musculus (house mouse) microRNA mmu-mir-7116 precursor	
*chr1_783*	Brassica napus miR824 stem-loop (bna-MIR824)	
*chr1_2624*	Brassica napus miR824 stem-loop (bna-MIR824)	

**Table 2:** Predicted target genes in the human genome of the five identified miRNAs.

miRNA	Gene ID, Name and Description
miR168	NM_001040429.3 Homo sapiens protocadherin 17 (PCDH17), mRNA
	NM_018644.3 Homo sapiens beta-1,3-glucuronyltransferase 1 (B3GAT1), transcript variant 1, mRNA
miR396	NM_001271562.2 Homo sapiens chromosome 11 open reading frame 71 (C11orf71), transcript variant
	NM_001370465.2 Homo sapiens dual specificity phosphatase 28 (DUSP28), transcript variant 1, mR
	NM_001394062.1 Homo sapiens microtubule actin crosslinking factor 1 (MACF1), transcript variant
	NM_001395392.1 Homo sapiens general transcription factor IIH subunit 2 (GTF2H2), transcript va variant
	NM_004736.4 Homo sapiens xenotropic and polytropic retrovirus receptor 1 (XPR1), transcript va variant
	NM_005502.4 Homo sapiens ATP binding cassette subfamily A member 1 (ABCA1), mRNA
	NM_006948.5 Homo sapiens heat shock protein family A (Hsp70) member 13 (HSPA13), mRNA
	NM_016052.4 Homo sapiens ribosomal RNA processing 15 homolog (RRP15), mRNA
	NM_022739.4 Homo sapiens SMAD specific E3 ubiquitin protein ligase 2 (SMURF2), mRNA
	NM_001004464.2 Homo sapiens olfactory receptor family 10 subfamily G member 8 (OR10G8), mRNA
	NM_001142934.2 Homo sapiens choline O-acetyltransferase (CHAT), transcript variant, mRNA
	NM_001270974.2 Homo sapiens HYDIN axonemal central pair apparatus protein (HYDIN), transcript
	NM_001308022.2 Homo sapiens tensin 1 (TNS1), transcript variant 2, mRNA
	NM_001321231.2 Homo sapiens histone deacetylase 6 (HDAC6), transcript variant 8, mRNA
	NM_001347701.2 Homo sapiens spectrin repeat containing nuclear envelope protein 1 (SYNE1)
	NM_001376194.2 Homo sapiens chromodomain helicase DNA binding protein 1 (CHD1)
	NM_002646.4 Homo sapiens phosphatidylinositol-4-phosphate 3-kinase catalytic subunit type 2
	NM_004371.4 Homo sapiens COPI coat complex subunit alpha (COPA), transcript variant 2, mRNA
	NM_004405.4 Homo sapiens distal-less homeobox 2 (DLX2), mRNA
	NM_004485.4 Homo sapiens G protein subunit gamma 4 (GNG4), transcript variant 3, mRNA
	NM_004664.4 Homo sapiens lin-7 homolog A, crumbs cell polarity complex component (LIN7A), transcript
	NM_007118.4 Homo sapiens trio Rho guanine nucleotide exchange factor (TRIO), transcript variant
	NM_007204.5 Homo sapiens DEAD-box helicase 20 (DDX20), mRNA
	NM_015114.3 Homo sapiens ankyrin repeat and LEM domain containing 2 (ANKLE2), mRNA
	NM_015286.6 Homo sapiens synemin (SYNM), transcript variant B, mRNA
	NM_016652.6 Homo sapiens crooked neck pre-mRNA splicing factor 1 (CRNKL1), transcript variant
	NM_018230.3 Homo sapiens nucleoporin 133 (NUP133), mRNA
	NM_022161.4 Homo sapiens baculoviral IAP repeat containing 7 (BIRC7), transcript variant 2, mRNA
	NM_001164183.2 Homo sapiens ectonucleoside triphosphate diphosphohydrolase 1 (ENTPD1), transcrIPT
	NM_001164389.2 Homo sapiens Rap guanine nucleotide exchange factor 6 (RAPGEF6), transcript variant
	NM_001166355.2 Homo sapiens O-fucosylpeptide 3-beta-N-acetylglucosaminyltransferase (LFNG)
	NM_001166699.2 Homo sapiens family with sequence similarity 104 member B (FAM104B), transcript
	NM_001286229.2 Homo sapiens denticleless E3 ubiquitin protein ligase homolog (DTL), transcript
	NM_001286365.2 Homo sapiens MAP7 domain containing 1 (MAP7D1), transcript variant 2, mRNA
	NM_001286730.2 Homo sapiens solute carrier family 44 member 1 (SLC44A1), transcript variant 2,
	NM_001301856.2 Homo sapiens ELOVL fatty acid elongase 5 (ELOVL5), transcript variant 5, mRNA
	NM_001310135.5 Homo sapiens tetratricopeptide repeat domain 6 (TTC6), transcript variant 1, mRNA
	NM_001318844.2 Homo sapiens SNW domain containing 1 (SNW1), transcript variant 1, mRNA
	NM_001324423.2 Homo sapiens lin-7 homolog A, crumbs cell polarity complex component (LIN7A), transcript varian t
	NM_001349745.2 Homo sapiens transmembrane and coiled-coil domains 3 (TMCO3), transcript varianT
	NM_001350339.2 Homo sapiens large tumor suppressor kinase 1 (LATS1), transcript variant 4, mRNA
	NM_001364113.3 Homo sapiens chromodomain helicase DNA binding protein 1 (CHD1), transcript varian
	NM_002788.4 Homo sapiens proteasome 20S subunit alpha 3 (PSMA3), transcript variant 1, mRNA
	NM_004274.5 Homo sapiens A-kinase anchoring protein 6 (AKAP6), mRNA
	NM_006428.5 Homo sapiens mitochondrial ribosomal protein L28 (MRPL28), mRNA
	NM_006469.5 Homo sapiens influenza virus NS1A binding protein (IVNS1ABP), mRNA
	NM_006836.2 Homo sapiens GCN1 activator of EIF2AK4 (GCN1), mRNA
	NM_012235.4 Homo sapiens SREBF chaperone (SCAP), transcript variant 1, mRNA
	NM_015488.5 Homo sapiens PNKD metallo-beta-lactamase domain containing (PNKD), transcript varian
	NM_015963.6 Homo sapiens THAP domain containing 4 (THAP4), transcript variant 1, mRNA
miR398	NM_001036.6 Homo sapiens ryanodine receptor 3 (RYR3), transcript variant 1, mRNA
	NM_001145784.2 Homo sapiens BLOC-1 related complex subunit 8 (BORCS8), transcript variant 1, mRNA
miR156	NM_001031848.2 Homo sapiens serpin family B member 8 (SERPINB8), transcript variant 3, mRNA
	NM_001144830.3 Homo sapiens flavin containing dimethylaniline monoxygenase 5 (FMO5), transcripT
	NM_001163771.2 Homo sapiens collagen type XI alpha 2 chain (COL11A2), transcript variant 4, mRNA
	NM_001243403.2 Homo sapiens C-type lectin domain containing 16A (CLEC16A), transcript variant
	NM_007021.4 Homo sapiens DEPP autophagy regulator 1 (DEPP1), mRNA
miR166	NM_015221.4 Homo sapiens dynamin binding protein (DNMBP), transcript variant 1, mRNA

**Table 3:** Metabolites identified as cargo molecules in the exosomes isolated from saffron-waste tepals. Metabolites found based on their *m*/*z*.

Type of Compound	Name	t*_R_*, min	*m*/*z*	Ion	Formula
Carotenoids					
	β-Carotene	7.00	537.4426	M + H^+^	C_40_H_56_
	α-Carotene	7.20	537.4426	M + H^+^	C_40_H_56_
	Zeaxanthin	4.73	569.4323	M + H^+^	C_40_H_56_O_2_
	Lutein	3.94	569.4323	M + H^+^	C_40_H_56_O_2_
	Hydroxy phytofluene	2.82	559.4901	M + H^+^	C_40_H_62_O
Quinones					
	Plastoquinone	6.25	748.6125	M + H^+^	C_53_H_80_O_2_
	Ubiquinone 10	5.78	862.6823	M + H^+^	C_59_H_90_O_4_
Flavonoids					
	Kaempferol 3,7-diglucoside	8.81	611.1604	M + H^+^	C_27_H_30_O_16_
Anthocyanins					
	Delphinidin 3,5-di-*O*-glucoside	8.36	627.5248	M^+^	C_15_H_11_O_7_

## Data Availability

The data supporting our findings are available in the manuscript file or from the corresponding author upon request. The datasets generated during the current study are available in the NCBI repository with number PRJNA1176498; all the proteomic data are included in the Supplementary Materials Section.

## Supplementary Materials

The following supporting information can be downloaded at: https://www.mdpi.com/article/10.3390/biology14020215/s1, Figure S1. Protein interactome network for the waste saffron exosome proteome using the STRING software (Version 12.0); Figure S2. Target metabolite analyses of saffron-waste tepals by HPLC-DAD analysis; Table S1. Primers used for the expression analyses; Table S2. Identified targets in *C. sativus* genome for the miRNAs detected in the exosomes; Table S3. Cross-kingdom regulatory capabilities of miRNAs identified in the saffron-waste tepals’ exosomes; Table S4. Proteins identified with homology to saffron proteins present in the NCBI database; Table S5. Relation of proteins identified in saffron flowers’ exosomes, using the Arabidopsis proteome; Table S6. Clusters of the 349 protein homologues to Arabidopsis identified in the exosomes; Table S7. The main significant pathways enriched in the proteome exosome.

## References

[1] Ben-Othman S., Jõudu I., Bhat R. (2020). Bioactives from Agri-Food Wastes: Present Insights and Future Challenges. Molecules.

[2] Oliveira T.C., Caleja C., Oliveira M.B.P., Pereira E., Barros L. (2023). Reuse of fruits and vegetables biowaste for sustainable development of natural ingredients. Food Biosci..

[3] Kalluri R., LeBleu V.S. (2020). The biology, function, and biomedical applications of exosomes. Science.

[4] Kumar M.A., Baba S.K., Sadida H.Q., Marzooqi S.A., Jerobin J., Altemani F.H., Algehainy N., Alanazi M.A., Abou-Samra A.-B., Kumar R. (2024). Extracellular vesicles as tools and targets in therapy for diseases. Signal Transduct. Target. Ther..

[5] Liu K., Chen Y.-Y., Pan L.-H., Li Q.-M., Luo J.-P., Zha X.-Q. (2022). Co-encapsulation systems for delivery of bioactive ingredients. Food Res. Int..

[6] Ashaolu T.J., Le T.-D., Suttikhana I. (2023). Stability and bioactivity of peptides in food matrices based on processing conditions. Food Res. Int..

[7] Mandal S. (2016). Curcumin, a promising anti-cancer therapeutic: It’s bioactivity and development of drug delivery vehicles. Int. J. Drug Res. Technol.

[8] Munir J., Lee M., Ryu S. (2020). Exosomes in Food: Health Benefits and Clinical Relevance in Diseases. Adv. Nutr. Int. Rev. J..

[9] Cho J.H., Hong Y.D., Kim D., Park S.J., Kim J.S., Kim H.-M., Yoon E.J., Cho J.-S. (2022). Confirmation of plant-derived exosomes as bioactive substances for skin application through comparative analysis of keratinocyte transcriptome. Appl. Biol. Chem..

[10] Ahrazem O., Rubio-Moraga A., Nebauer S.G., Molina R.V., Gómez-Gómez L. (2015). Saffron: Its Phytochemistry, Developmental Processes, and Biotechnological Prospects. J. Agric. Food Chem..

[11] Bukhari S.I., Manzoor M., Dhar M.K. (2018). A comprehensive review of the pharmacological potential of *Crocus sativus* and its bioactive apocarotenoids. Biomed. Pharmacother..

[12] Lahmass I., Lamkami T., Delporte C., Sikdar S., Van Antwerpen P., Saalaoui E., Megalizzi V. (2017). The waste of saffron crop, a cheap source of bioactive compounds. J. Funct. Foods.

[13] Ruggieri F., Maggi M.A., Rossi M., Consonni R. (2023). Comprehensive Extraction and Chemical Characterization of Bioactive Compounds in Tepals of *Crocus sativus* L. Molecules.

[14] Mottaghipisheh J., Sourestani M.M., Kiss T., Horváth A., Tóth B., Ayanmanesh M., Khamushi A., Csupor D. (2020). Comprehensive chemotaxonomic analysis of saffron crocus tepal and stamen samples, as raw materials with potential antidepressant activity. J. Pharm. Biomed. Anal..

[15] Zhou L., Cai Y., Yang L., Zou Z., Zhu J., Zhang Y. (2022). Comparative Metabolomics Analysis of Stigmas and Petals in Chinese Saffron (*Crocus sativus*) by Widely Targeted Metabolomics. Plants.

[16] Wang L., Qin Y., Wang Y., Zhou Y. (2024). Changes of anthocyanin and amino acid metabolites in saffron petals (*Crocus sativus* L.) during fermentation based on untargeted metabolomics. LWT.

[17] Hosseini A., Razavi B.M., Hosseinzadeh H. (2018). Saffron (*Crocus sativus*) petal as a new pharmacological target: A review. Iran. J. Basic Med. Sci..

[18] Moratalla-López N., Bagur M.J., Lorenzo C., Martínez-Navarro M.E., Salinas M.R., Alonso G.L. (2019). Bioactivity and Bioavailability of the Major Metabolites of *Crocus sativus* L. Flower. Molecules.

[19] Yao L., Guo S., Wang H., Feng T., Sun M., Song S., Hou F. (2022). Volatile fingerprints of different parts of Chongming saffron (*Crocus sativus*) flowers by headspace-gas chromatography-ion mobility spectrometry and in vitro bioactive properties of the saffron tepals. J. Food Sci..

[20] Ouahhoud S., Khoulati A., Kadda S., Bencheikh N., Mamri S., Ziani A., Baddaoui S., Eddabbeh F.-E., Lahmass I., Benabbes R. (2022). Antioxidant Activity, Metal Chelating Ability and DNA Protective Effect of the Hydroethanolic Extracts of *Crocus sativus* Stigmas, Tepals and Leaves. Antioxidants.

[21] Righi V., Parenti F., Tugnoli V., Schenetti L., Mucci A. (2015). *Crocus sativus* Petals: Waste or Valuable Resource? The Answer of High-Resolution and High-Resolution Magic Angle Spinning Nuclear Magnetic Resonance. J. Agric. Food Chem..

[22] Da Porto C., Natolino A. (2018). Extraction kinetic modelling of total polyphenols and total anthocyanins from saffron floral bio-residues: Comparison of extraction methods. Food Chem..

[23] Vardakas A., Vassilev K., Nenov N., Passon M., Shikov V., Schieber A., Mihalev K. (2024). Combining Enzymatic and Subcritical Water Treatments for Green Extraction of Polyphenolic Co-pigments from Saffron Tepals. Waste Biomass-Valorization.

[24] Lachguer K., El Merzougui S., Boudadi I., Laktib A., Ben El Caid M., Ramdan B., Boubaker H., Serghini M.A. (2023). Major Phytochemical Compounds, In Vitro Antioxidant, Antibacterial, and Antifungal Activities of Six Aqueous and Organic Extracts of Crocus sativus L. Flower Waste. Waste Biomass-Valorization.

[25] Naim N., Bouymajane A., El Majdoub Y.O., Ezrari S., Lahlali R., Tahiri A., Ennahli S., Vinci R.L., Cacciola F., Mondello L. (2022). Flavonoid Composition and Antibacterial Properties of *Crocus sativus* L. Petal Extracts. Molecules.

[26] Friedländer M.R., Mackowiak S.D., Li N., Chen W., Rajewsky N. (2012). miRDeep2 accurately identifies known and hundreds of novel microRNA genes in seven animal clades. Nucleic Acids Res..

[27] Bo X., Wang S. (2005). TargetFinder: A software for antisense oligonucleotide target site selection based on MAST and secondary structures of target mRNA. Bioinformatics.

[28] Diretto G., Ahrazem O., Rubio-Moraga Á., Fiore A., Sevi F., Argandoña J., Gómez-Gómez L. (2019). UGT709G1: A novel uridine diphosphate glycosyltransferase involved in the biosynthesis of picrocrocin, the precursor of safranal in saffron (*Crocus sativus*). New Phytol..

[29] Wu Y., Deng W., Ii D.J.K. (2015). Exosomes: Improved methods to characterize their morphology, RNA content, and surface protein biomarkers. Analyst.

[30] Pasquinelli A.E. (2012). MicroRNAs and their targets: Recognition, regulation and an emerging reciprocal relationship. Nat. Rev. Genet..

[31] Yadav A., Kumar S., Verma R., Lata C., Sanyal I., Rai S.P. (2021). microRNA 166: An evolutionarily conserved stress biomarker in land plants targeting HD-ZIP family. Physiol. Mol. Biol. Plants.

[32] Li Y., Li X., Yang J., He Y. (2020). Natural antisense transcripts of *MIR398* genes suppress microR398 processing and attenuate plant thermotolerance. Nat. Commun..

[33] Liebsch D., Palatnik J.F. (2020). MicroRNA miR396, GRF transcription factors and GIF co-regulators: A conserved plant growth regulatory module with potential for breeding and biotechnology. Curr. Opin. Plant Biol..

[34] Sim E., Abuhammad A., Ryan A. (2014). Arylamine N-acetyltransferases: From drug metabolism and pharmacogenetics to drug discovery. Br. J. Pharmacol..

[35] Suanno C., Tonoli E., Fornari E., Savoca M.P., Aloisi I., Parrotta L., Faleri C., Cai G., Coveney C., Boocock D.J. (2023). Small extracellular vesicles released from germinated kiwi pollen (pollensomes) present characteristics similar to mammalian exosomes and carry a plant homolog of ALIX. Front. Plant Sci..

[36] Woith E., Guerriero G., Hausman J.-F., Renaut J., Leclercq C.C., Weise C., Legay S., Weng A., Melzig M.F. (2021). Plant Extracellular Vesicles and Nanovesicles: Focus on Secondary Metabolites, Proteins and Lipids with Perspectives on Their Potential and Sources. Int. J. Mol. Sci..

[37] Dharmasiri S., Garrido-Martin E.M., Harris R.J., Bateman A.C., ECollins J., Cummings J.R.F., Sanchez-Elsner T. (2021). Human Intestinal Macrophages Are Involved in the Pathology of Both Ulcerative Colitis and Crohn Disease. Inflamm. Bowel Dis..

[38] Hegarty L.M., Jones G.-R., Bain C.C. (2023). Macrophages in intestinal homeostasis and inflammatory bowel disease. Nat. Rev. Gastroenterol. Hepatol..

[39] Mantovani A., Sica A., Locati M. (2007). New vistas on macrophage differentiation and activation. Eur. J. Immunol..

[40] Wu W.-J.H., Kim M., Chang L.-C., Assie A., Saldana-Morales F.B., Zegarra-Ruiz D.F., Norwood K., Samuel B.S., Diehl G.E. (2022). Interleukin-1β secretion induced by mucosa-associated gut commensal bacteria promotes intestinal barrier repair. Gut Microbes.

[41] Ruder B., Atreya R., Becker C. (2019). Tumour Necrosis Factor Alpha in Intestinal Homeostasis and Gut Related Diseases. Int. J. Mol. Sci..

[42] Zhao S., Jiang J., Jing Y., Liu W., Yang X., Hou X., Gao L., Wei L. (2020). The concentration of tumor necrosis factor-α determines its protective or damaging effect on liver injury by regulating Yap activity. Cell Death Dis..

[43] Jang D.-I., Lee A.-H., Shin H.-Y., Song H.-R., Park J.-H., Kang T.-B., Lee S.-R., Yang S.-H. (2021). The Role of Tumor Necrosis Factor Alpha (TNF-α) in Autoimmune Disease and Current TNF-α Inhibitors in Therapeutics. Int. J. Mol. Sci..

[44] Ravindran R., Jaiswal A.K. (2016). Exploitation of Food Industry Waste for High-Value Products. Trends Biotechnol..

[45] Kang T., Atukorala I., Mathivanan S. (2021). Biogenesis of Extracellular Vesicles. Subcell. Biochem..

[46] Wang J., Li C., Ruan J., Yang C., Tian Y., Lu B., Wang Y. (2024). Cross-kingdom regulation of ginseng miRNA156 on immunity and metabolism. Int. Immunopharmacol..

[47] Chin A.R., Fong M.Y., Somlo G., Wu J., Swiderski P., Wu X., Wang S.E. (2016). Cross-kingdom inhibition of breast cancer growth by plant miR159. Cell Res..

[48] Zhang L., Hou D., Chen X., Li D., Zhu L., Zhang Y., Bian Z., Liang X., Cai X., Yin Y. (2012). Exogenous plant MIR168a specifically targets mammalian LDLRAP1: Evidence of cross-kingdom regulation by microRNA. Cell Res..

[49] Kameli N., Dragojlovic-Kerkache A., Savelkoul P., Stassen F.R. (2021). Plant-Derived Extracellular Vesicles: Current Findings, Challenges, and Future Applications. Membranes.

[50] Cavalieri D., Rizzetto L., Tocci N., Rivero D., Asquini E., Si-Ammour A., Bonechi E., Ballerini C., Viola R. (2016). Plant microRNAs as novel immunomodulatory agents. Sci. Rep..

[51] Link J., Thon C., Schanze D., Steponaitiene R., Kupcinskas J., Zenker M., Canbay A., Malfertheiner P., Link A. (2019). Food-Derived Xeno-microRNAs: Influence of Diet and Detectability in Gastrointestinal Tract—Proof-of-Principle Study. Mol. Nutr. Food Res..

[52] Minutolo A., Potestà M., Roglia V., Cirilli M., Iacovelli F., Cerva C., Fokam J., Desideri A., Andreoni M., Grelli S. (2020). Plant microRNAs from Moringa oleifera Regulate Immune Response and HIV Infection. Front. Pharmacol..

[53] Rakhmetullina A., Pyrkova A., Aisina D., Ivashchenko A. (2020). In silico prediction of human genes as potential targets for rice miRNAs. Comput. Biol. Chem..

[54] Shen C.-B., Yu L., Wang S. (2018). miR-396 ameliorate allergic inflammation in a mouse model of asthma. J. Immunol..

[55] Bian Y., Li W., Jiang X., Yin F., Yin L., Zhang Y., Guo H., Liu J. (2023). Garlic-derived exosomes carrying miR-396e shapes macrophage metabolic reprograming to mitigate the inflammatory response in obese adipose tissue. J. Nutr. Biochem..

[56] Li D., Yang J., Yang Y., Liu J., Li H., Li R., Cao C., Shi L., Wu W., He K. (2021). A Timely Review of Cross-Kingdom Regulation of Plant-Derived MicroRNAs. Front. Genet..

[57] Alshehri B. (2021). Plant-derived xenomiRs and cancer: Cross-kingdom gene regulation. Saudi J. Biol. Sci..

[58] Doyle L., Wang M. (2019). Overview of Extracellular Vesicles, Their Origin, Composition, Purpose, and Methods for Exosome Isolation and Analysis. Cells.

[59] Ou X., Wang H., Tie H., Liao J., Luo Y., Huang W., Yu R., Song L., Zhu J. (2023). Novel plant-derived exosome-like nanovesicles from Catharanthus roseus: Preparation, characterization, and immunostimulatory effect via TNF-α/NF-κB/PU.1 axis. J. Nanobiotechnol..

[60] Tripathy M.K., Deswal R., Sopory S.K. (2021). Plant RABs: Role in Development and in Abiotic and Biotic Stress Responses. Curr. Genom..

[61] Xiao J., Shen X., Chen H., Ding L., Wang K., Zhai L., Mao C. (2022). TM9SF1 knockdown decreases inflammation by enhancing autophagy in a mouse model of acute lung injury. Heliyon.

[62] Perrin J., Le Coadic M., Vernay A., Dias M., Gopaldass N., Ouertatani-Sakouhi H., Cosson P. (2015). TM9 family proteins control surface targeting of glycine-rich transmembrane domains. J. Cell Sci..

[63] Conde-Vancells J., Rodriguez-Suarez E., Embade N., Gil D., Matthiesen R., Valle M., Elortza F., Lu S.C., Mato J.M., Falcon-Perez J.M. (2008). Characterization and comprehensive proteome profiling of exosomes secreted by hepatocytes. J. Proteome Res..

[64] Ghosh S., Ghosh S. (2022). Exosome: The “Off-the-Shelf” Cellular Nanocomponent as a Potential Pathogenic Agent, a Disease Biomarker, and Neurotherapeutics. Front. Pharmacol..

[65] Regimbeau M., Abrey J., Vautrot V., Causse S., Gobbo J., Garrido C. (2022). Heat shock proteins and exosomes in cancer theranostics. Semin. Cancer Biol..

[66] Komarova E.Y., Suezov R.V., Nikotina A.D., Aksenov N.D., Garaeva L.A., Shtam T.A., Zhakhov A.V., Martynova M.G., Bystrova O.A., Istomina M.S. (2021). Hsp70-containing extracellular vesicles are capable of activating of adaptive immunity in models of mouse melanoma and colon carcinoma. Sci. Rep..

[67] Serrano-Díaz J., Sánchez A.M., Martínez-Tomé M., Winterhalter P., Alonso G.L. (2014). Flavonoid Determination in the Quality Control of Floral Bioresidues from *Crocus sativus* L. J. Agric. Food Chem..

[68] Guijarro-Díez M., Nozal L., Marina M.L., Crego A.L. (2015). Metabolomic fingerprinting of saffron by LC/MS: Novel authenticity markers. Anal. Bioanal. Chem..

[69] Zeka K., Ruparelia K.C., Continenza M.A., Stagos D., Vegliò F., Arroo R.R. (2015). Petals of Crocus sativus L. as a potential source of the antioxidants crocin and kaempferol. Fitoterapia.

[70] Serafini M., Peluso I., Raguzzini A. (2010). Flavonoids as anti-inflammatory agents. Proc. Nutr. Soc..

[71] Sahoo D.K., Heilmann R.M., Paital B., Patel A., Yadav V.K., Wong D., Jergens A.E. (2023). Oxidative stress, hormones, and effects of natural antioxidants on intestinal inflammation in inflammatory bowel disease. Front. Endocrinol..

[72] Choi W., Cho J.H., Park S.H., Kim D.S., Lee H.P., Kim D., Kim H.S., Kim J.H., Cho J.Y. (2024). Ginseng root-derived exosome-like nanoparticles protect skin from UV irradiation and oxidative stress by suppressing activator protein-1 signaling and limiting the generation of reactive oxygen species. J. Ginseng Res..

[73] Wang B., Zhuang X., Deng Z.-B., Jiang H., Mu J., Wang Q., Xiang X., Guo H., Zhang L., Dryden G. (2014). Targeted Drug Delivery to Intestinal Macrophages by Bioactive Nanovesicles Released from Grapefruit. Mol. Ther..

[74] Dad H.A., Gu T.-W., Zhu A.-Q., Huang L.-Q., Peng L.-H. (2021). Plant Exosome-like Nanovesicles: Emerging Therapeutics and Drug Delivery Nanoplatforms. Mol. Ther..

[75] Yi Q., Xu Z., Thakur A., Zhang K., Liang Q., Liu Y., Yan Y. (2023). Current understanding of plant-derived exosome-like nanoparticles in regulating the inflammatory response and immune system microenvironment. Pharmacol. Res..

[76] Chen S., Saeed A.F., Liu Q., Jiang Q., Xu H., Xiao G.G., Rao L., Duo Y. (2023). Macrophages in immunoregulation and therapeutics. Signal Transduct. Target. Ther..

[77] Purcu D.U., Korkmaz A., Gunalp S., Helvaci D.G., Erdal Y., Dogan Y., Suner A., Wingender G., Sag D. (2022). Effect of stimulation time on the expression of human macrophage polarization markers. PLoS ONE.

[78] Scarpa M., Behboo R., Angriman I., Cecchetto A., D’incà R., Termini B., Barollo M., Ruffolo C., Polese L., Sturniolo G.C. (2006). Expression of costimulatory molecule CD80 in colonic dysplasia in ulcerative colitis: An immunosurveillance mechanism against colorectal cancer?. Int. J. Color. Dis..

[79] Sasaki D., Suzuki H., Kusamori K., Itakura S., Todo H., Nishikawa M. (2024). Development of rice bran-derived nanoparticles with excellent anti-cancer activity and their application for peritoneal dissemination. J. Nanobiotechnol..

[80] Sasaki D., Kusamori K., Takayama Y., Itakura S., Todo H., Nishikawa M. (2021). Development of nanoparticles derived from corn as mass producible bionanoparticles with anticancer activity. Sci. Rep..

[81] Cao M., Yan H., Han X., Weng L., Wei Q., Sun X., Lu W., Wei Q., Ye J., Cai X. (2019). Ginseng-derived nanoparticles alter macrophage polarization to inhibit melanoma growth. J. Immunother. Cancer.

[82] Mu J., Zhuang X., Wang Q., Jiang H., Deng Z.B., Wang B., Zhang L., Kakar S., Jun Y., Miller D. (2014). Interspecies communication between plant and mouse gut host cells through edible plant derived exosome-like nanoparticles. Mol. Nutr. Food Res..

[83] Shahi T., Assadpour E., Jafari S.M. (2016). Main chemical compounds and pharmacological activities of stigmas and tepals of ‘red gold’; saffron. Trends Food Sci. Technol..

