# Supplementary material for: Characterization of Exosome-like Nanoparticles from Saffron Tepals and Their Immunostimulatory Activity
Source: Biology (Basel). 2025 Feb 18;14(2):215. doi: 10.3390/biology14020215 (PMC11851917; doi:10.3390/biology14020215)
Supplement: Supplementary file 1 [file biology-14-00215-s001.zip › Supplemental Figures S1, S2, Tables S1, S3, S4, S6, S7.pdf]

Supplemental Materials:
Fig. S1
Table S1
Table S3
Table S4
Table S6
Table S7

Characterization of Exosome-like Nanoparticles from Saffron Tepals and Their Immunostimulatory Activity

Cristian Martínez Fajardo [1], Alberto J. López-Jiménez [1,2], Susana López-López [3,4], Lucía Morote [1], Elena Moreno-Giménez [1], Gianfranco Diretto [5], María José M. Díaz-Guerra [4], Ángela Rubio-Moraga [1,2], Oussama Ahrazem [1,2] and Lourdes Gómez-Gómez [1,6,*]

[1] Instituto Botánico, Departamento de Ciencia y Tecnología Agroforestal y Genética, Universidad de Castilla-La Mancha, Campus Universitario s/n, 02071 Albacete, Spain

[2] Escuela Técnica Superior de Ingenieros Agrónomos, Montes y Biotecnología, Departamento de Ciencia y Tecnología Agroforestal y Genética. Universidad de Castilla-La Mancha, Campus Universitario s/n, 02071 Albacete, Spain

[3] Unidad de Investigación, Complejo Hospitalario Universitario de Albacete, C/Laurel, s/n, 02008 Albacete, Spain

[4] Facultad de Medicina. Departamento de Química Inorgánica, orgánica y Bioquímica. Universidad de Castilla-La Mancha, Campus Universitario s/n, 02071 Albacete, Spain

[5] Italian National Agency for New Technologies, Energy, and Sustainable Development, Casaccia Research Centre, 00123 Rome, Italy

[6] Facultad de Farmacia. Departamento de Ciencia y Tecnología Agroforestal y Genética, Universidad de Castilla-La Mancha, Campus Universitario s/n, 02071 Albacete, Spain

* Corresponding author: marialourdes.gomez@uclm.es

Supplemental Figure S1

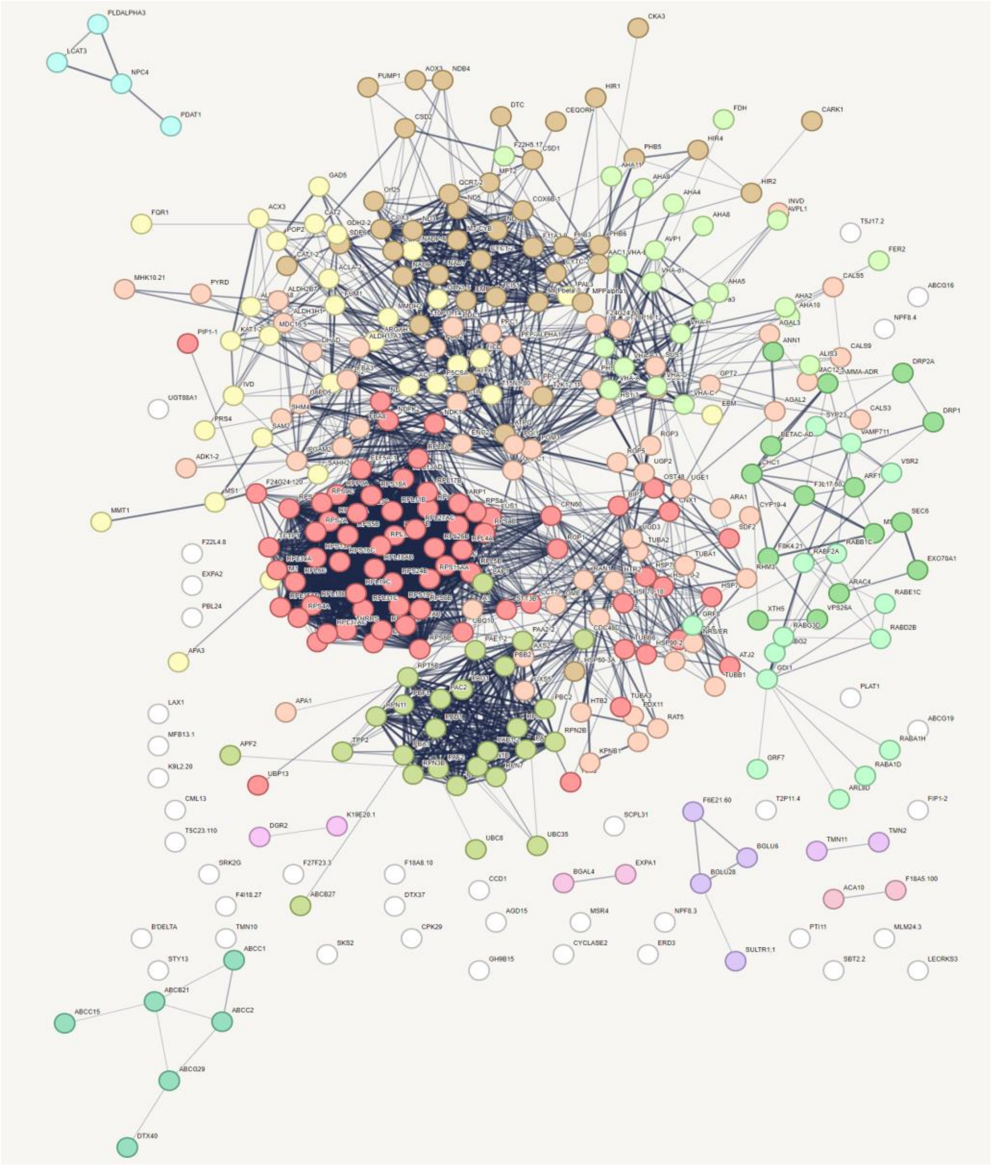

Figure S1. Protein interactome network for the waste saffron exosome proteome using the STRING software. The PPI network of identified proteins. Nodes represent proteins, while edges denote the interactions between 2 proteins. The PPI enrichment p-value was < 1.0-16 Stronger associations are represented by thicker lines. The k-means method was used because it separates proteins into distinct clusters. To determine the optimal number of clusters, we ran separate analyses with 10, 15, 20, and 25 clusters. The figure showed the results with 15 clusters.

Supplemental Figure S2

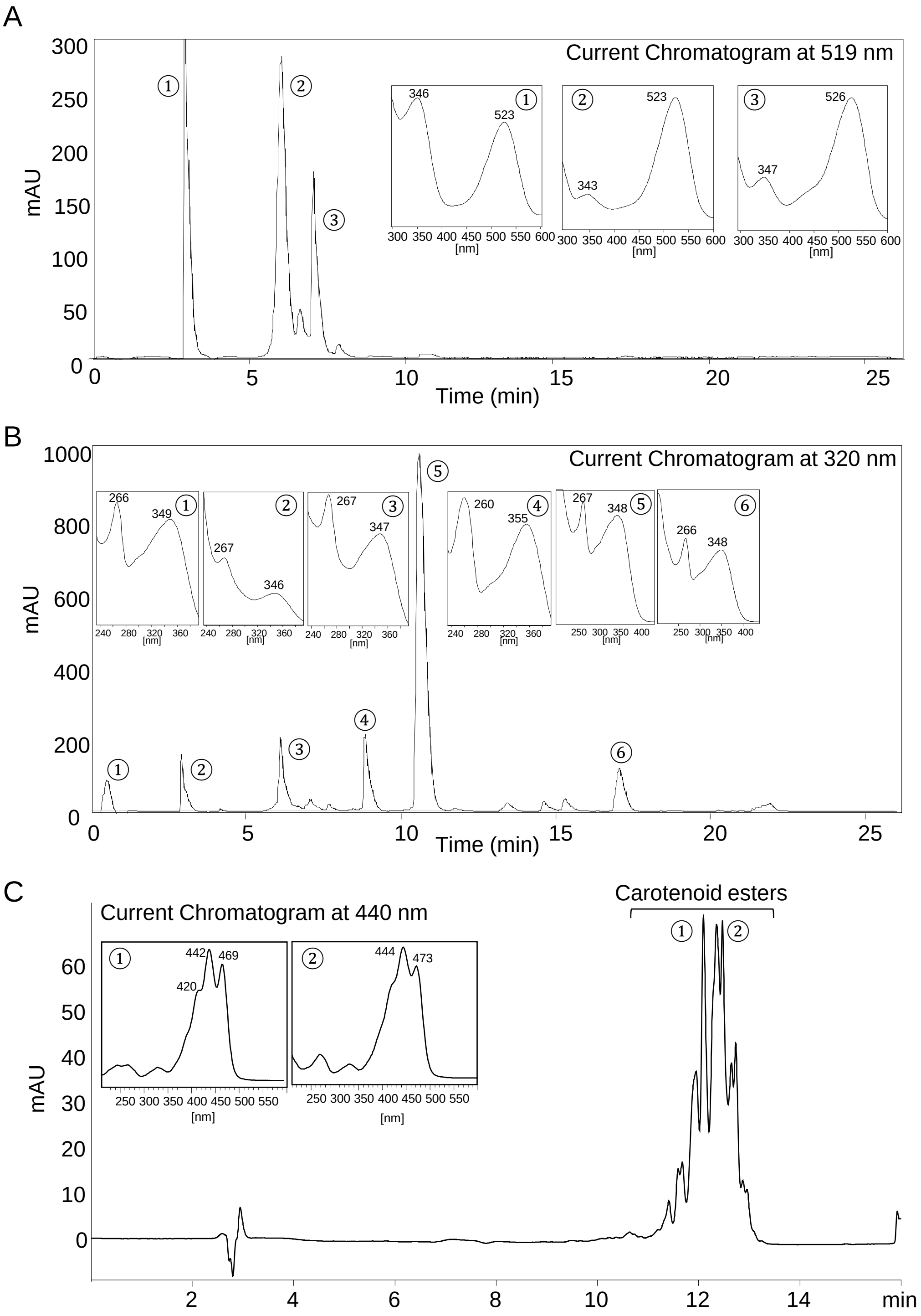

Figure S2. Target metabolite analyses of saffron waste tepals by HPLC-DAD analysis. A) Representative chromatogram of polar extracts at 519 nm for anthocyanins detection. 1, delphinidin 3,5-di-*O*-glucoside; 2, delphinidin 3-*O*-glucoside; 3, petunidin 3-*O*-glucoside. B) Representative chromatogram of polar extracts at 320 nm for flavonoids detection. 1, kaempferol 3-*O*-sophoroside-7-*O*-glucoside; 2, kaempferol 3,7-di-*O*-glucoside; 3, kaempferol 3-*O*-glucoside-7-*O*-rhamnoside; 4, quercetin 3-*O*-sophoroside; 5, kaempferol 3-*O*-sophoroside; 6, kaempferol 3-*O*-glucoside. C) Representative chromatogram of apolar extracts at 440 nm for carotenoids detection.

Table S1. Primers used for the expression analyses.

Gene	Forward Primer	Reverse Primer
P0	5′-GAATCGCTCCTGCAGCAAAG-3′	5′-CCAGGGTCTCATCCGCATT-3′
IL-6	5′-CCACGGCCTTCCCTACTTC-3′	5′-TTGGGAGTGGTATCCTCTGTGA-3′
CD80	5′-CAGGATTCGGCGCAGTAATAA-3′	5′-GTTTCTCTGCTTGCTCATTC-3′
IL-1β	5′-GGATGAGGACATGAGCACCT-3′	5′-AGCTCATATGGGTCCGACAG-3′
CD86	5′-CGACGTTTCCATCAGCTTGT-3′	5′-GGTGAAGATAAAAGCCGCGT-3′
Il-10	5′-CCTGGATCTGTATCACCGAAGC-3′	5′-CTCCGACCACTCTGCCTTGTTA-3′

Table S3. cross-kingdom regulatory capabilities of miRNAs identified in the saffron waste tepals exosomes.

Protein name	miRNA	pathology/disease	Data source: https://www.proteinatlas.org/search/
DNMBP	miR166	Chronic lymphocytic leukemia, Myeloma, Diffuse large B-cell lymphoma, Acute myeloid leukemia	
DEPP1	miR156	Cancer enhanced (Kidney cancer)	
CLEC16A	miR156	ND	
COL11A2	miR156	ND	
FMO5	miR156	Cancer enhanced (Liver cancer)	
SERPINB8	miR156	ND	
BORCS8	miR398	prostate cancers and cases of gliomas and melanomas.	
RYR3	miR398	ND	
PCDH17	miR168	Cancer enhanced (glioma)	
B3GAT1	miR168	Group enriched (glioma, prostate cancer, thyroid cancer)	
C11orf71	miR396	ND	
DUSP28	miR396	ND	
MACF1	miR396	ND	
GTF2H2	miR396	ND	
XPR1	miR396	ND	
ABCA1	miR396	ND	
HSPA13	miR396	ND	
RRP15	miR396	ND	
SMURF2	miR396	Cancer enhanced (Adrenocortical cancer)	
OR10G8	miR396	ND	
CHAT	miR396	ND	
HYDIN	miR396	ND	
TNS1	miR396	ND	
HDAC6	miR396	Detected in all cancer, low specificity	
SYNE1	miR396	Detected in all cancer, low specificity	
CHD1	miR396	Detected in all cancer, low specificity	
phosphatidylinositol-4-phosphate 3-kinase catalytic subunit type 2	miR396	Detected in all cancer, low specificity	
COPA	miR396	Detected in all cancer, low specificity	
DLX2	miR396	ND	
LIN7A	miR396	Cancer enhanced (renal)	
GNG4	miR396	ND	
DDX20	miR396	Detected in all cancer, low specificity	
TRIO	miR396	Detected in all cancer, low specificity	
ANKLE2	miR396	Detected in all cancer, low specificity	
SYNM	miR396	Cancer enhanced (prostate cancer)	
CRNKL1	miR396	ND	
NUP133	miR396	Detected in all cancer, low specificity	
BIRC7	miR396	ND	
ENTPD1	miR396	Cancer enhanced (thyroid cancer)	
RAPGEF6	miR396	Detected in all cancer, low specificity	
LFNG	miR396	Detected in all cancer, low specificity	
FAM104B	miR396	Detected in all cancer, low specificity	
DTL	miR396	Detected in all cancer, low specificity	
MAP7D1	miR396	Detected in all cancer, low specificity	
SLC44A1	miR396	Detected in all cancer, low specificity	
ELOVL5	miR396	Detected in all cancer, low specificity	
TTC6	miR396	ND	
SNW1	miR396	Detected in all cancer, low specificity	
LIN7A	miR396	Cancer enhanced (renal cancer)	
TMCO3	miR396	ND	
LATS1	miR396	Detected in all cancer, low specificity	
CHD1	miR396	Detected in all cancer, low specificity	
PSMA3	miR396	Detected in all cancer, low specificity	
AKAP6	miR396	Detected in all cancer, low specificity	
MRPL28	miR396	Detected in all cancer, low specificity	
IVNS1ABP	miR396	Detected in all cancer, low specificity	
GCN1	miR396	Detected in all cancer, low specificity	
SCAP	miR396	Detected in all cancer, low specificity	
PNKD	miR396	Detected in all cancer, low specificity	
THAP4	miR396	Detected in all cancer, low specificity	

Table S4. Proteins identified with homology to saffron proteins present in the NCBI data base.

Accession	Description	Score	Coverage	# Proteins	# Unique Peptides	# Peptides	# PSMs
A0A2U8ZTY0	Aldehyde dehydrogenase 2B4 OS=Crocus sativus OX=82528 GN=ALDH2B4 PE=2 SV=1 - [A0A2U8ZTY0_CROSA]	543,01	24,77	2	3	13	16
A0A3G1GZP7	Aldehyde dehydrogenase OS=Crocus sativus OX=82528 PE=2 SV=1 - [A0A3G1GZP7_CROSA]	499,81	23,84	1	2	12	14
A0A6H0C806	Carotenoid cleavage dioxygenase 1 OS=Crocus sativus OX=82528 GN=CCD1 PE=2 SV=1 - [A0A6H0C806_CROSA]	322,36	19,05	2	9	9	9
A0A2U8XEB8	Aldehyde dehydrogenase 11 OS=Crocus sativus OX=82528 PE=2 SV=1 - [A0A2U8XEB8_CROSA]	249,66	19,11	2	8	8	9
A0A5A4LPD2	ABC transporter C family member 2 OS=Crocus sativus OX=82528 GN=ABCC2 PE=2 SV=1 - [A0A5A4LPD2_CROSA]	246,67	5,84	2	7	7	8
A0A5J6NPR8	ATP-binding cassette sub-family C member 4b OS=Crocus sativus OX=82528 PE=2 SV=1 - [A0A5J6NPR8_CROSA]	242,67	5,65	3	1	7	7
A0A5J6NPW0	ATP-binding cassette sub-family C member 4c OS=Crocus sativus OX=82528 PE=2 SV=1 - [A0A5J6NPW0_CROSA]	213,35	5,51	3	1	7	7
D2T0A6	Non-specific lipid-transfer protein (Fragment) OS=Crocus sativus OX=82528 GN=LTP2 PE=2 SV=1 - [D2T0A6_CROSA]	195,24	44,57	1	4	4	5
A0A5J6ANM0	Aldehyde dehydrogenase OS=Crocus sativus OX=82528 GN=ALDH10A PE=2 SV=1 - [A0A5J6ANM0_CROSA]	169,21	13,69	1	6	6	6
A0A5J6NPU0	Protein DETOXIFICATION OS=Crocus sativus OX=82528 PE=2 SV=1 - [A0A5J6NPU0_CROSA]	156,55	4,80	1	2	2	2
A0A5K1KAX4	Aldehyde dehydrogenase OS=Crocus sativus OX=82528 GN=ALDH12A1 PE=2 SV=1 - [A0A5K1KAX4_CROSA]	137,40	10,97	1	5	5	5
A0A3G1GZN4	Aldehyde dehydrogenase OS=Crocus sativus OX=82528 PE=2 SV=1 - [A0A3G1GZN4_CROSA]	127,05	6,85	2	3	3	4
A0A411MRK4	Phenylalanine ammonia-lyase OS=Crocus sativus OX=82528 GN=PAL PE=2 SV=1 - [A0A411MRK4_CROSA]	115,97	4,47	1	2	2	2
A0A5A4LKD4	ABC transporter C family member 1 OS=Crocus sativus OX=82528 GN=ABCC1 PE=2 SV=1 - [A0A5A4LKD4_CROSA]	109,77	2,87	2	3	4	4
A0A5J6AM91	Aldehyde dehydrogenase OS=Crocus sativus OX=82528 GN=ALDH3F1 PE=2 SV=1 - [A0A5J6AM91_CROSA]	82,64	5,64	1	2	2	2
A0A343AXU3	Glycosyltransferase OS=Crocus sativus OX=82528 PE=2 SV=1 - [A0A343AXU3_CROSA]	59,34	2,52	1	1	1	1
A0A5A4LIX7	Protein DETOXIFICATION OS=Crocus sativus OX=82528 PE=2 SV=1 - [A0A5A4LIX7_CROSA]	48,32	2,20	1	1	1	1
A0A1S5VK40	Beta-glucosidase 12 OS=Crocus sativus OX=82528 PE=2 SV=1 - [A0A1S5VK40_CROSA]	48,03	3,75	1	2	2	2
A0A139YZN6	Glycosyltransferase OS=Crocus sativus OX=82528 GN=UGT703B1 PE=2 SV=1 - [A0A139YZN6_CROSA]	45,54	6,24	1	3	3	3
D2T0A5	Non-specific lipid-transfer protein (Fragment) OS=Crocus sativus OX=82528 GN=LTP1 PE=2 SV=1 - [D2T0A5_CROSA]	41,48	10,99	1	1	1	1
A0A481WAI5	ATP synthase subunit beta, chloroplastic OS=Crocus cartwrightianus OX=323707 GN=atpB PE=3 SV=1 - [A0A481WAI5_9ASPA]	40,00	2,21	1	1	1	1
A0A5A4LJP6	Protein DETOXIFICATION OS=Crocus sativus OX=82528 PE=2 SV=1 - [A0A5A4LJP6_CROSA]	36,67	2,25	3	1	1	1
A0A2U8XEY2	aldehyde dehydrogenase (NAD(+)) OS=Crocus sativus OX=82528 PE=2 SV=1 - [A0A2U8XEY2_CROSA]	35,18	2,17	1	1	1	1
A0A6J3UZ10	Glycosyltransferase (Fragment) OS=Crocus sativus OX=82528 GN=UGTCs4 PE=2 SV=1 - [A0A6J3UZ10_CROSA]	33,14	2,61	1	1	1	1
Q84K97	Alpha-tubulin (Fragment) OS=Crocus sativus OX=82528 PE=3 SV=1 - [Q84K97_CROSA]	31,03	10,67	1	1	1	1
A0A2U8XFS2	Succinate-semialdehyde dehydrogenase OS=Crocus sativus OX=82528 PE=2 SV=1 - [A0A2U8XFS2_CROSA]	30,47	2,90	1	1	1	1

Table S6. Clusters of the 349 proteins homologues to Arabidopsis identified in the exosomes. The k-means method was used because it separates proteins into distinct clusters. The analyses were done using the on-line tool: https://string-db.org/.

Clusters

bubble	cluster Id	gene count	protein names
	Cluster 1	73	ARP1, ATJ2, BIP2, CCT7, CNX1, CPN60, EIF4A1, ELF5A-1, F24G24.120, HSP70-18, HSP70-2, HSP70-3, HSP70-5, HSP90-2, HSP90-6, LOS1, NDK1, NDK4, NDPK2, OST48, PIP1-1, RACK1B, RGP1, RPL10A, RPL11B, RPL13AD, RPL13B, RPL17B, RPL18AB, RPL18AC, RPL18B, RPL19C, RPL23AA, RPL26B, RPL27AC, RPL31C, RPL35AD, RPL37AC, RPL39A, RPL4A, RPL5B, RPL6C, RPL7AB, RPL7B, RPL8A, RPL8C, RPP0A, RPS13A, RPS14C, RPS15AA, RPS16C, RPS18A, RPS19C, RPS23B, RPS24B, RPS2A, RPS3C, RPS4A, RPS5B, RPS6B, RPS8B, RPS9C, RPSaA, RPSaB, SDF2, STT3B, TCTP1, THRRS, TUBA3, TUBB6, UBP13, UBQ10, YSL8
	Cluster 2	63	A1, ACT4, ADK1-2, AGAL2, AGAL3, ALDH11A3, ALDH2B7, ALDH3H1, APA1, ARA1, AXS2, CALS3, CALS5, CALS9, CTIMC, CYP19-4, DHAD, ENO2, F24B9.25, F24G24.60, FBA3, FBA8, G6PD5, GAPC1, GME, GPT2, HTB2, HTR2, IMPA2, INVD, IPGAM2, KPNB1, MAC12.5, MHK10.21, NRS/ER, PDC1, PDX11, PFP-ALPHA1, PGD2, PGI1, PGM3, PHS1-3, PHS2, PKP1, PPC1, PPC3, PYRD, RAN1, RAT5, RGP3, RGP5, RHM3, SHM4, SUS1, SUS3, T28P16.12, TUBA1, TUBA2, TUBB1, UGD3, UGE1, UGP2, UXS5
	Cluster 3	42	AAC1, AOX3, ATPA, ATPQ, CARK1, CAT1-2, CEQORH, CI51, CKA3, COX2, COX3, COX6B-1, CSD1, CSD2, CYC1-2, CYTC-2, DTC, EMB1467, F11A3.9, HIR1, HIR2, HIR4, HSP60-3A, MPPalpha1, MPPbeta, MPT2, MT-CYB, NAD7, NAD9, ND1, ND3, ND5, NDB4, Orf25, PHB3, PHB5, PHB6, PUMP1, QCR7-2, SDP6, T10P11.14, T2K12.11
	Cluster 4	31	ACLA-2, ACLB-2, ACO1, ACX3, ALDH10A8, APA3, APM1, ARGAH1, CAT2, EBM, FQR1, FUM1, GAD5, GDH2-2, GLN1-5, ICDH, IVD, KAT1-2, MDC16.5, MDH1, MMDH2, MMT1, MS1, NADP-ME4, P5CSA, PAL3, POP2, PRS4, SAHH2, SAM2, T15N1.80
	Cluster 5	27	ABCB27, APF2, CDC48D, LAP1, PAA2-2, PAB1-2, PAC2, PAD2, PAE1-2, PAF2, PAG1, PBA1, PBB2, PBC2, PBD1, PBE2, PBF1, RPN11, RPN1B, RPN2B, RPN3B, RPN6, RPN7, RPT5B, TPP2, UBC35, UBC8
	Cluster 6	22	AHA10, AHA11, AHA2, AHA4, AHA5, AHA8, AHA9, ALIS3, AVP1, AVPL1, F22H5.17, FDH, FER2, PPA4, VHA-A, VHA-B2, VHA-C, VHA-D, VHA-E3, VHA-H, VHA-a3, VHA-d1
	Cluster 7	15	ANN1, ARAC4, ARF1, BETAC-AD, CHC1, DRP1A, DRP2A, EXO70A1, F3L17.60, F8K4.21, GAMMA-ADR, MSL1.2, SEC6, VPS26A, XTH5
	Cluster 8	15	ARL8D, GDI1, GRF7, GRF8, RABA1D, RABA1H, RABB1C, RABD2B, RABE1C, RABF2A, RABG2, RABG3D, SYP23, VAMP711, VSR2
	Cluster 9	6	ABCB21, ABCC1, ABCC15, ABCC2, ABCG29, DTX40
	Cluster 10	4	LCAT3, NPC4, PDAT1, PLDALPHA3
	Cluster 11	4	BGLU28, BGLU6, F6E21.60, SULTR1;1
	Cluster 12	2	TMN11, TMN2
	Cluster 13	2	DGR2, K19E20.1
	Cluster 14	2	BGAL4, EXPA1
	Cluster 15	2	ACA10, F18A5.100

Table S7. Main significant pathways enriched in the proteome exosome.

pathway	description	count in network	strength	false discovery rate
ath03050	Proteasome	20 of 60	1.42	3.10e-19
ath00903	Limonene and pinene degradation	2 of 6	1.42	0.0208
ath00190	Oxidative phosphorylation	30 of 146	1.21	1.31e-23
ath04145	Phagosome	16 of 77	1.21	7.87e-13
ath00603	Glycosphingolipid biosynthesis - globo and isoglobo series	2 of 11	1.16	0.0425
ath00020	Citrate cycle (TCA cycle)	9 of 62	1.06	2.87e-06
ath00030	Pentose phosphate pathway	8 of 58	1.04	1.52e-05
ath03010	Ribosome	42 of 318	1.02	2.80e-26
ath00710	Carbon fixation in photosynthetic organisms	9 of 69	1.01	5.59e-06
ath00010	Glycolysis / Gluconeogenesis	15 of 118	1.0	2.05e-09
ath00520	Amino sugar and nucleotide sugar metabolism	16 of 131	0.98	9.57e-10
ath00620	Pyruvate metabolism	10 of 86	0.96	3.74e-06
ath00410	beta-Alanine metabolism	5 of 47	0.92	0.0031
ath01200	Carbon metabolism	26 of 270	0.88	2.05e-13
ath00051	Fructose and mannose metabolism	6 of 62	0.88	0.0014
ath04146	Peroxisome	8 of 85	0.87	0.00018
ath00592	alpha-Linolenic acid metabolism	4 of 43	0.87	0.0152
ath00630	Glyoxylate and dicarboxylate metabolism	7 of 77	0.86	0.00064
ath04144	Endocytosis	14 of 161	0.84	6.05e-07
ath00052	Galactose metabolism	5 of 57	0.84	0.0066
ath00770	Pantothenate and CoA biosynthesis	3 of 34	0.84	0.0425
ath00071	Fatty acid degradation	4 of 47	0.83	0.0197
ath00220	Arginine biosynthesis	3 of 36	0.82	0.0452
ath00250	Alanine, aspartate and glutamate metabolism	4 of 51	0.79	0.0240
ath00561	Glycerolipid metabolism	5 of 65	0.78	0.0109
ath00280	Valine, leucine and isoleucine degradation	4 of 52	0.78	0.0247
ath00330	Arginine and proline metabolism	4 of 54	0.77	0.0271
ath00053	Ascorbate and aldarate metabolism	4 of 54	0.77	0.0271
ath01230	Biosynthesis of amino acids	17 of 241	0.75	4.41e-07
ath00500	Starch and sucrose metabolism	12 of 170	0.75	2.93e-05
ath00240	Pyrimidine metabolism	4 of 60	0.72	0.0358
ath00380	Tryptophan metabolism	4 of 62	0.71	0.0375
ath00230	Purine metabolism	6 of 100	0.68	0.0124
ath04141	Protein processing in endoplasmic reticulum	12 of 212	0.65	0.00021
ath00260	Glycine, serine and threonine metabolism	4 of 70	0.65	0.0483
ath01100	Metabolic pathways	117 of 2285	0.61	1.27e-37
ath00480	Glutathione metabolism	5 of 102	0.59	0.0425
ath01110	Biosynthesis of secondary metabolites	58 of 1219	0.57	3.63e-16
ath04016	MAPK signaling pathway - plant	6 of 136	0.54	0.0372
