# Supplementary material for: Characterization of Exosome-like Nanoparticles from Saffron Tepals and Their Immunostimulatory Activity
Source: Biology (Basel). 2025 Feb 18;14(2):215. doi: 10.3390/biology14020215 (PMC11851917; doi:10.3390/biology14020215)
Supplement: Supplementary file 1 [file biology-14-00215-s001.zip › Table S2.pdf]

Table S2.

SeqName	#Hits	Length	Description	e-Value	sim mean
			**MiR157**		
Csativus00037.1	5	1080	A0A2H3YB86_PHODCprobable feruloyl esterase A OS=Phoenix dactylifera OX=42345 GN=LOC103711735 PE=4 SV=1	0	8701
Csativus03063.1	5	954	A0A2H3YZY0_PHODCsquamosa promoter-binding-like protein 16 OS=Phoenix dactylifera OX=42345 GN=LOC10371	2,3392E-66	5875
Csativus03067.1	5	945	A0A2H3XYQ2_PHODCsquamosa promoter-binding-like protein 16 OS=Phoenix dactylifera OX=42345 GN=LOC1037	4,3888E-68	5953
Csativus03077.1	5	951	A0A2H3YZY0_PHODCsquamosa promoter-binding-like protein 16 OS=Phoenix dactylifera OX=42345 GN=LOC10371	3,9088E-64	5724
Csativus08221.1	5	1020	A0A2H3YAS6_PHODCsquamosa promoter-binding-like protein 14 isoform X1 OS=Phoenix dactylifera OX=42345 GN	1,015E-102	622
Csativus08226.1	5	1014	A0A2H3YA40_PHODCsquamosa promoter-binding-like protein 14 isoform X2 OS=Phoenix dactylifera OX=42345 GN	3,854E-104	6374
Csativus14861.1	5	1242	A0A5P1EU73_ASPOFSBP-type domain-containing protein OS=Asparagus officinalis OX=4686 GN=A4U43_C05F6790	8,346E-163	6901
Csativus14873.1	5	1227	A0A5P1EU73_ASPOFSBP-type domain-containing protein OS=Asparagus officinalis OX=4686 GN=A4U43_C05F6790	3,406E-162	6831
Csativus15553.1	5	1080	A0A2H3X008_PHODCsquamosa promoter-binding-like protein 14 OS=Phoenix dactylifera OX=42345 GN=LOC10369	2,415E-113	667
Csativus15612.1	5	1080	A0A2H3X008_PHODCsquamosa promoter-binding-like protein 14 OS=Phoenix dactylifera OX=42345 GN=LOC10369	2,938E-113	6619
Csativus16674.1	5	834	A0A2H3YZY0_PHODCsquamosa promoter-binding-like protein 16 OS=Phoenix dactylifera OX=42345 GN=LOC10371	1,0879E-72	546
Csativus18443.1	5	1164	A0A2H3YPU0_PHODCsquamosa promoter-binding-like protein 16 OS=Phoenix dactylifera OX=42345 GN=LOC10371	2,518E-112	6882
Csativus18627.1	5	1581	A0A2H3YUQ3_PHODCWRKY transcription factor WRKY24-like OS=Phoenix dactylifera OX=42345 GN=LOC103717512	0	6998
Csativus18652.1	5	1584	A0A2H3YUQ3_PHODCWRKY transcription factor WRKY24-like OS=Phoenix dactylifera OX=42345 GN=LOC103717512	0	703
Csativus19250.1	5	924	A0A2H3YZY0_PHODCsquamosa promoter-binding-like protein 16 OS=Phoenix dactylifera OX=42345 GN=LOC10371	3,9312E-74	5659
Csativus23385.1	5	1158	A0A2H3YPU0_PHODCsquamosa promoter-binding-like protein 16 OS=Phoenix dactylifera OX=42345 GN=LOC10371	4,933E-123	7074
Csativus25932.1	5	1164	A0A2H3YPU0_PHODCsquamosa promoter-binding-like protein 16 OS=Phoenix dactylifera OX=42345 GN=LOC10371	8,529E-137	7273
Csativus28623.1	5	1350	A0A5P1EU73_ASPOFSBP-type domain-containing protein OS=Asparagus officinalis OX=4686 GN=A4U43_C05F6790	1,363E-154	6919
Csativus29035.1	5	1032	A0A2H3XF46_PHODCsquamosa promoter-binding-like protein 17 OS=Phoenix dactylifera OX=42345 GN=LOC10370	4,588E-108	6535
Csativus29043.1	5	1023	A0A2H3XF46_PHODCsquamosa promoter-binding-like protein 17 OS=Phoenix dactylifera OX=42345 GN=LOC10370	3,212E-111	6565
Csativus29917.1	5	594	A0A5P1EU73_ASPOFSBP-type domain-containing protein OS=Asparagus officinalis OX=4686 GN=A4U43_C05F6790	4,8241E-61	6224
Csativus29991.1	5	1332	A0A5P1EU73_ASPOFSBP-type domain-containing protein OS=Asparagus officinalis OX=4686 GN=A4U43_C05F6790	1,134E-155	6811
Csativus31551.1	5	1014	A0A2H3XF46_PHODCsquamosa promoter-binding-like protein 17 OS=Phoenix dactylifera OX=42345 GN=LOC10370	7,447E-109	654
Csativus38153.1	5	1080	A0A2H3YB86_PHODCprobable feruloyl esterase A OS=Phoenix dactylifera OX=42345 GN=LOC103711735 PE=4 SV=1	0	8683
Csativus41201.1	5	1164	A0A2H3YPU0_PHODCsquamosa promoter-binding-like protein 16 OS=Phoenix dactylifera OX=42345 GN=LOC10371	1,585E-119	6876
Csativus44317.1	5	1122	A0A2H3YPU0_PHODCsquamosa promoter-binding-like protein 16 OS=Phoenix dactylifera OX=42345 GN=LOC10371	1,293E-118	7162
Csativus44331.1	5	996	A0A5P1E7A2_ASPOFSBP-type domain-containing protein OS=Asparagus officinalis OX=4686 GN=A4U43_C09F1375	1,0392E-68	6734
Csativus44335.1	5	1125	A0A2H3YPU0_PHODCsquamosa promoter-binding-like protein 16 OS=Phoenix dactylifera OX=42345 GN=LOC10371	1,072E-115	7144
Csativus50457.1	5	1191	A0A2H3YPU0_PHODCsquamosa promoter-binding-like protein 16 OS=Phoenix dactylifera OX=42345 GN=LOC10371	4,6529E-97	6941
Csativus55086.1	5	810	A0A2H3XYQ2_PHODCsquamosa promoter-binding-like protein 16 OS=Phoenix dactylifera OX=42345 GN=LOC1037	2,5764E-55	5706
			**MiR166**		
Csativus02984.1	5	2544	I0IUI5_ASPOFClass III homeobox-leucine zipper protein OS=Asparagus officinalis OX=4686 GN=AoPHB PE=2 SV=1	0	9207
Csativus03046.1	5	2544	I0IUI5_ASPOFClass III homeobox-leucine zipper protein OS=Asparagus officinalis OX=4686 GN=AoPHB PE=2 SV=1	0	9198
Csativus16625.1	5	2553	I0IUI5_ASPOFClass III homeobox-leucine zipper protein OS=Asparagus officinalis OX=4686 GN=AoPHB PE=2 SV=1	0	9173
Csativus16628.1	5	2556	I0IUI5_ASPOFClass III homeobox-leucine zipper protein OS=Asparagus officinalis OX=4686 GN=AoPHB PE=2 SV=1	0	9128
Csativus16658.1	5	2556	I0IUI5_ASPOFClass III homeobox-leucine zipper protein OS=Asparagus officinalis OX=4686 GN=AoPHB PE=2 SV=1	0	9108
Csativus19299.1	5	1752	A0A2H3Y776_PHODChomeobox-leucine zipper protein HOX32 OS=Phoenix dactylifera OX=42345 GN=LOC1037105	0	9161
Csativus19323.1	5	2595	I0IUI5_ASPOFClass III homeobox-leucine zipper protein OS=Asparagus officinalis OX=4686 GN=AoPHB PE=2 SV=1	0	9139
Csativus19883.1	5	2526	A0A2H3Y0I2_PHODChomeobox-leucine zipper protein ATHB-15-like OS=Phoenix dactylifera OX=42345 GN=LOC10	0	8771
Csativus19891.1	5	2532	A0A2H3Y0I2_PHODChomeobox-leucine zipper protein ATHB-15-like OS=Phoenix dactylifera OX=42345 GN=LOC10	0	8796
Csativus23595.1	5	3003	A0A2H3X0G7_PHODCfilament-like plant protein 4 isoform X1 OS=Phoenix dactylifera OX=42345 GN=LOC10369658	0	669
Csativus24025.1	5	2547	A0A2H3Y0E5_PHODChomeobox-leucine zipper protein HOX9-like isoform X1 OS=Phoenix dactylifera OX=42345 GN	0	8828
Csativus27994.1	5	3003	A0A2H3X0G7_PHODCfilament-like plant protein 4 isoform X1 OS=Phoenix dactylifera OX=42345 GN=LOC10369658	0	6631
Csativus28349.1	5	2538	I0IUI5_ASPOFClass III homeobox-leucine zipper protein OS=Asparagus officinalis OX=4686 GN=AoPHB PE=2 SV=1	0	9149
Csativus28359.1	5	2538	I0IUI5_ASPOFClass III homeobox-leucine zipper protein OS=Asparagus officinalis OX=4686 GN=AoPHB PE=2 SV=1	0	9149
Csativus29874.1	5	1023	A0A2H3ZQC5_PHODChomeobox-leucine zipper protein HOX9-like isoform X2 OS=Phoenix dactylifera OX=42345 G	3,234E-117	6864
Csativus40502.1	5	2505	A0A2H3Y0E5_PHODChomeobox-leucine zipper protein HOX9-like isoform X1 OS=Phoenix dactylifera OX=42345 GN	0	8792
Csativus40509.1	5	2505	A0A2H3Y0E5_PHODChomeobox-leucine zipper protein HOX9-like isoform X1 OS=Phoenix dactylifera OX=42345 GN	0	8824
Csativus42228.1	5	2538	A0A2H3Y0E5_PHODChomeobox-leucine zipper protein HOX9-like isoform X1 OS=Phoenix dactylifera OX=42345 GN	0	8759
Csativus47642.1	5	1014	A0A2H3ZQC5_PHODChomeobox-leucine zipper protein HOX9-like isoform X2 OS=Phoenix dactylifera OX=42345 G	1,201E-151	8686
Csativus51333.1	5	2505	A0A2H3Y0E5_PHODChomeobox-leucine zipper protein HOX9-like isoform X1 OS=Phoenix dactylifera OX=42345 GN	0	8804
Csativus51338.1	5	2493	A0A2H3Y0E5_PHODChomeobox-leucine zipper protein HOX9-like isoform X1 OS=Phoenix dactylifera OX=42345 GN	0	878
Csativus52681.1	5	1821	A0A5P1E519_ASPOFPhosphatidylserine decarboxylase proenzyme 2 OS=Asparagus officinalis OX=4686 GN=PSD2 P	0	8836
Csativus54198.1	5	2553	I0IUI5_ASPOFClass III homeobox-leucine zipper protein OS=Asparagus officinalis OX=4686 GN=AoPHB PE=2 SV=1	0	927
Csativus54220.1	5	2553	I0IUI5_ASPOFClass III homeobox-leucine zipper protein OS=Asparagus officinalis OX=4686 GN=AoPHB PE=2 SV=1	0	9284
Csativus55061.1	5	2547	I0IUI5_ASPOFClass III homeobox-leucine zipper protein OS=Asparagus officinalis OX=4686 GN=AoPHB PE=2 SV=1	0	9316
Csativus58306.1	5	2541	A0A2H3Y0E5_PHODChomeobox-leucine zipper protein HOX9-like isoform X1 OS=Phoenix dactylifera OX=42345 GN	0	8697
			**MiR168**		
Csativus02153.1	5	1875	GLO14_ORYSJVery-long-chain aldehyde decarbonylase GL1-4 OS=Oryza sativa subsp. japonica OX=39947 GN=GL1-4	0	8072
Csativus45054.1	5	1515	A0A2H3YA49_PHODCEndoglucanase OS=Phoenix dactylifera OX=42345 GN=LOC103711412 PE=3 SV=1	0	9069
Csativus46759.1	5	2961	A0A5P1EVJ0_ASPOFExocyst subunit Exo70 family protein OS=Asparagus officinalis OX=4686 GN=A4U43_C05F28260	0	8498
Csativus02153.1	5	1875	GLO14_ORYSJVery-long-chain aldehyde decarbonylase GL1-4 OS=Oryza sativa subsp. japonica OX=39947 GN=GL1-4	0	8072
Csativus45054.1	5	1515	A0A2H3YA49_PHODCEndoglucanase OS=Phoenix dactylifera OX=42345 GN=LOC103711412 PE=3 SV=1	0	9069
Csativus46759.1	5	2961	A0A5P1EVJ0_ASPOFExocyst subunit Exo70 family protein OS=Asparagus officinalis OX=4686 GN=A4U43_C05F28260	0	8498
			**MiR396**		
Csativus00052.1	5	756	A0A5P1FAZ4_ASPOFRRM domain-containing protein OS=Asparagus officinalis OX=4686 GN=A4U43_C03F17950 PE=	5,7664E-60	9762
Csativus00824.1	1	1713	A0A5P1EYW3_ASPOFRep_fac-A_C domain-containing protein OS=Asparagus officinalis OX=4686 GN=A4U43_C04F4	1,4037E-28	4512
Csativus01640.1	5	555	A0A2H3ZEW0_PHODCGrowth-regulating factor OS=Phoenix dactylifera OX=42345 GN=LOC103723579 PE=3 SV=1	1,6851E-62	8613
Csativus01678.1	5	993	A0A2H3ZEW0_PHODCGrowth-regulating factor OS=Phoenix dactylifera OX=42345 GN=LOC103723579 PE=3 SV=1	2,853E-125	6858
Csativus01705.1	5	993	A0A2H3ZGE7_PHODCGrowth-regulating factor OS=Phoenix dactylifera OX=42345 GN=LOC103724096 PE=3 SV=1	1,055E-122	7183
Csativus02717.1	5	2763	A0A5P1FNL3_ASPOFLipoxygenase OS=Asparagus officinalis OX=4686 GN=A4U43_C01F2120 PE=3 SV=1	0	7939
Csativus02718.1	5	2763	A0A5P1FNL3_ASPOFLipoxygenase OS=Asparagus officinalis OX=4686 GN=A4U43_C01F2120 PE=3 SV=1	0	8023
Csativus02723.1	5	2763	A0A5P1FNL3_ASPOFLipoxygenase OS=Asparagus officinalis OX=4686 GN=A4U43_C01F2120 PE=3 SV=1	0	7948
Csativus02738.1	5	2763	A0A5P1FNL3_ASPOFLipoxygenase OS=Asparagus officinalis OX=4686 GN=A4U43_C01F2120 PE=3 SV=1	0	7965
Csativus03498.1	5	2334	A0A5P1EID3_ASPOFComponent of oligomeric Golgi complex 3 OS=Asparagus officinalis OX=4686 GN=A4U43_C07F	0	8987
Csativus03746.1	5	3123	A0A2H3ZYC6_PHODCmyosin-6-like isoform X2 OS=Phoenix dactylifera OX=42345 GN=LOC103720153 PE=3 SV=1	0	8369
Csativus04462.1	5	1098	A0A2H3ZE42_PHODC8-hydroxygeraniol dehydrogenase-like OS=Phoenix dactylifera OX=42345 GN=LOC103723340	0	8406
Csativus05061.1	5	4455	A0A2H3XTI4_PHODCTranscription elongation factor spt6 OS=Phoenix dactylifera OX=42345 GN=LOC103706290 PE=	0	7916
Csativus05089.1	5	4452	A0A2H3XTI4_PHODCTranscription elongation factor spt6 OS=Phoenix dactylifera OX=42345 GN=LOC103706290 PE=	0	7915
Csativus06645.1	5	633	A0A5P1ECB7_ASPOFS-formylglutathione hydrolase OS=Asparagus officinalis OX=4686 GN=A4U43_C07F15320 PE=3	6,215E-114	8795
Csativus06741.1	5	867	A0A2H3YB39_PHODCS-formylglutathione hydrolase OS=Phoenix dactylifera OX=42345 GN=LOC103711699 PE=3 SV=	7,764E-172	8825

Csativus07451.1	5	3078	A0A2H3XGP6_PHODCFT-interacting protein 1-like OS=Phoenix dactylifera OX=42345 GN=LOC103702639 PE=3 SV=1	0	7463
Csativus07456.1	5	3072	Q9SKA3_ARATHC2 calcium/lipid-binding plant phosphoribosyltransferase family protein OS=Arabidopsis thaliana	0	8124
Csativus09688.1	5	2529	A0A2H3Y4U3_PHODCheat shock 70 kDa protein 14-like OS=Phoenix dactylifera OX=42345 GN=LOC103709743 PE=4 S	0	9046
Csativus10030.1	5	1272	A0A2H3XL10_PHODC26S proteasome regulatory subunit 8 homolog A OS=Phoenix dactylifera OX=42345 GN=LOC1	0	9895
Csativus10051.1	5	1272	A0A2H3XL10_PHODC26S proteasome regulatory subunit 8 homolog A OS=Phoenix dactylifera OX=42345 GN=LOC1	0	9895
Csativus11458.1	2	357	A0A2H3XV93_PHODCuncharacterized protein LOC103706755 OS=Phoenix dactylifera OX=42345 GN=LOC103706755	2,7524E-08	6661
Csativus12760.1	5	753	A0A5P1FD59_ASPOFGrowth-regulating factor OS=Asparagus officinalis OX=4686 GN=A4U43_C03F23250 PE=3 SV=1	4,2736E-64	6484
Csativus12776.1	5	783	A0A2H3XY57_PHODCGrowth-regulating factor OS=Phoenix dactylifera OX=42345 GN=LOC103707535 PE=3 SV=1	7,6045E-60	6673
Csativus14204.1	5	975	A0A2H3X991_PHODCGrowth-regulating factor OS=Phoenix dactylifera OX=42345 GN=LOC103700134 PE=3 SV=1	9,1537E-54	8462
Csativus18561.1	5	5292	MYO7_ARATHMyosin-7 OS=Arabidopsis thaliana OX=3702 GN=XI-A PE=3 SV=1	0	7467
Csativus19757.1	5	2487	A0A5P1FPS0_ASPOFPWWP domain-containing protein OS=Asparagus officinalis OX=4686 GN=A4U43_C02F20920 PI	1,9496E-77	7048
Csativus19809.1	5	2478	A0A5P1FPS0_ASPOFPWWP domain-containing protein OS=Asparagus officinalis OX=4686 GN=A4U43_C02F20920 PI	1,8949E-78	7123
Csativus19813.1	5	2514	A0A5P1FPS0_ASPOFPWWP domain-containing protein OS=Asparagus officinalis OX=4686 GN=A4U43_C02F20920 PI	1,3873E-77	7082
Csativus20438.1	5	1413	A0A2H3Y1U4_PHODCGlycosyltransferase OS=Phoenix dactylifera OX=42345 GN=LOC103708834 PE=3 SV=1	0	7615
Csativus20921.1	5	1410	A0A2H3Y1U4_PHODCGlycosyltransferase OS=Phoenix dactylifera OX=42345 GN=LOC103708834 PE=3 SV=1	0	7639
Csativus21400.1	5	1359	A0A2H3XL10_PHODC26S proteasome regulatory subunit 8 homolog A OS=Phoenix dactylifera OX=42345 GN=LOC1	0	9327
Csativus21738.1	5	2196	A0A2H3XWD4_PHODCzinc finger CCCH domain-containing protein 36-like OS=Phoenix dactylifera OX=42345 GN=L	2,251E-121	601
Csativus21750.1	5	2862	A0A2H3XWD4_PHODCzinc finger CCCH domain-containing protein 36-like OS=Phoenix dactylifera OX=42345 GN=L	2,384E-121	5967
Csativus22992.1	5	1416	A0A2H3YTK7_PHODCSerine hydroxymethyltransferase OS=Phoenix dactylifera OX=42345 GN=LOC103717174 PE=3	0	9397
Csativus23452.1	5	1416	A0A2H3YTK7_PHODCSerine hydroxymethyltransferase OS=Phoenix dactylifera OX=42345 GN=LOC103717174 PE=3	0	9397
Csativus24159.1	5	4560	A0A2H3Z3R8_PHODCmyosin-6-like isoform X1 OS=Phoenix dactylifera OX=42345 GN=LOC103720153 PE=3 SV=1	0	8936
Csativus24510.1	5	4560	A0A2H3ZYC6_PHODCmyosin-6-like isoform X2 OS=Phoenix dactylifera OX=42345 GN=LOC103720153 PE=3 SV=1	0	8932
Csativus25030.1	5	1395	A0A2H3Y1U4_PHODCGlycosyltransferase OS=Phoenix dactylifera OX=42345 GN=LOC103708834 PE=3 SV=1	0	7608
Csativus26178.1	4	567	A0A2H3XN17_PHODCputative invertase inhibitor OS=Phoenix dactylifera OX=42345 GN=LOC103704632 PE=4 SV=1	1,4162E-12	4884
Csativus26193.1	4	564	A0A2H3XN17_PHODCputative invertase inhibitor OS=Phoenix dactylifera OX=42345 GN=LOC103704632 PE=4 SV=1	8,6745E-13	4822
Csativus28492.1	5	768	B9G1H8_ORYSJRRM domain-containing protein OS=Oryza sativa subsp. japonica OX=39947 GN=OsJ_27729 PE=4 SV=	5,3569E-61	9835
Csativus28510.1	5	768	B9G1H8_ORYSJRRM domain-containing protein OS=Oryza sativa subsp. japonica OX=39947 GN=OsJ_27729 PE=4 SV=	1,0373E-59	9623
Csativus28840.1	5	2859	A0A2H3XDJ1_PHODChistidine kinase 5-like OS=Phoenix dactylifera OX=42345 GN=LOC103701735 PE=4 SV=1	0	732
Csativus30678.1	5	1098	A0A2H3ZE42_PHODC8-hydroxygeraniol dehydrogenase-like OS=Phoenix dactylifera OX=42345 GN=LOC103723340	0	8372
Csativus30679.1	5	1098	A0A5P1EP73_ASPOFPKS_ER domain-containing protein OS=Asparagus officinalis OX=4686 GN=A4U43_C06F1410 PE	0	835
Csativus32966.1	5	399	EIL1B_ORYSJProtein ETHYLENE-INSENSITIVE 3-like 1b OS=Oryza sativa subsp. japonica OX=39947 GN=EIL1B PE=2 SV	1,4261E-37	6654
Csativus32978.1	5	1641	A0A0P0VWX9_ORYSJOs03g0324200 protein (Fragment) OS=Oryza sativa subsp. japonica OX=39947 GN=Os03g03242	3,2446E-93	6819
Csativus32980.1	5	1656	A0A2H3XY14_PHODCETHYLENE INSENSITIVE 3-like 1 protein OS=Phoenix dactylifera OX=42345 GN=LOC103707671 F	3,3884E-91	6697
Csativus34819.1	5	996	A0A2H3ZEW0_PHODCGrowth-regulating factor OS=Phoenix dactylifera OX=42345 GN=LOC103723579 PE=3 SV=1	2,752E-121	7297
Csativus35275.1	5	810	A0A2H3XPD2_PHODCGrowth-regulating factor OS=Phoenix dactylifera OX=42345 GN=LOC103704966 PE=3 SV=2	5,6302E-77	7415
Csativus36203.1	4	459	A0A2H3Z576_PHODCputative invertase inhibitor OS=Phoenix dactylifera OX=42345 GN=LOC103720370 PE=4 SV=1	3,2254E-12	5437
Csativus36298.1	5	567	A0A2H3Z576_PHODCputative invertase inhibitor OS=Phoenix dactylifera OX=42345 GN=LOC103720370 PE=4 SV=1	2,8865E-17	5372
Csativus36713.1	5	855	Q65X91_ORYSJOs05g0526200 protein OS=Oryza sativa subsp. japonica OX=39947 GN=Os05g0526200 PE=2 SV=1	3,21E-115	7953
Csativus38217.1	5	1272	A0A2H3Y1M7_PHODC26S proteasome regulatory subunit 8 homolog A-like OS=Phoenix dactylifera OX=42345 GN=I	0	9912
Csativus40085.1	5	579	A0A2H3YT92_PHODCGrowth-regulating factor OS=Phoenix dactylifera OX=42345 GN=LOC103716888 PE=3 SV=1	1,8415E-60	8715
Csativus42590.1	5	1131	A0A2H3ZCT3_PHODCGrowth-regulating factor OS=Phoenix dactylifera OX=42345 GN=LOC103722922 PE=3 SV=1	2,45E-54	5089
Csativus42596.1	5	1131	A0A2H3ZCT3_PHODCGrowth-regulating factor OS=Phoenix dactylifera OX=42345 GN=LOC103722922 PE=3 SV=1	6,589E-42	6216
Csativus42630.1	5	1131	A0A2H3ZCT3_PHODCGrowth-regulating factor OS=Phoenix dactylifera OX=42345 GN=LOC103722922 PE=3 SV=1	2,928E-54	505
Csativus43403.1	5	1002	A0A2H3ZGE7_PHODCGrowth-regulating factor OS=Phoenix dactylifera OX=42345 GN=LOC103724096 PE=3 SV=1	8,577E-137	7383
Csativus43419.1	5	999	A0A5P1F0F3_ASPOFGrowth-regulating factor OS=Asparagus officinalis OX=4686 GN=A4U43_C04F10090 PE=3 SV=1	1,044E-138	7462
Csativus45703.1	5	1893	A0A2H3XY57_PHODCGrowth-regulating factor OS=Phoenix dactylifera OX=42345 GN=LOC103707535 PE=3 SV=1	1,989E-166	633
Csativus45724.1	5	1872	A0A2H3XY57_PHODCGrowth-regulating factor OS=Phoenix dactylifera OX=42345 GN=LOC103707535 PE=3 SV=1	6,579E-170	6224
Csativus46561.1	5	1866	A0A2H3XY57_PHODCGrowth-regulating factor OS=Phoenix dactylifera OX=42345 GN=LOC103707535 PE=3 SV=1	1,555E-162	6386
Csativus48049.1	5	975	A0A2H3X991_PHODCGrowth-regulating factor OS=Phoenix dactylifera OX=42345 GN=LOC103700134 PE=3 SV=1	7,2551E-45	7653
Csativus49065.1	5	1224	A0A5P1FB46_ASPOFAa_trans domain-containing protein OS=Asparagus officinalis OX=4686 GN=A4U43_C03F330 PE	0	7579
Csativus49072.1	5	1224	A0A5P1FB46_ASPOFAa_trans domain-containing protein OS=Asparagus officinalis OX=4686 GN=A4U43_C03F330 PE	0	7581
Csativus50361.1	5	3066	A0A2H3ZB10_PHODCtransportin MOS14 isoform X5 OS=Phoenix dactylifera OX=42345 GN=LOC103722323 PE=4 SV=	0	8237
Csativus50839.1	5	1113	A0A2H3ZDG9_PHODCGrowth-regulating factor OS=Phoenix dactylifera OX=42345 GN=LOC103722922 PE=3 SV=1	2,7717E-42	6774
Csativus50844.1	5	1110	A0A2H3ZDG9_PHODCGrowth-regulating factor OS=Phoenix dactylifera OX=42345 GN=LOC103722922 PE=3 SV=1	2,9588E-41	6744
Csativus51757.1	5	1278	ODPA3_ORYSJPyruvate dehydrogenase E1 component subunit alpha-3, chloroplastic OS=Oryza sativa subsp. japon	0	9036
Csativus52332.1	5	1014	A0A5P1F0F3_ASPOFGrowth-regulating factor OS=Asparagus officinalis OX=4686 GN=A4U43_C04F10090 PE=3 SV=1	4,103E-133	7368
Csativus52413.1	5	612	A0A2H3ZGE7_PHODCGrowth-regulating factor OS=Phoenix dactylifera OX=42345 GN=LOC103724096 PE=3 SV=1	6,5166E-58	8528
Csativus53046.1	5	4746	A0A5P1E6L5_ASPOFHECT-type E3 ubiquitin transferase OS=Asparagus officinalis OX=4686 GN=A4U43_C09F10490 P	0	8552
Csativus53055.1	5	11001	A0A5P1E6L5_ASPOFHECT-type E3 ubiquitin transferase OS=Asparagus officinalis OX=4686 GN=A4U43_C09F10490 P	0	8524
Csativus53147.1	5	2892	A0A2H3ZZG3_PHODCuncharacterized protein LOC103720610 OS=Phoenix dactylifera OX=42345 GN=LOC103720610	0	5719
Csativus53165.1	5	2178	A0A2H3ZZG3_PHODCuncharacterized protein LOC103720610 OS=Phoenix dactylifera OX=42345 GN=LOC103720610	1,068E-127	5657
Csativus53185.1	5	2811	A0A5P1E6L5_ASPOFHECT-type E3 ubiquitin transferase OS=Asparagus officinalis OX=4686 GN=A4U43_C09F10490 P	0	7935
Csativus53583.1	5	2178	A0A2H3ZZG3_PHODCuncharacterized protein LOC103720610 OS=Phoenix dactylifera OX=42345 GN=LOC103720610	6,863E-129	5668
Csativus54338.1	5	735	A0A3Q0IA51_PHODCserine/arginine-rich splicing factor SC35 OS=Phoenix dactylifera OX=42345 GN=LOC103716111	6,9162E-62	9817
Csativus54390.1	5	306	B9G1H8_ORYSJRRM domain-containing protein OS=Oryza sativa subsp. japonica OX=39947 GN=OsJ_27729 PE=4 SV=	1,0812E-58	986
Csativus54727.1	5	2175	A0A2H3YBR3_PHODCphototropin-2 OS=Phoenix dactylifera OX=42345 GN=LOC103711883 PE=4 SV=1	0	8509
Csativus54728.1	5	1443	A0A2H3YBR3_PHODCphototropin-2 OS=Phoenix dactylifera OX=42345 GN=LOC103711883 PE=4 SV=1	0	7305
Csativus56204.1	5	1287	A0A5P1F9A1_ASPOFComponent of oligomeric Golgi complex 3 OS=Asparagus officinalis OX=4686 GN=A4U43_C03F	0	857
Csativus57348.1	5	4407	A0A2H3ZYC6_PHODCmyosin-6-like isoform X2 OS=Phoenix dactylifera OX=42345 GN=LOC103720153 PE=3 SV=1	0	8737
Csativus57557.1	5	957	A0A2H3ZEW0_PHODCGrowth-regulating factor OS=Phoenix dactylifera OX=42345 GN=LOC103723579 PE=3 SV=1	2,063E-120	7021
Csativus57575.1	5	963	A0A2H3ZEW0_PHODCGrowth-regulating factor OS=Phoenix dactylifera OX=42345 GN=LOC103723579 PE=3 SV=1	1,64E-118	7089
Csativus59261.1	5	750	A0A3Q0IA51_PHODCserine/arginine-rich splicing factor SC35 OS=Phoenix dactylifera OX=42345 GN=LOC103716111	1,1668E-60	9817
			**MiR398**		
Csativus02153.1	5	1875	GLO14_ORYSJVery-long-chain aldehyde decarbonylase GL1-4 OS=Oryza sativa subsp. japonica OX=39947 GN=GL1-4	0	8072
Csativus45054.1	5	1515	A0A2H3YA49_PHODCEndoglucanase OS=Phoenix dactylifera OX=42345 GN=LOC103711412 PE=3 SV=1	0	9069
Csativus46759.1	5	2961	A0A5P1EVJ0_ASPOFExocyst subunit Exo70 family protein OS=Asparagus officinalis OX=4686 GN=A4U43_C05F28260	0	8498
