# Supplementary material for: Characterization of Exosome-like Nanoparticles from Saffron Tepals and Their Immunostimulatory Activity
Source: Biology (Basel). 2025 Feb 18;14(2):215. doi: 10.3390/biology14020215 (PMC11851917; doi:10.3390/biology14020215)
Supplement: Supplementary file 1 [file biology-14-00215-s001.zip › Table S5.pdf]

Table S5. Relation of proteins identified in saffron flowers exosomes, using the Arabidopsis proteome.

Accession	Description	Score	Coverage	# Proteins	# Unique Peptides	# Peptides	# PSMs
P29197	Chaperonin CPN60, mitochondrial OS=Arabidopsis thaliana GN=CPN60 PE=1 SV=2 - [CH60A_ARATH]	700,99	26,17	3	17	17	20
Q9SZN1	V-type proton ATPase subunit B2 OS=Arabidopsis thaliana OX=3702 GN=VHA-B2 PE=1 SV=1 - [VATB2_ARATH]	676,00	25,46	3	11	11	21
Q0WNJ6	Clathrin heavy chain 1 OS=Arabidopsis thaliana OX=3702 GN=CHC1 PE=1 SV=1 - [CLAH1_ARATH]	562,23	10,56	2	16	16	17
Q9SU58	ATPase 4, plasma membrane-type OS=Arabidopsis thaliana OX=3702 GN=AHA4 PE=2 SV=2 - [PMA4_ARATH]	434,10	9,27	3	1	10	13
P19456	ATPase 2, plasma membrane-type OS=Arabidopsis thaliana OX=3702 GN=AHA2 PE=1 SV=2 - [PMA2_ARATH]	412,65	11,92	5	5	12	16
Q9LK36	Adenosylhomocysteinase 2 OS=Arabidopsis thaliana OX=3702 GN=SAHH2 PE=2 SV=1 - [SAHH2_ARATH]	389,90	22,27	2	11	11	14
Q9LV11	ATPase 11, plasma membrane-type OS=Arabidopsis thaliana OX=3702 GN=AHA11 PE=1 SV=1 - [PMA11_ARATH]	349,82	10,04	3	1	10	12
O23654	V-type proton ATPase catalytic subunit A OS=Arabidopsis thaliana OX=3702 GN=VHA-A PE=1 SV=1 - [VATA_ARATH]	343,08	15,25	1	8	8	12
Q9M2A0	ATPase 8, plasma membrane-type OS=Arabidopsis thaliana OX=3702 GN=AHA8 PE=3 SV=1 - [PMA8_ARATH]	342,84	8,33	3	1	8	10
P83483	ATP synthase subunit beta-1, mitochondrial OS=Arabidopsis thaliana OX=3702 GN=At5g08670 PE=1 SV=1 - [ATPBM_ARATH]	336,54	15,65	4	6	6	8
P25819	Catalase-2 OS=Arabidopsis thaliana OX=3702 GN=CAT2 PE=1 SV=3 - [CATA2_ARATH]	325,20	8,74	1	2	4	13
Q9MAH0	Phosphoenolpyruvate carboxylase 1 OS=Arabidopsis thaliana OX=3702 GN=PPC1 PE=1 SV=1 - [CAPP1_ARATH]	305,87	11,07	1	4	9	10
Q9C7X7	Heat shock 70 kDa protein 18 OS=Arabidopsis thaliana OX=3702 GN=HSP70-18 PE=2 SV=1 - [HSP7N_ARATH]	297,64	15,40	1	1	7	8
Q42556	ATPase 9, plasma membrane-type OS=Arabidopsis thaliana OX=3702 GN=AHA9 PE=2 SV=2 - [PMA9_ARATH]	293,26	9,22	2	1	7	9
B9DGT7	Tubulin alpha-2 chain OS=Arabidopsis thaliana OX=3702 GN=TUBA2 PE=2 SV=2 - [TBA2_ARATH]	290,00	18,22	2	2	8	10
P22954	Heat shock 70 kDa protein 2 OS=Arabidopsis thaliana OX=3702 GN=HSP70-2 PE=1 SV=2 - [HS702_ARATH]	284,41	15,62	3	1	8	9
Q8LD27	Proteasome subunit beta type-6 OS=Arabidopsis thaliana OX=3702 GN=PBA1 PE=1 SV=2 - [PSB6_ARATH]	277,18	17,60	1	4	4	6
P12411	Tubulin beta-1 chain OS=Arabidopsis thaliana OX=3702 GN=TUBB1 PE=2 SV=1 - [TBB1_ARATH]	275,26	10,07	5	1	5	8
Q9SCN8	Cell division control protein 48 homolog D OS=Arabidopsis thaliana OX=3702 GN=CDC48D PE=1 SV=1 - [CD48D_ARATH]	270,80	17,55	3	11	11	12
O65719	Heat shock 70 kDa protein 3 OS=Arabidopsis thaliana OX=3702 GN=HSP70-3 PE=1 SV=1 - [HSP7C_ARATH]	265,25	12,63	1	1	6	7
P93306	NADH dehydrogenase [ubiquinone] iron-sulfur protein 2 OS=Arabidopsis thaliana OX=3702 GN=NAD7 PE=1 SV=2 - [NDUS2_ARATH]	261,89	21,83	1	7	7	10
P92963	Ras-related protein RABB1c OS=Arabidopsis thaliana OX=3702 GN=RABB1C PE=1 SV=1 - [RAB1C_ARATH]	259,57	31,75	3	5	5	8
P31414	Pyrophosphate-energized vacuolar membrane proton pump 1 OS=Arabidopsis thaliana OX=3702 GN=AVP1 PE=1 SV=1 - [AVP1_ARATH]	252,97	4,81	1	4	4	5
P31167	ADP,ATP carrier protein 1, mitochondrial OS=Arabidopsis thaliana OX=3702 GN=AAC1 PE=1 SV=2 - [ADT1_ARATH]	245,55	15,22	3	5	5	8
Q9S9N1	Heat shock 70 kDa protein 5 OS=Arabidopsis thaliana OX=3702 GN=HSP70-5 PE=2 SV=1 - [HSP7E_ARATH]	244,40	9,44	1	1	4	6
Q95748	NADH dehydrogenase [ubiquinone] iron-sulfur protein 3 OS=Arabidopsis thaliana OX=3702 GN=NAD9 PE=1 SV=2 - [NDUS3_ARATH]	240,66	31,05	1	6	6	6
O50008	5-methyltetrahydropteroyltriglutamate--homocysteine methyltransferase 1 OS=Arabidopsis thaliana OX=3702 GN=MS1 PE=1 SV=1 - [METE1_ARATH]	238,06	5,88	3	4	4	5
P28186	Ras-related protein RABE1c OS=Arabidopsis thaliana OX=3702 GN=RABE1C PE=1 SV=1 - [RAE1C_ARATH]	236,44	27,78	14	4	6	9

P53494	Actin-4 OS=Arabidopsis thaliana GN=ACT4 PE=1 SV=1 - [ACT4_ARATH]	234,86	18,30	8	6	6	7
Q9FGX1	ATP-citrate synthase beta chain protein 2 OS=Arabidopsis thaliana OX=3702 GN=ACLB-2 PE=1 SV=1 - [ACLB2_ARATH]	225,49	9,70	2	5	5	6
Q84VW9	Phosphoenolpyruvate carboxylase 3 OS=Arabidopsis thaliana OX=3702 GN=PPC3 PE=1 SV=2 - [CAPP3_ARATH]	224,49	7,64	1	1	6	7
Q9LZH9	60S ribosomal protein L7a-2 OS=Arabidopsis thaliana OX=3702 GN=RPL7AB PE=1 SV=1 - [RL7A2_ARATH]	222,83	13,67	2	4	4	9
P29514	Tubulin beta-6 chain OS=Arabidopsis thaliana OX=3702 GN=TUBB6 PE=2 SV=1 - [TBB6_ARATH]	221,21	10,02	6	1	5	7
Q43128	ATPase 10, plasma membrane-type OS=Arabidopsis thaliana OX=3702 GN=AHA10 PE=2 SV=2 - [PMA10_ARATH]	218,01	4,75	1	1	5	6
P46286	60S ribosomal protein L8-1 OS=Arabidopsis thaliana OX=3702 GN=RPL8A PE=1 SV=2 - [RL81_ARATH]	217,97	15,89	2	1	3	5
Q9LF98	Fructose-bisphosphate aldolase 8, cytosolic OS=Arabidopsis thaliana OX=3702 GN=FBA8 PE=1 SV=1 - [ALFC8_ARATH]	206,88	13,97	4	6	6	6
Q56WH1	Tubulin alpha-3 chain OS=Arabidopsis thaliana OX=3702 GN=TUBA3 PE=1 SV=2 - [TBA3_ARATH]	191,82	13,11	1	1	7	8
P42794	60S ribosomal protein L11-2 OS=Arabidopsis thaliana OX=3702 GN=RPL11B PE=2 SV=2 - [RL112_ARATH]	186,97	26,37	2	5	5	7
Q39043	Heat shock 70 kDa protein BIP2 OS=Arabidopsis thaliana OX=3702 GN=BIP2 PE=1 SV=2 - [BIP2_ARATH]	183,89	7,93	2	3	4	4
Q9SN35	Ras-related protein RABA1d OS=Arabidopsis thaliana OX=3702 GN=RABA1D PE=1 SV=1 - [RAA1D_ARATH]	180,90	28,50	22	2	6	6
P59259	Histone H4 OS=Arabidopsis thaliana OX=3702 GN=At1g07660 PE=1 SV=2 - [H4_ARATH]	180,85	51,46	1	6	6	7
P17094	60S ribosomal protein L3-1 OS=Arabidopsis thaliana OX=3702 GN=ARP1 PE=1 SV=5 - [RL31_ARATH]	180,78	13,88	2	5	5	8
Q9XIW1	Probable xyloglucan endotransglucosylase/hydrolase protein 5 OS=Arabidopsis thaliana OX=3702 GN=XTH5 PE=2 SV=1 - [XTH5_ARATH]	173,73	13,99	4	4	4	6
Q9FJA6	40S ribosomal protein S3-3 OS=Arabidopsis thaliana OX=3702 GN=RPS3C PE=1 SV=1 - [RS33_ARATH]	171,20	16,94	3	4	4	6
Q8H159	Polyubiquitin 10 OS=Arabidopsis thaliana OX=3702 GN=UBQ10 PE=2 SV=2 - [UBQ10_ARATH]	169,62	40,70	14	4	4	8
P30184	Leucine aminopeptidase 1 OS=Arabidopsis thaliana OX=3702 GN=LAP1 PE=1 SV=1 - [AMPL1_ARATH]	164,90	6,73	3	3	3	4
P0DH99	Elongation factor 1-alpha 1 OS=Arabidopsis thaliana GN=A1 PE=1 SV=1 - [EF1A1_ARATH]	163,79	6,68	1	3	3	5
P42792	Cytochrome b OS=Arabidopsis thaliana OX=3702 GN=MT-CYB PE=1 SV=2 - [CYB_ARATH]	160,55	8,14	1	3	3	4
Q8L8Y0	40S ribosomal protein S2-1 OS=Arabidopsis thaliana OX=3702 GN=RPS2A PE=2 SV=2 - [RS21_ARATH]	159,80	11,62	4	4	4	5
P42791	60S ribosomal protein L18-2 OS=Arabidopsis thaliana OX=3702 GN=RPL18B PE=1 SV=2 - [RL182_ARATH]	157,30	13,37	2	2	2	3
Q3B724	Callose synthase 5 OS=Arabidopsis thaliana OX=3702 GN=CALS5 PE=1 SV=1 - [CALS5_ARATH]	156,42	2,55	6	2	4	6
P92549	ATP synthase subunit alpha, mitochondrial OS=Arabidopsis thaliana OX=3702 GN=ATPA PE=1 SV=2 - [ATPAM_ARATH]	153,24	9,66	1	5	5	6
O24616	Proteasome subunit alpha type-7-B OS=Arabidopsis thaliana OX=3702 GN=PAD2 PE=1 SV=2 - [PSA7B_ARATH]	152,83	13,20	2	3	3	4
P0CAN7	V-type proton ATPase subunit E3 OS=Arabidopsis thaliana OX=3702 GN=VHA-E3 PE=2 SV=1 - [VATE3_ARATH]	150,09	11,81	3	3	3	4
Q96300	14-3-3-like protein GF14 nu OS=Arabidopsis thaliana OX=3702 GN=GRF7 PE=1 SV=1 - [14337_ARATH]	149,19	12,45	8	4	4	4
Q9FPJ4	Ras-related protein RABD2b OS=Arabidopsis thaliana OX=3702 GN=RABD2B PE=1 SV=1 - [RAD2B_ARATH]	148,67	14,85	12	1	3	5
Q42064	60S ribosomal protein L8-3 OS=Arabidopsis thaliana OX=3702 GN=RPL8C PE=2 SV=2 - [RL83_ARATH]	142,24	15,89	2	1	3	4
Q9SIL6	Prohibitin-6, mitochondrial OS=Arabidopsis thaliana OX=3702 GN=PHB6 PE=1 SV=1 - [PHB6_ARATH]	141,84	5,24	3	1	1	2
Q42093	ABC transporter C family member 2 OS=Arabidopsis thaliana OX=3702 GN=ABCC2 PE=1 SV=2 - [AB2C_ARATH]	135,51	1,91	1	1	3	4
O04331	Prohibitin-3, mitochondrial OS=Arabidopsis thaliana OX=3702 GN=PHB3 PE=1 SV=1 - [PHB3_ARATH]	134,22	9,39	2	2	2	3
O49299	Probable phosphoglucomutase, cytoplasmic 1 OS=Arabidopsis thaliana OX=3702 GN=At1g23190 PE=2 SV=2 - [PGMC1_ARATH]	133,70	4,97	2	3	3	3

Q1PEX3	Ras-related protein RABA1h OS=Arabidopsis thaliana OX=3702 GN=RABA1H PE=2 SV=1 - [RAA1H_ARATH]	133,55	23,85	16	1	5	5
P11139	Tubulin alpha-1 chain OS=Arabidopsis thaliana OX=3702 GN=TUBA1 PE=2 SV=1 - [TBA1_ARATH]	132,43	9,78	1	1	5	6
O23712	Proteasome subunit alpha type-1-B OS=Arabidopsis thaliana OX=3702 GN=PAF2 PE=1 SV=2 - [PSA1B_ARATH]	132,35	13,72	2	3	3	3
O81147	Proteasome subunit alpha type-6-B OS=Arabidopsis thaliana OX=3702 GN=PAA2 PE=1 SV=1 - [PSA6B_ARATH]	127,41	10,98	2	3	3	4
Q96528	Catalase-1 OS=Arabidopsis thaliana OX=3702 GN=CAT1 PE=1 SV=3 - [CATA1_ARATH]	126,83	5,89	1	1	3	4
Q9C9C5	60S ribosomal protein L6-3 OS=Arabidopsis thaliana OX=3702 GN=RPL6C PE=2 SV=1 - [RL63_ARATH]	125,27	6,44	3	1	1	3
Q8LAH8	Nucleoside diphosphate kinase IV, chloroplastic/mitochondrial OS=Arabidopsis thaliana OX=3702 GN=NDK4 PE=1 SV=2 - [NDK4_ARATH]	121,81	13,08	2	4	4	4
P60040	60S ribosomal protein L7-2 OS=Arabidopsis thaliana OX=3702 GN=RPL7B PE=1 SV=1 - [RL72_ARATH]	120,85	10,74	3	3	3	5
P55737	Heat shock protein 90-2 OS=Arabidopsis thaliana OX=3702 GN=HSP90-2 PE=1 SV=1 - [HS902_ARATH]	120,48	4,72	3	3	3	3
Q96254	Guanosine nucleotide diphosphate dissociation inhibitor 1 OS=Arabidopsis thaliana OX=3702 GN=GDI1 PE=1 SV=1 - [GDI1_ARATH]	118,62	4,72	1	3	3	4
F4JJE5	Putative proteasome subunit alpha type-4-B OS=Arabidopsis thaliana OX=3702 GN=PAC2 PE=5 SV=1 - [PSA4B_ARATH]	116,59	13,94	2	3	3	4
Q9LF33	UDP-glucose 6-dehydrogenase 3 OS=Arabidopsis thaliana OX=3702 GN=UGD3 PE=1 SV=1 - [UGDH3_ARATH]	115,27	6,46	3	3	3	3
Q9ZU52	Fructose-bisphosphate aldolase 3, chloroplastic OS=Arabidopsis thaliana OX=3702 GN=FBA3 PE=1 SV=1 - [ALFP3_ARATH]	115,23	5,63	3	2	2	2
Q9M9P3	UTP--glucose-1-phosphate uridylyltransferase 2 OS=Arabidopsis thaliana OX=3702 GN=UGP2 PE=1 SV=1 - [UGPA2_ARATH]	113,51	4,90	2	2	2	3
O81149	Proteasome subunit alpha type-5-A OS=Arabidopsis thaliana OX=3702 GN=PAE1 PE=1 SV=1 - [PSA5A_ARATH]	111,29	17,30	2	4	4	4
P93819	Malate dehydrogenase 1, cytoplasmic OS=Arabidopsis thaliana OX=3702 GN=MDH1 PE=1 SV=2 - [MDHC1_ARATH]	110,44	9,94	3	3	3	4
Q8LPJ5	Isocitrate dehydrogenase [NADP], chloroplastic/mitochondrial OS=Arabidopsis thaliana OX=3702 GN=At5g14590 PE=1 SV=1 - [ICDHP_ARATH]	108,86	4,33	1	1	2	3
Q9SN95	UDP-glucuronic acid decarboxylase 5 OS=Arabidopsis thaliana OX=3702 GN=UXS5 PE=2 SV=1 - [UXS5_ARATH]	108,61	9,68	6	3	3	3
O23254	Serine hydroxymethyltransferase 4 OS=Arabidopsis thaliana OX=3702 GN=SHM4 PE=1 SV=1 - [GLYC4_ARATH]	108,02	5,31	1	2	2	2
O22666	UDP-arabinopyranose mutase 3 OS=Arabidopsis thaliana OX=3702 GN=RGP3 PE=1 SV=2 - [RGP3_ARATH]	107,22	8,29	3	1	3	4
Q9FLF0	40S ribosomal protein S9-2 OS=Arabidopsis thaliana OX=3702 GN=RPS9C PE=1 SV=1 - [RS92_ARATH]	105,30	12,69	2	4	4	6
P17562	S-adenosylmethionine synthase 2 OS=Arabidopsis thaliana OX=3702 GN=SAM2 PE=1 SV=1 - [METK2_ARATH]	102,36	11,96	4	3	3	4
O82647	Pyruvate decarboxylase 1 OS=Arabidopsis thaliana OX=3702 GN=PDC1 PE=2 SV=1 - [PDC1_ARATH]	102,36	5,11	4	3	3	4
Q9SID0	Probable fructokinase-1 OS=Arabidopsis thaliana OX=3702 GN=At2g31390 PE=2 SV=1 - [SCRK1_ARATH]	101,76	7,69	5	2	2	2
P51418	60S ribosomal protein L18a-2 OS=Arabidopsis thaliana OX=3702 GN=RPL18AB PE=1 SV=2 - [R18A2_ARATH]	101,73	15,17	2	1	4	5
Q8H1G6	PTI1-like tyrosine-protein kinase 1 OS=Arabidopsis thaliana OX=3702 GN=PTI11 PE=1 SV=1 - [PTI11_ARATH]	99,76	5,54	5	1	2	2
P29402	Calnexin homolog 1 OS=Arabidopsis thaliana OX=3702 GN=CNX1 PE=1 SV=1 - [CALX1_ARATH]	98,84	5,66	1	2	2	3
Q9SJB3	ATPase 5, plasma membrane-type OS=Arabidopsis thaliana OX=3702 GN=AHA5 PE=3 SV=3 - [PMA5_ARATH]	98,70	4,53	2	1	4	4
P34788	40S ribosomal protein S18 OS=Arabidopsis thaliana OX=3702 GN=RPS18A PE=1 SV=1 - [RS18_ARATH]	98,48	11,18	1	2	2	2
Q9SRT9	UDP-arabinopyranose mutase 1 OS=Arabidopsis thaliana OX=3702 GN=RGP1 PE=1 SV=1 - [RGP1_ARATH]	97,97	8,68	3	1	3	3
Q9SFU6	Callose synthase 9 OS=Arabidopsis thaliana OX=3702 GN=CALS9 PE=2 SV=2 - [CALS9_ARATH]	96,95	1,59	2	2	2	2
P42742	Proteasome subunit beta type-1 OS=Arabidopsis thaliana OX=3702 GN=PBF1 PE=1 SV=2 - [PSB1_ARATH]	96,15	6,28	1	1	1	2

Q9XEC4	Aspartic proteinase A3 OS=Arabidopsis thaliana OX=3702 GN=APA3 PE=2 SV=1 - [APA3_ARATH]	96,12	1,97	1	1	1	2
Q93VT9	60S ribosomal protein L10-1 OS=Arabidopsis thaliana OX=3702 GN=RPL10A PE=1 SV=1 - [RL101_ARATH]	95,52	9,09	3	2	2	2
Q9SRL5	Ferritin-2, chloroplastic OS=Arabidopsis thaliana OX=3702 GN=FER2 PE=2 SV=1 - [FRI2_ARATH]	95,39	3,95	1	2	2	4
Q9FM19	Hypersensitive-induced response protein 1 OS=Arabidopsis thaliana OX=3702 GN=HIR1 PE=1 SV=1 - [HIR1_ARATH]	94,74	10,84	2	2	3	3
Q94A40	Coatomer subunit alpha-1 OS=Arabidopsis thaliana OX=3702 GN=At1g62020 PE=2 SV=2 - [COPA1_ARATH]	94,44	3,04	2	4	4	4
Q9CAR7	Hypersensitive-induced response protein 2 OS=Arabidopsis thaliana OX=3702 GN=HIR2 PE=1 SV=1 - [HIR2_ARATH]	94,41	6,29	1	1	2	2
Q9SK66	NADH dehydrogenase [ubiquinone] 1 alpha subcomplex subunit 9, mitochondrial OS=Arabidopsis thaliana OX=3702 GN=At2g20360 PE=1 SV=2 - [NDUA9_ARATH]	93,10	4,98	1	2	2	3
Q9LSQ5	NAD(P)H dehydrogenase (quinone) FQR1 OS=Arabidopsis thaliana OX=3702 GN=FQR1 PE=1 SV=1 - [FQR1_ARATH]	92,98	13,24	2	2	2	2
Q9FNN5	NADH dehydrogenase [ubiquinone] flavoprotein 1, mitochondrial OS=Arabidopsis thaliana OX=3702 GN=At5g08530 PE=1 SV=1 - [NDUV1_ARATH]	91,69	10,49	1	4	4	5
Q9LUD4	60S ribosomal protein L18a-3 OS=Arabidopsis thaliana OX=3702 GN=RPL18AC PE=2 SV=1 - [R18A3_ARATH]	91,54	14,61	2	1	4	5
Q9ZU25	Probable mitochondrial-processing peptidase subunit alpha-1, mitochondrial OS=Arabidopsis thaliana OX=3702 GN=MPPalpha1 PE=1 SV=1 - [MPPA1_ARATH]	91,27	3,58	2	2	2	2
Q9C5M0	Mitochondrial dicarboxylate/tricarboxylate transporter DTC OS=Arabidopsis thaliana OX=3702 GN=DTC PE=1 SV=1 - [DTC_ARATH]	91,10	4,03	1	1	1	2
Q9C820	Ras-related protein RABG3d OS=Arabidopsis thaliana OX=3702 GN=RABG3D PE=2 SV=1 - [RAG3D_ARATH]	90,65	15,53	6	3	3	3
Q93Y35	26S proteasome non-ATPase regulatory subunit 6 homolog OS=Arabidopsis thaliana OX=3702 GN=RPN7 PE=1 SV=1 - [PSMD6_ARATH]	89,93	6,20	1	2	2	2
P51427	40S ribosomal protein S5-2 OS=Arabidopsis thaliana OX=3702 GN=RPS5B PE=1 SV=2 - [RS52_ARATH]	89,23	17,87	2	4	4	5
F4JFN3	Heat shock protein 90-6, mitochondrial OS=Arabidopsis thaliana OX=3702 GN=HSP90-6 PE=1 SV=1 - [HS906_ARATH]	89,21	1,75	2	2	2	2
P36397	ADP-ribosylation factor 1 OS=Arabidopsis thaliana OX=3702 GN=ARF1 PE=1 SV=2 - [ARF1_ARATH]	88,36	13,26	2	2	2	2
P24704	Superoxide dismutase [Cu-Zn] 1 OS=Arabidopsis thaliana OX=3702 GN=CSD1 PE=1 SV=2 - [SODC1_ARATH]	87,55	8,55	2	1	1	2
Q8W4S4	V-type proton ATPase subunit a3 OS=Arabidopsis thaliana OX=3702 GN=VHA-a3 PE=1 SV=1 - [VHAA3_ARATH]	86,97	3,29	2	3	3	3
P42825	Chaperone protein dnaJ 2 OS=Arabidopsis thaliana OX=3702 GN=ATJ2 PE=1 SV=2 - [DNAJ2_ARATH]	86,70	2,86	2	1	1	1
Q9SRQ7	Non-specific phospholipase C4 OS=Arabidopsis thaliana OX=3702 GN=NPC4 PE=1 SV=1 - [NPC4_ARATH]	85,83	1,86	1	1	1	2
P61837	Aquaporin PIP1-1 OS=Arabidopsis thaliana OX=3702 GN=PIP1-1 PE=1 SV=1 - [PIP11_ARATH]	83,91	3,50	5	1	1	3
Q0WML0	ABC transporter B family member 27 OS=Arabidopsis thaliana OX=3702 GN=ABCB27 PE=1 SV=1 - [AB27B_ARATH]	81,96	1,86	1	1	1	1
Q9C8G9	ABC transporter C family member 1 OS=Arabidopsis thaliana OX=3702 GN=ABCC1 PE=1 SV=1 - [AB1C_ARATH]	81,78	1,48	3	1	3	3
Q70DU8	Aldehyde dehydrogenase family 3 member H1 OS=Arabidopsis thaliana OX=3702 GN=ALDH3H1 PE=1 SV=2 - [AL3H1_ARATH]	81,70	4,34	1	2	2	3
P25858	Glyceraldehyde-3-phosphate dehydrogenase GAPC1, cytosolic OS=Arabidopsis thaliana OX=3702 GN=GAPC1 PE=1 SV=2 - [G3PC1_ARATH]	81,64	6,80	2	2	2	2
Q9FFC0	Histone H2B.10 OS=Arabidopsis thaliana OX=3702 GN=At5g22880 PE=1 SV=3 - [H2B10_ARATH]	79,99	20,69	10	3	3	3
P42036	40S ribosomal protein S14-3 OS=Arabidopsis thaliana OX=3702 GN=RPS14C PE=2 SV=2 - [RS143_ARATH]	79,77	16,00	3	2	2	2
Q6XJG8	26S proteasome non-ATPase regulatory subunit 2 homolog B OS=Arabidopsis thaliana OX=3702 GN=RPN1B PE=1 SV=1 - [PSD2B_ARATH]	78,98	2,81	2	2	2	3
Q42290	Probable mitochondrial-processing peptidase subunit beta, mitochondrial OS=Arabidopsis thaliana OX=3702 GN=MPPbeta PE=1 SV=2 - [MPPB_ARATH]	78,09	5,46	1	3	3	3

P49227	60S ribosomal protein L5-2 OS=Arabidopsis thaliana OX=3702 GN=RPL5B PE=2 SV=3 - [RL52_ARATH]	77,92	7,97	2	3	3	3
Q9LH76	Trifunctional UDP-glucose 4,6-dehydratase/UDP-4-keto-6-deoxy-D-glucose 3,5-epimerase/UDP-4-keto-L-rhamnose-reductase RHM3 OS=Arabidopsis thaliana OX=3702 GN=RHM3 PE=2 SV=1 - [RHM3_ARATH]	77,89	3,46	3	1	2	2
P45725	Phenylalanine ammonia-lyase 3 OS=Arabidopsis thaliana OX=3702 GN=PAL3 PE=1 SV=3 - [PAL3_ARATH]	77,50	2,16	4	1	1	1
Q9FKC0	60S ribosomal protein L13a-4 OS=Arabidopsis thaliana OX=3702 GN=RPL13AD PE=2 SV=1 - [R13A4_ARATH]	77,39	12,62	4	2	2	2
Q944R1	Probable pectate lyase 15 OS=Arabidopsis thaliana OX=3702 GN=At4g13710 PE=2 SV=1 - [PLY15_ARATH]	76,19	4,04	4	2	2	2
F4JVN6	Tripeptidyl-peptidase 2 OS=Arabidopsis thaliana OX=3702 GN=TPP2 PE=1 SV=1 - [TPPII_ARATH]	75,73	0,87	1	1	1	1
Q9M2V7	ABC transporter G family member 16 OS=Arabidopsis thaliana OX=3702 GN=ABCG16 PE=2 SV=2 - [AB16G_ARATH]	75,18	3,67	5	3	3	3
O04204	60S acidic ribosomal protein P0-1 OS=Arabidopsis thaliana OX=3702 GN=RPP0A PE=1 SV=1 - [RLA01_ARATH]	74,52	7,57	3	2	2	2
P41127	60S ribosomal protein L13-1 OS=Arabidopsis thaliana OX=3702 GN=RPL13B PE=1 SV=1 - [RL131_ARATH]	73,81	5,83	1	1	1	2
Q9SE96	GEM-like protein 1 OS=Arabidopsis thaliana OX=3702 GN=FIP1 PE=1 SV=1 - [GEML1_ARATH]	73,54	4,63	1	1	1	1
P42798	40S ribosomal protein S15a-1 OS=Arabidopsis thaliana OX=3702 GN=RPS15AA PE=2 SV=2 - [R15A1_ARATH]	73,33	10,77	2	1	1	2
Q9FIF3	40S ribosomal protein S8-2 OS=Arabidopsis thaliana OX=3702 GN=RPS8B PE=2 SV=1 - [RS82_ARATH]	72,59	7,14	1	1	1	1
P41376	Eukaryotic initiation factor 4A-1 OS=Arabidopsis thaliana OX=3702 GN=EIF4A1 PE=1 SV=1 - [IF4A1_ARATH]	72,37	10,19	4	4	4	4
P59226	Histone H3.1 OS=Arabidopsis thaliana OX=3702 GN=HTR2 PE=1 SV=2 - [H31_ARATH]	70,14	11,76	6	2	2	2
Q9SGE0	UDP-D-apiose/UDP-D-xylose synthase 2 OS=Arabidopsis thaliana OX=3702 GN=AXS2 PE=2 SV=1 - [AXS2_ARATH]	70,05	6,17	2	2	2	2
Q8L828	Coatomer subunit beta'-3 OS=Arabidopsis thaliana OX=3702 GN=At3g15980 PE=2 SV=1 - [COB23_ARATH]	69,98	2,53	3	2	2	2
Q9SYP2	Pyrophosphate--fructose 6-phosphate 1-phosphotransferase subunit alpha 1 OS=Arabidopsis thaliana OX=3702 GN=PFP-ALPHA1 PE=1 SV=1 - [PFPA1_ARATH]	69,37	5,05	2	2	2	2
P51413	60S ribosomal protein L17-2 OS=Arabidopsis thaliana OX=3702 GN=RPL17B PE=2 SV=2 - [RL172_ARATH]	69,18	5,14	2	1	1	2
Q9SS48	Glycerol-3-phosphate dehydrogenase SDP6, mitochondrial OS=Arabidopsis thaliana OX=3702 GN=SDP6 PE=1 SV=1 - [SDP6_ARATH]	68,90	2,54	1	2	2	2
Q9SF40	60S ribosomal protein L4-1 OS=Arabidopsis thaliana OX=3702 GN=RPL4A PE=1 SV=1 - [RL4A_ARATH]	68,01	3,69	2	2	2	2
P58766	Phospholipase D alpha 3 OS=Arabidopsis thaliana OX=3702 GN=PLDALPHA3 PE=2 SV=1 - [PLDA3_ARATH]	67,75	2,68	2	2	2	2
O23715	Proteasome subunit alpha type-3 OS=Arabidopsis thaliana OX=3702 GN=PAG1 PE=1 SV=2 - [PSA3_ARATH]	67,35	7,23	1	2	2	2
Q9MAT0	26S proteasome non-ATPase regulatory subunit 1 homolog B OS=Arabidopsis thaliana OX=3702 GN=RPN2B PE=1 SV=1 - [PSD1B_ARATH]	67,18	1,20	2	1	1	1
P49040	Sucrose synthase 1 OS=Arabidopsis thaliana OX=3702 GN=SUS1 PE=1 SV=3 - [SUS1_ARATH]	66,76	1,73	2	1	1	2
Q9LT08	26S proteasome non-ATPase regulatory subunit 14 homolog OS=Arabidopsis thaliana OX=3702 GN=RPN11 PE=1 SV=1 - [PSDE_ARATH]	66,46	4,22	1	1	1	1
Q9S7L9	Cytochrome c oxidase subunit 6b-1 OS=Arabidopsis thaliana OX=3702 GN=COX6B-1 PE=1 SV=1 - [CX6B1_ARATH]	66,27	8,90	3	2	2	2
O23708	Proteasome subunit alpha type-2-A OS=Arabidopsis thaliana OX=3702 GN=PAB1 PE=1 SV=1 - [PSA2A_ARATH]	66,24	14,47	2	2	2	2
Q9LK23	Glucose-6-phosphate 1-dehydrogenase 5, cytoplasmic OS=Arabidopsis thaliana OX=3702 GN=G6PD5 PE=1 SV=1 - [G6PD5_ARATH]	66,20	3,68	2	2	2	2
Q7DLS1	Proteasome subunit beta type-7-B OS=Arabidopsis thaliana OX=3702 GN=PBB2 PE=1 SV=2 - [PSB7B_ARATH]	66,16	8,39	2	2	2	2
Q944K2	Dolichyl-diphosphooligosaccharide--protein glycosyltransferase 48 kDa subunit OS=Arabidopsis thaliana OX=3702 GN=OST48 PE=2 SV=1 - [OST48_ARATH]	65,99	2,75	1	1	1	1

Q8LD46	60S ribosomal protein L23a-1 OS=Arabidopsis thaliana OX=3702 GN=RPL23AA PE=2 SV=2 - [R23A1_ARATH]	64,99	8,44	2	1	1	1
Q9LD28	Histone H2A.6 OS=Arabidopsis thaliana OX=3702 GN=RAT5 PE=1 SV=1 - [H2A6_ARATH]	63,95	6,92	11	1	1	1
Q8LEE7	Ubiquinol oxidase 3, mitochondrial OS=Arabidopsis thaliana OX=3702 GN=AOX3 PE=1 SV=2 - [AOX3_ARATH]	63,63	3,14	5	1	1	1
Q9LZD3	Exocyst complex component EXO70A1 OS=Arabidopsis thaliana OX=3702 GN=EXO70A1 PE=1 SV=1 - [E70A1_ARATH]	62,63	1,88	1	1	1	1
Q9SJ11	Ras-related protein RABG2 OS=Arabidopsis thaliana OX=3702 GN=RABG2 PE=2 SV=2 - [RABG2_ARATH]	62,32	7,08	1	1	1	1
Q9C4Z6	Receptor for activated C kinase 1B OS=Arabidopsis thaliana OX=3702 GN=RACK1B PE=1 SV=1 - [GPLPB_ARATH]	60,81	4,91	3	2	2	2
Q9LQ04	Bifunctional dTDP-4-dehydrorhamnose 3,5-epimerase/dTDP-4-dehydrorhamnose reductase OS=Arabidopsis thaliana OX=3702 GN=NRS/ER PE=1 SV=1 - [RMLCD_ARATH]	60,71	6,31	1	1	2	2
O65572	Carotenoid 9,10(9',10')-cleavage dioxygenase 1 OS=Arabidopsis thaliana OX=3702 GN=CCD1 PE=1 SV=2 - [CCD1_ARATH]	60,68	3,35	1	1	1	1
Q8RXU5	60S ribosomal protein L37a-2 OS=Arabidopsis thaliana OX=3702 GN=RPL37AC PE=3 SV=1 - [R37A2_ARATH]	60,50	26,09	2	2	2	2
Q9SWG0	Isovaleryl-CoA dehydrogenase, mitochondrial OS=Arabidopsis thaliana OX=3702 GN=IVD PE=1 SV=2 - [IVD_ARATH]	60,00	2,93	1	1	1	1
Q940S0	Transmembrane 9 superfamily member 2 OS=Arabidopsis thaliana OX=3702 GN=TMN2 PE=2 SV=1 - [TMN2_ARATH]	59,99	2,03	3	1	1	1
Q8RWL2	Calcium-dependent protein kinase 29 OS=Arabidopsis thaliana OX=3702 GN=CPK29 PE=2 SV=2 - [CDPKT_ARATH]	59,73	2,25	1	1	1	1
Q9FGI6	NADH dehydrogenase [ubiquinone] iron-sulfur protein 1, mitochondrial OS=Arabidopsis thaliana OX=3702 GN=EMB1467 PE=1 SV=2 - [NDUS1_ARATH]	59,13	1,74	1	1	1	1
Q1PE89	Probable serine/threonine-protein kinase PBL24 OS=Arabidopsis thaliana OX=3702 GN=PBL24 PE=2 SV=1 - [PBL24_ARATH]	58,91	4,11	48	1	2	3
P48348	14-3-3-like protein GF14 kappa OS=Arabidopsis thaliana OX=3702 GN=GRF8 PE=1 SV=2 - [14338_ARATH]	58,85	4,84	2	2	2	2
Q04613	ATP synthase protein MI25 OS=Arabidopsis thaliana OX=3702 GN=AtMg00640 PE=1 SV=2 - [MI25_ARATH]	58,43	4,17	1	1	1	1
O22925	Vacuolar-sorting receptor 2 OS=Arabidopsis thaliana OX=3702 GN=VSR2 PE=2 SV=1 - [VSR2_ARATH]	58,25	2,24	1	1	1	1
Q94II3	Probable methyltransferase PMT21 OS=Arabidopsis thaliana OX=3702 GN=ERD3 PE=2 SV=1 - [PMTL_ARATH]	58,17	1,67	1	1	1	1
Q9LFF9	Soluble inorganic pyrophosphatase 4 OS=Arabidopsis thaliana OX=3702 GN=PPA4 PE=1 SV=1 - [IPYR4_ARATH]	57,97	5,56	1	1	1	1
O82616	Probable fructokinase-5 OS=Arabidopsis thaliana OX=3702 GN=At4g10260 PE=2 SV=1 - [SCRK5_ARATH]	57,96	2,78	1	1	1	1
P43292	Serine/threonine-protein kinase SRK2G OS=Arabidopsis thaliana OX=3702 GN=SRK2G PE=1 SV=2 - [SRK2G_ARATH]	57,95	4,53	4	1	1	1
Q8H118	Probable methyltransferase PMT1 OS=Arabidopsis thaliana OX=3702 GN=At3g23300 PE=2 SV=2 - [PMT1_ARATH]	57,78	3,60	4	2	2	2
Q9SLK2	ALA-interacting subunit 3 OS=Arabidopsis thaliana OX=3702 GN=ALIS3 PE=1 SV=1 - [ALIS3_ARATH]	57,72	3,44	2	1	1	1
Q1WIQ6	NADP-dependent glyceraldehyde-3-phosphate dehydrogenase OS=Arabidopsis thaliana OX=3702 GN=ALDH11A3 PE=1 SV=2 - [GAPN_ARATH]	57,50	3,63	1	2	2	2
Q9LXT9	Callose synthase 3 OS=Arabidopsis thaliana OX=3702 GN=CALS3 PE=3 SV=3 - [CALS3_ARATH]	57,07	1,38	5	1	3	3
O64817	Casein kinase II subunit alpha-3 OS=Arabidopsis thaliana OX=3702 GN=CKA3 PE=1 SV=1 - [CSK23_ARATH]	56,67	3,60	3	1	1	1
Q75W54	Mannosylglycoprotein endo-beta-mannosidase OS=Arabidopsis thaliana OX=3702 GN=EBM PE=1 SV=3 - [EBM_ARATH]	56,09	1,06	1	1	1	1
Q38919	Rac-like GTP-binding protein ARAC4 OS=Arabidopsis thaliana OX=3702 GN=ARAC4 PE=1 SV=1 - [RAC4_ARATH]	56,08	5,64	11	1	1	1
Q9M1Q9	ABC transporter B family member 21 OS=Arabidopsis thaliana OX=3702 GN=ABCB21 PE=1 SV=2 - [AB21B_ARATH]	55,92	1,47	9	2	2	2
O04019	26S proteasome regulatory subunit 6A homolog B OS=Arabidopsis thaliana OX=3702 GN=RPT5B PE=1 SV=3 - [PS6AB_ARATH]	55,30	6,62	7	2	2	2
P29388	NADH-ubiquinone oxidoreductase chain 5 OS=Arabidopsis thaliana OX=3702 GN=ND5 PE=1 SV=3 - [NU5M_ARATH]	55,19	1,49	1	1	1	1
O81845	Mitochondrial uncoupling protein 1 OS=Arabidopsis thaliana OX=3702 GN=PUMP1 PE=1 SV=1 - [PUMP1_ARATH]	55,02	5,23	1	1	1	1

Q9SLK0	Peroxisomal isocitrate dehydrogenase [NADP] OS=Arabidopsis thaliana OX=3702 GN=ICDH PE=1 SV=1 - [ICDHX_ARATH]	54,81	7,45	2	2	3	3
P31582	Ras-related protein RABF2a OS=Arabidopsis thaliana OX=3702 GN=RABF2A PE=1 SV=1 - [RAF2A_ARATH]	54,44	5,50	3	1	1	1
P93285	Cytochrome c oxidase subunit 2 OS=Arabidopsis thaliana OX=3702 GN=COX2 PE=1 SV=2 - [COX2_ARATH]	54,43	2,69	1	1	1	1
P31265	Translationally-controlled tumor protein 1 OS=Arabidopsis thaliana OX=3702 GN=TCTP1 PE=1 SV=2 - [TCTP1_ARATH]	53,84	5,36	1	1	1	1
Q93ZE8	Stromal cell-derived factor 2-like protein OS=Arabidopsis thaliana OX=3702 GN=SDF2 PE=1 SV=1 - [SDF2_ARATH]	53,32	5,50	1	1	1	1
Q9SD76	Alpha-glucan phosphorylase 2, cytosolic OS=Arabidopsis thaliana OX=3702 GN=PHS2 PE=1 SV=1 - [PHS2_ARATH]	53,13	2,97	1	1	2	2
Q9C554	Expansin-A1 OS=Arabidopsis thaliana OX=3702 GN=EXPA1 PE=2 SV=1 - [EXPA1_ARATH]	52,83	3,60	1	1	1	1
Q7FB56	Putative ABC transporter C family member 15 OS=Arabidopsis thaliana OX=3702 GN=ABCC15 PE=5 SV=2 - [AB15C_ARATH]	52,36	0,85	3	1	1	1
Q8RWN9	Dihydrolipoyllysine-residue acetyltransferase component 2 of pyruvate dehydrogenase complex, mitochondrial OS=Arabidopsis thaliana OX=3702 GN=At3g13930 PE=1 SV=2 - [ODP22_ARATH]	52,27	3,90	1	2	2	2
Q3EDL4	Probable serine/threonine-protein kinase At1g01540 OS=Arabidopsis thaliana OX=3702 GN=At1g01540 PE=1 SV=2 - [Y1154_ARATH]	52,19	3,39	17	2	2	2
Q9FNA9	Phospholipid:diacylglycerol acyltransferase 1 OS=Arabidopsis thaliana OX=3702 GN=PDAT1 PE=2 SV=1 - [PDAT1_ARATH]	51,93	1,34	1	1	1	1
P92558	NADH-ubiquinone oxidoreductase chain 1 OS=Arabidopsis thaliana OX=3702 GN=ND1 PE=1 SV=4 - [NU1M_ARATH]	51,67	6,77	1	2	2	2
Q9LIK0	Plastidial pyruvate kinase 1, chloroplastic OS=Arabidopsis thaliana OX=3702 GN=PKP1 PE=1 SV=1 - [PKP1_ARATH]	51,62	1,85	1	1	1	1
Q9LQR8	26S proteasome non-ATPase regulatory subunit 3 homolog B OS=Arabidopsis thaliana OX=3702 GN=RPN3B PE=1 SV=2 - [PSD3B_ARATH]	51,35	1,85	2	1	1	1
Q9ASR1	Elongation factor 2 OS=Arabidopsis thaliana OX=3702 GN=LOS1 PE=1 SV=1 - [EF2_ARATH]	51,30	2,73	1	2	2	2
Q9FNP8	40S ribosomal protein S19-3 OS=Arabidopsis thaliana OX=3702 GN=RPS19C PE=2 SV=1 - [RS193_ARATH]	51,06	9,09	3	1	1	2
F4I2X9	Probable alkaline/neutral invertase D OS=Arabidopsis thaliana OX=3702 GN=INVD PE=2 SV=1 - [INVD_ARATH]	51,02	2,25	1	1	1	1
O04084	Serine carboxypeptidase-like 31 OS=Arabidopsis thaliana OX=3702 GN=SCPL31 PE=2 SV=2 - [SCP31_ARATH]	51,00	2,24	1	1	1	1
Q9FHM7	Hypersensitive-induced response protein 4 OS=Arabidopsis thaliana OX=3702 GN=HIR4 PE=1 SV=1 - [HIR4_ARATH]	50,93	2,74	1	1	1	1
Q9SYT0	Annexin D1 OS=Arabidopsis thaliana OX=3702 GN=ANN1 PE=1 SV=1 - [ANXD1_ARATH]	50,40	2,84	1	1	1	1
Q94AI6	Exocyst complex component SEC6 OS=Arabidopsis thaliana OX=3702 GN=SEC6 PE=1 SV=1 - [SEC6_ARATH]	50,07	1,46	1	1	1	1
Q38946	Glutamate dehydrogenase 2 OS=Arabidopsis thaliana OX=3702 GN=GDH2 PE=1 SV=1 - [DHE2_ARATH]	49,37	2,92	2	1	1	1
Q9M3D6	ABC transporter G family member 19 OS=Arabidopsis thaliana GN=ABCG19 PE=1 SV=1 - [AB19G_ARATH]	49,32	1,52	1	1	1	1
Q9SF16	T-complex protein 1 subunit eta OS=Arabidopsis thaliana OX=3702 GN=CCT7 PE=1 SV=1 - [TCPH_ARATH]	48,78	2,15	1	1	1	1
Q9LIP2	Proteasome subunit beta type-5-B OS=Arabidopsis thaliana OX=3702 GN=PBE2 PE=1 SV=1 - [PSB5B_ARATH]	48,54	4,76	2	1	1	1
Q9STF0	Receptor like protein kinase S.3 OS=Arabidopsis thaliana OX=3702 GN=LECRKS3 PE=3 SV=1 - [LRKS3_ARATH]	48,39	2,97	2	1	1	1
Q42560	Aconitate hydratase 1 OS=Arabidopsis thaliana OX=3702 GN=ACO1 PE=1 SV=2 - [ACO1_ARATH]	48,38	2,45	2	2	2	2
O04378	Syntaxin-23 OS=Arabidopsis thaliana OX=3702 GN=SYP23 PE=1 SV=1 - [SYP23_ARATH]	48,35	6,27	2	2	2	2
O80695	Protein DETOXIFICATION 37 OS=Arabidopsis thaliana OX=3702 GN=DTX37 PE=2 SV=1 - [DTX37_ARATH]	48,32	2,20	3	1	1	1
Q94AZ4	Probable calcium-binding protein CML13 OS=Arabidopsis thaliana OX=3702 GN=CML13 PE=2 SV=1 - [CML13_ARATH]	48,01	8,11	1	1	1	1
Q38866	Expansin-A2 OS=Arabidopsis thaliana OX=3702 GN=EXPA2 PE=2 SV=2 - [EXPA2_ARATH]	47,46	3,53	1	1	1	1
Q8VXZ7	Alpha-galactosidase 3 OS=Arabidopsis thaliana OX=3702 GN=AGAL3 PE=1 SV=1 - [AGAL3_ARATH]	46,74	2,29	1	1	1	1

O22769	NADH dehydrogenase [ubiquinone] flavoprotein 2, mitochondrial OS=Arabidopsis thaliana OX=3702 GN=At4g02580 PE=1 SV=3 - [NDUV2_ARATH]	46,73	3,14	1	1	1	1
Q94B38	Glucose-6-phosphate/phosphate translocator 2, chloroplastic OS=Arabidopsis thaliana OX=3702 GN=GPT2 PE=2 SV=2 - [GPT2_ARATH]	46,67	3,09	2	1	1	1
Q84K16	AP-1 complex subunit gamma-1 OS=Arabidopsis thaliana OX=3702 GN=GAMMA-ADR PE=1 SV=1 - [AP1G1_ARATH]	46,50	1,03	1	1	1	1
O64903	Nucleoside diphosphate kinase II, chloroplastic OS=Arabidopsis thaliana OX=3702 GN=NDPK2 PE=1 SV=2 - [NDK2_ARATH]	46,50	3,90	1	1	1	1
P51430	40S ribosomal protein S6-2 OS=Arabidopsis thaliana OX=3702 GN=RPS6B PE=1 SV=3 - [RS62_ARATH]	46,50	6,02	2	1	1	1
P54887	Delta-1-pyrroline-5-carboxylate synthase A OS=Arabidopsis thaliana OX=3702 GN=P5CSA PE=1 SV=1 - [P5CS1_ARATH]	46,21	1,53	2	1	1	1
P41916	GTP-binding nuclear protein Ran-1 OS=Arabidopsis thaliana OX=3702 GN=RAN1 PE=1 SV=1 - [RAN1_ARATH]	45,55	4,98	3	1	1	1
Q9SV68	Chloroplast envelope quinone oxidoreductase homolog OS=Arabidopsis thaliana OX=3702 GN=CEQORH PE=1 SV=1 - [QORH_ARATH]	45,45	3,34	1	1	1	1
Q93Z92	E3 ubiquitin-protein ligase At4g11680 OS=Arabidopsis thaliana OX=3702 GN=At4g11680 PE=2 SV=1 - [RING4_ARATH]	45,35	2,56	1	1	1	1
O23461	L-arabinokinase OS=Arabidopsis thaliana OX=3702 GN=ARA1 PE=1 SV=1 - [ARAK_ARATH]	45,08	0,96	1	1	1	1
Q9LUT0	Receptor-like cytoplasmic kinase 1 OS=Arabidopsis thaliana OX=3702 GN=CARK1 PE=1 SV=1 - [CARK1_ARATH]	44,58	5,77	2	1	2	2
Q42340	40S ribosomal protein S16-3 OS=Arabidopsis thaliana OX=3702 GN=RPS16C PE=2 SV=1 - [RS163_ARATH]	44,36	5,48	3	1	1	1
Q9XI91	Eukaryotic translation initiation factor 5A-1 OS=Arabidopsis thaliana OX=3702 GN=ELF5A-1 PE=1 SV=1 - [IF5A1_ARATH]	44,31	6,33	1	1	1	1
Q9M088	Glucan endo-1,3-beta-glucosidase 5 OS=Arabidopsis thaliana OX=3702 GN=At4g31140 PE=2 SV=1 - [E135_ARATH]	43,94	2,27	1	1	1	1
Q9FLJ8	Probable receptor-like protein kinase At5g61350 OS=Arabidopsis thaliana OX=3702 GN=At5g61350 PE=2 SV=1 - [Y5613_ARATH]	43,77	0,95	5	1	1	1
F4JLM5	Isoleucine--tRNA ligase, cytoplasmic OS=Arabidopsis thaliana OX=3702 GN=At4g10320 PE=2 SV=1 - [SYIC_ARATH]	43,67	0,84	1	1	1	1
P42697	Phragmoplastin DRP1A OS=Arabidopsis thaliana OX=3702 GN=DRP1A PE=1 SV=3 - [DRP1A_ARATH]	43,34	4,59	1	3	3	3
Q94A18	ABC transporter G family member 29 OS=Arabidopsis thaliana OX=3702 GN=ABCG29 PE=2 SV=2 - [AB29G_ARATH]	42,65	0,71	4	1	1	1
Q9LIB2	Alpha-glucan phosphorylase 1 OS=Arabidopsis thaliana OX=3702 GN=PHS1 PE=1 SV=1 - [PHS1_ARATH]	42,30	2,60	1	1	2	2
O65660	PLAT domain-containing protein 1 OS=Arabidopsis thaliana OX=3702 GN=PLAT1 PE=1 SV=1 - [PLAT1_ARATH]	41,75	4,97	1	1	1	1
P59223	40S ribosomal protein S13-1 OS=Arabidopsis thaliana OX=3702 GN=RPS13A PE=2 SV=1 - [RS131_ARATH]	41,68	7,95	2	1	1	1
Q9FKS5	Cytochrome c1 2, heme protein, mitochondrial OS=Arabidopsis thaliana OX=3702 GN=CYC1-2 PE=1 SV=1 - [CYC1B_ARATH]	41,66	5,86	2	1	1	1
Q6R3K4	Probable metal-nicotianamine transporter YSL8 OS=Arabidopsis thaliana OX=3702 GN=YSL8 PE=1 SV=2 - [YSL8_ARATH]	41,55	1,38	1	1	1	1
Q9LJI5	V-type proton ATPase subunit d1 OS=Arabidopsis thaliana OX=3702 GN=VHA-d1 PE=1 SV=1 - [VA0D1_ARATH]	41,44	2,28	1	1	1	1
O23714	Proteasome subunit beta type-2-A OS=Arabidopsis thaliana OX=3702 GN=PBD1 PE=1 SV=1 - [PSB2A_ARATH]	41,37	7,35	1	1	1	1
F4IZ82	ADP-ribosylation factor-like protein 8d OS=Arabidopsis thaliana OX=3702 GN=ARL8D PE=3 SV=1 - [ARL8D_ARATH]	41,36	5,11	3	1	1	1
Q8S528	Aldehyde dehydrogenase family 2 member B7, mitochondrial OS=Arabidopsis thaliana OX=3702 GN=ALDH2B7 PE=1 SV=2 - [AL2B7_ARATH]	41,33	1,50	2	1	1	1
Q9SZR1	Calcium-transporting ATPase 10, plasma membrane-type OS=Arabidopsis thaliana OX=3702 GN=ACA10 PE=1 SV=2 - [ACA10_ARATH]	41,32	1,12	1	1	1	1
Q9FT52	ATP synthase subunit d, mitochondrial OS=Arabidopsis thaliana OX=3702 GN=At3g52300 PE=1 SV=3 - [ATP5H_ARATH]	41,16	5,36	1	1	1	1
Q84WG0	Protein NRT1/ PTR FAMILY 8.4 OS=Arabidopsis thaliana OX=3702 GN=NPF8.4 PE=2 SV=2 - [PTR26_ARATH]	41,16	1,28	1	1	1	1
Q9LNJ3	Aspartyl protease family protein 2 OS=Arabidopsis thaliana OX=3702 GN=APF2 PE=2 SV=1 - [APF2_ARATH]	41,04	1,65	1	1	1	1

Q9SKT7	External alternative NAD(P)H-ubiquinone oxidoreductase B4, mitochondrial OS=Arabidopsis thaliana OX=3702 GN=NDB4 PE=1 SV=1 - [NDB4_ARATH]	40,82	1,55	1	1	1	1
Q9T0G2	Cytochrome c-2 OS=Arabidopsis thaliana OX=3702 GN=CYTC-2 PE=1 SV=1 - [CYC2_ARATH]	40,73	7,14	2	1	1	1
P25696	Bifunctional enolase 2/transcriptional activator OS=Arabidopsis thaliana OX=3702 GN=ENO2 PE=1 SV=1 - [ENO2_ARATH]	40,52	4,95	1	1	1	1
Q93ZM7	Chaperonin CPN60-like 2, mitochondrial OS=Arabidopsis thaliana OX=3702 GN=At3g13860 PE=1 SV=2 - [CH60C_ARATH]	40,22	2,27	1	1	1	1
O81153	Proteasome subunit beta type-3-B OS=Arabidopsis thaliana OX=3702 GN=PBC2 PE=1 SV=1 - [PSB3B_ARATH]	40,07	4,41	2	1	1	1
Q9SV20	Coatomer subunit beta-2 OS=Arabidopsis thaliana OX=3702 GN=At4g31490 PE=2 SV=2 - [COPB2_ARATH]	39,98	1,05	2	1	1	1
P39207	Nucleoside diphosphate kinase 1 OS=Arabidopsis thaliana OX=3702 GN=NDK1 PE=1 SV=1 - [NDK1_ARATH]	39,88	10,74	1	1	1	1
Q08682	40S ribosomal protein Sa-1 OS=Arabidopsis thaliana OX=3702 GN=RPSaA PE=1 SV=3 - [RSSA1_ARATH]	39,83	3,69	1	1	1	2
Q8LF48	3-ketoacyl-CoA thiolase 1, peroxisomal OS=Arabidopsis thaliana OX=3702 GN=KAT1 PE=1 SV=2 - [THIK1_ARATH]	39,58	2,03	2	1	1	1
P35131	Ubiquitin-conjugating enzyme E2 8 OS=Arabidopsis thaliana OX=3702 GN=UBC8 PE=1 SV=1 - [UBC8_ARATH]	39,50	7,43	5	1	1	1
P51420	60S ribosomal protein L31-3 OS=Arabidopsis thaliana OX=3702 GN=RPL31C PE=3 SV=2 - [RL313_ARATH]	39,43	6,72	3	1	1	1
Q9S795	Aminoaldehyde dehydrogenase ALDH10A8, chloroplastic OS=Arabidopsis thaliana OX=3702 GN=ALDH10A8 PE=1 SV=1 - [BADH1_ARATH]	39,20	2,20	1	1	1	1
Q94CE5	Gamma-aminobutyrate transaminase POP2, mitochondrial OS=Arabidopsis thaliana OX=3702 GN=POP2 PE=1 SV=1 - [GATP_ARATH]	38,91	4,17	1	2	2	2
O81742	Beta-adaptin-like protein C OS=Arabidopsis thaliana OX=3702 GN=BETAC-AD PE=1 SV=2 - [APBLC_ARATH]	38,61	1,12	2	1	1	1
O49377	Vesicle-associated membrane protein 711 OS=Arabidopsis thaliana OX=3702 GN=VAMP711 PE=1 SV=2 - [VA711_ARATH]	38,54	7,76	1	1	1	1
F4JWS8	Cytochrome b-c1 complex subunit 7-2, mitochondrial OS=Arabidopsis thaliana OX=3702 GN=QCR7-2 PE=1 SV=1 - [QCR72_ARATH]	38,47	9,02	2	1	1	1
B5X574	Phosphoenolpyruvate carboxykinase (ATP) 2 OS=Arabidopsis thaliana OX=3702 GN=PCK2 PE=1 SV=1 - [PCKA2_ARATH]	38,22	2,24	2	1	1	1
Q9LSH2	Glutamate decarboxylase 5 OS=Arabidopsis thaliana OX=3702 GN=GAD5 PE=2 SV=1 - [DCE5_ARATH]	38,02	4,25	2	2	2	2
O22718	ATP-citrate synthase alpha chain protein 2 OS=Arabidopsis thaliana OX=3702 GN=ACLA-2 PE=2 SV=1 - [ACLA2_ARATH]	37,84	2,36	3	1	1	1
Q9ZQ31	Serine/threonine-protein kinase STY13 OS=Arabidopsis thaliana OX=3702 GN=STY13 PE=1 SV=2 - [STY13_ARATH]	37,38	1,95	2	1	1	1
Q9LKA3	Malate dehydrogenase 2, mitochondrial OS=Arabidopsis thaliana GN=At3g15020 PE=1 SV=1 - [MDHM2_ARATH]	37,31	3,52	2	1	1	1
Q9LVD9	Protein DETOXIFICATION 40 OS=Arabidopsis thaliana OX=3702 GN=DTX40 PE=1 SV=1 - [DTX40_ARATH]	36,67	2,17	1	1	1	1
Q9XGM1	V-type proton ATPase subunit D OS=Arabidopsis thaliana OX=3702 GN=VHA-D PE=1 SV=2 - [VATD_ARATH]	36,64	3,45	1	1	1	1
Q9FKU8	Berberine bridge enzyme-like 26 OS=Arabidopsis thaliana OX=3702 GN=At5g44400 PE=2 SV=1 - [BBE26_ARATH]	36,64	1,49	1	1	1	1
Q9M111	Sucrose synthase 3 OS=Arabidopsis thaliana OX=3702 GN=SUS3 PE=1 SV=1 - [SUS3_ARATH]	36,60	1,48	1	1	1	1
Q8H173	40S ribosomal protein Sa-2 OS=Arabidopsis thaliana OX=3702 GN=RPSaB PE=1 SV=2 - [RSSA2_ARATH]	36,19	6,07	1	1	1	1
Q56ZN6	Pyrophosphate-energized membrane proton pump 2 OS=Arabidopsis thaliana OX=3702 GN=AVPL1 PE=1 SV=2 - [AVP2_ARATH]	36,08	1,75	1	1	1	1
Q42605	Bifunctional UDP-glucose 4-epimerase and UDP-xylose 4-epimerase 1 OS=Arabidopsis thaliana OX=3702 GN=UGE1 PE=1 SV=2 - [UGE1_ARATH]	35,86	2,28	2	1	1	1
Q682B4	Putative beta-glucosidase 6 OS=Arabidopsis thaliana OX=3702 GN=BGLU6 PE=5 SV=1 - [BGL06_ARATH]	35,72	1,85	24	1	1	1
Q9SDS7	V-type proton ATPase subunit C OS=Arabidopsis thaliana OX=3702 GN=VHA-C PE=1 SV=1 - [VATC_ARATH]	35,68	1,87	1	1	1	1
Q9SF85	Adenosine kinase 1 OS=Arabidopsis thaliana OX=3702 GN=ADK1 PE=1 SV=1 - [ADK1_ARATH]	35,62	3,78	2	1	1	1

Q9LX65	V-type proton ATPase subunit H OS=Arabidopsis thaliana OX=3702 GN=VHA-H PE=1 SV=1 - [VATH_ARATH]	35,59	1,59	1	1	1	1
Q94F20	Protein DUF642 L-GALACTONO-1,4-LACTONE-RESPONSIVE GENE 2 OS=Arabidopsis thaliana OX=3702 GN=DGR2 PE=2 SV=1 - [DGR2_ARATH]	35,57	2,17	1	1	1	1
O78310	Superoxide dismutase [Cu-Zn] 2, chloroplastic OS=Arabidopsis thaliana OX=3702 GN=CSD2 PE=1 SV=2 - [SODC2_ARATH]	35,45	6,94	1	1	1	1
Q9M9K1	Probable 2,3-bisphosphoglycerate-independent phosphoglycerate mutase 2 OS=Arabidopsis thaliana OX=3702 GN=PGM2 PE=1 SV=1 - [PMG2_ARATH]	35,42	3,39	1	2	2	2
Q9LTB2	Methionine S-methyltransferase OS=Arabidopsis thaliana OX=3702 GN=MMT1 PE=1 SV=1 - [MMT1_ARATH]	35,29	0,84	1	1	1	1
P32746	Dihydroorotate dehydrogenase (quinone), mitochondrial OS=Arabidopsis thaliana OX=3702 GN=PYRD PE=1 SV=2 - [PYRD_ARATH]	35,29	1,74	1	1	1	1
Q4V3B3	Beta-glucosidase 28 OS=Arabidopsis thaliana OX=3702 GN=BGLU28 PE=2 SV=1 - [BGL28_ARATH]	35,26	1,20	2	1	1	1
Q9SAY1	Sulfate transporter 1.1 OS=Arabidopsis thaliana OX=3702 GN=SULTR1;1 PE=1 SV=2 - [SUT11_ARATH]	35,23	3,54	3	2	2	2
Q9FJD0	Vacuolar protein sorting-associated protein 26A OS=Arabidopsis thaliana OX=3702 GN=VPS26A PE=2 SV=1 - [VP26A_ARATH]	34,93	3,64	2	1	1	1
P46637	Arginase 1, mitochondrial OS=Arabidopsis thaliana OX=3702 GN=ARGAH1 PE=1 SV=1 - [ARGI1_ARATH]	34,71	2,63	1	1	1	1
Q8H103	Glucose-6-phosphate isomerase 1, chloroplastic OS=Arabidopsis thaliana OX=3702 GN=PGI1 PE=1 SV=1 - [G6PIP_ARATH]	34,61	1,14	1	1	1	1
O80844	Probable methyltransferase PMT16 OS=Arabidopsis thaliana OX=3702 GN=At2g45750 PE=3 SV=1 - [PMTG_ARATH]	34,41	2,06	4	1	1	1
Q94A97	Ubiquitin-conjugating enzyme E2 35 OS=Arabidopsis thaliana OX=3702 GN=UBC35 PE=1 SV=1 - [UBC35_ARATH]	34,33	4,58	2	1	1	1
F4JL11	Importin subunit alpha-2 OS=Arabidopsis thaliana OX=3702 GN=IMPA2 PE=1 SV=1 - [IMPA2_ARATH]	34,33	1,31	1	1	1	1
P48491	Triosephosphate isomerase, cytosolic OS=Arabidopsis thaliana OX=3702 GN=CTIMC PE=1 SV=2 - [TPIS_ARATH]	34,21	3,15	2	1	1	1
O48788	Probable inactive receptor kinase At2g26730 OS=Arabidopsis thaliana OX=3702 GN=At2g26730 PE=1 SV=1 - [Y2267_ARATH]	34,17	1,82	1	1	1	1
P93033	Fumarate hydratase 1, mitochondrial OS=Arabidopsis thaliana OX=3702 GN=FUM1 PE=1 SV=2 - [FUM1_ARATH]	34,04	1,63	2	1	1	1
Q9SUN6	Subtilisin-like protease SBT2.2 OS=Arabidopsis thaliana OX=3702 GN=SBT2.2 PE=3 SV=1 - [SBT22_ARATH]	33,84	1,99	1	1	1	1
Q8GXW5	Glutamine synthetase cytosolic isozyme 1-5 OS=Arabidopsis thaliana OX=3702 GN=GLN1-5 PE=1 SV=1 - [GLN15_ARATH]	33,71	2,27	3	1	1	1
P52577	Isoflavone reductase homolog P3 OS=Arabidopsis thaliana OX=3702 GN=At1g75280 PE=2 SV=1 - [IFRH_ARATH]	33,69	2,58	1	1	1	1
Q9LFB2	Auxin transporter-like protein 1 OS=Arabidopsis thaliana OX=3702 GN=LAX1 PE=2 SV=1 - [LAX1_ARATH]	33,31	2,05	1	1	1	1
O80448	Pyridoxal 5'-phosphate synthase subunit PDX1.1 OS=Arabidopsis thaliana OX=3702 GN=PDX11 PE=1 SV=1 - [PDX11_ARATH]	32,85	4,85	1	1	1	1
Q9ZQY6	Serine/threonine protein phosphatase 2A 55 kDa regulatory subunit B' delta isoform OS=Arabidopsis thaliana OX=3702 GN=B'DELTA PE=1 SV=1 - [2A5D_ARATH]	32,56	2,31	6	1	1	1
P92533	NADH-ubiquinone oxidoreductase chain 3 OS=Arabidopsis thaliana OX=3702 GN=ND3 PE=1 SV=2 - [NU3M_ARATH]	32,50	12,61	1	1	1	1
Q94JT5	Cyclase-like protein 2 OS=Arabidopsis thaliana OX=3702 GN=CYCLASE2 PE=2 SV=1 - [CYL2_ARATH]	32,50	4,78	1	1	1	1
Q9LIR4	Dihydroxy-acid dehydratase, chloroplastic OS=Arabidopsis thaliana OX=3702 GN=DHAD PE=1 SV=1 - [ILVD_ARATH]	32,15	1,48	1	1	1	1
Q9LP45	26S proteasome non-ATPase regulatory subunit 11 homolog OS=Arabidopsis thaliana OX=3702 GN=RPN6 PE=1 SV=1 - [PSD11_ARATH]	31,97	2,63	1	1	1	1
Q8L866	Amine oxidase [copper-containing] zeta, peroxisomal OS=Arabidopsis thaliana OX=3702 GN=CuAOzeta PE=1 SV=1 - [CUAOZ_ARATH]	31,96	1,03	1	1	1	1
P51422	60S ribosomal protein L35a-4 OS=Arabidopsis thaliana OX=3702 GN=RPL35AD PE=3 SV=2 - [R35A4_ARATH]	31,03	10,81	4	1	1	1
P0CZ23	Acyl-coenzyme A oxidase 3, peroxisomal OS=Arabidopsis thaliana OX=3702 GN=ACX3 PE=1 SV=1 - [ACOX3_ARATH]	30,78	1,04	2	1	1	1
Q93VH9	40S ribosomal protein S4-1 OS=Arabidopsis thaliana OX=3702 GN=RPS4A PE=2 SV=1 - [RS41_ARATH]	30,72	3,07	3	1	1	1

Q9CA83	NADP-dependent malic enzyme 4, chloroplastic OS=Arabidopsis thaliana OX=3702 GN=NADP-ME4 PE=1 SV=1 - [MAOP4_ARATH]	30,44	1,39	1	1	1	1
Q9SCV8	Beta-galactosidase 4 OS=Arabidopsis thaliana OX=3702 GN=BGAL4 PE=1 SV=1 - [BGAL4_ARATH]	30,31	1,52	6	1	1	1
Q93VR3	GDP-mannose 3,5-epimerase OS=Arabidopsis thaliana OX=3702 GN=At5g28840 PE=1 SV=1 - [GME_ARATH]	30,18	1,86	1	1	1	1
Q9SUS0	Endoglucanase 20 OS=Arabidopsis thaliana OX=3702 GN=At4g23560 PE=2 SV=1 - [GUN20_ARATH]	29,61	1,67	1	1	1	1
Q93WF1	Probable pectate lyase 20 OS=Arabidopsis thaliana OX=3702 GN=At5g48900 PE=2 SV=1 - [PLY20_ARATH]	29,28	2,16	2	1	1	1
Q9M063	Putative GEM-like protein 3 OS=Arabidopsis thaliana OX=3702 GN=At4g40100 PE=3 SV=2 - [GEML3_ARATH]	28,48	3,77	1	1	1	1
Q84WU2	Ubiquitin C-terminal hydrolase 13 OS=Arabidopsis thaliana OX=3702 GN=UBP13 PE=1 SV=1 - [UBP13_ARATH]	27,67	1,08	2	1	1	1
Q9FJD4	Importin subunit beta-1 OS=Arabidopsis thaliana OX=3702 GN=KPNB1 PE=1 SV=1 - [IMB1_ARATH]	27,62	1,03	1	1	1	1
Q9M2Z8	Mitochondrial phosphate carrier protein 2, mitochondrial OS=Arabidopsis thaliana OX=3702 GN=MPT2 PE=2 SV=1 - [MPCP2_ARATH]	26,91	3,58	1	1	1	1
Q9FFD2	Probable UDP-arabinopyranose mutase 5 OS=Arabidopsis thaliana OX=3702 GN=RGP5 PE=1 SV=1 - [RGP5_ARATH]	26,68	2,30	1	1	1	1
Q0WQQ1	Probable ADP-ribosylation factor GTPase-activating protein AGD15 OS=Arabidopsis thaliana OX=3702 GN=AGD15 PE=2 SV=1 - [AGD15_ARATH]	26,40	3,02	4	1	1	1
Q8LPJ3	Probable alpha-mannosidase At5g13980 OS=Arabidopsis thaliana OX=3702 GN=At5g13980 PE=2 SV=1 - [MANA2_ARATH]	26,36	0,88	1	1	1	1
Q93V61	Phospholipase A(1) LCAT3 OS=Arabidopsis thaliana OX=3702 GN=LCAT3 PE=1 SV=1 - [LCAT3_ARATH]	26,34	2,68	1	1	1	1
Q9FJX2	60S ribosomal protein L26-2 OS=Arabidopsis thaliana OX=3702 GN=RPL26B PE=2 SV=1 - [RL262_ARATH]	25,67	5,48	1	1	1	1
P92514	Cytochrome c oxidase subunit 3 OS=Arabidopsis thaliana OX=3702 GN=COX3 PE=2 SV=2 - [COX3_ARATH]	25,48	3,02	1	1	1	1
Q9FWA3	6-phosphogluconate dehydrogenase, decarboxylating 2 OS=Arabidopsis thaliana OX=3702 GN=PGD2 PE=1 SV=1 - [6PGD2_ARATH]	25,46	1,85	3	1	1	1
B9DFI7	Probable methyltransferase PMT2 OS=Arabidopsis thaliana OX=3702 GN=At1g26850 PE=2 SV=2 - [PMT2_ARATH]	25,29	2,11	1	1	1	1
P49693	60S ribosomal protein L19-3 OS=Arabidopsis thaliana OX=3702 GN=RPL19C PE=2 SV=3 - [RL193_ARATH]	25,23	4,33	2	1	1	1
Q9FHN6	Monocopper oxidase-like protein SKS2 OS=Arabidopsis thaliana OX=3702 GN=SKS2 PE=2 SV=1 - [SKS2_ARATH]	25,22	2,20	1	1	1	1
Q570B4	3-ketoacyl-CoA synthase 10 OS=Arabidopsis thaliana OX=3702 GN=FDH PE=1 SV=2 - [KCS10_ARATH]	24,97	1,45	1	1	1	1
Q9FX21	Dolichyl-diphosphooligosaccharide--protein glycosyltransferase subunit STT3B OS=Arabidopsis thaliana OX=3702 GN=STT3B PE=2 SV=1 - [STT3B_ARATH]	24,61	0,95	1	1	1	1
Q9LY99	Prohibitin-5, mitochondrial OS=Arabidopsis thaliana OX=3702 GN=PHB5 PE=1 SV=1 - [PHB5_ARATH]	24,28	2,81	1	1	1	1
Q8LC83	40S ribosomal protein S24-2 OS=Arabidopsis thaliana OX=3702 GN=RPS24B PE=2 SV=2 - [RS242_ARATH]	24,15	8,27	2	1	1	1
Q8VZH2	Aminopeptidase M1 OS=Arabidopsis thaliana OX=3702 GN=APM1 PE=1 SV=1 - [APM1_ARATH]	24,12	0,91	1	1	1	1
Q8LDP4	Peptidyl-prolyl cis-trans isomerase CYP19-4 OS=Arabidopsis thaliana OX=3702 GN=CYP19-4 PE=1 SV=2 - [CP19D_ARATH]	24,03	4,48	3	1	1	1
Q9FYQ8	Transmembrane 9 superfamily member 11 OS=Arabidopsis thaliana OX=3702 GN=TMN11 PE=2 SV=1 - [TMN11_ARATH]	23,93	1,37	1	1	1	1
Q8RX86	Alpha-galactosidase 2 OS=Arabidopsis thaliana OX=3702 GN=AGAL2 PE=1 SV=1 - [AGAL2_ARATH]	23,85	2,78	1	1	1	1
P46032	Protein NRT1/ PTR FAMILY 8.3 OS=Arabidopsis thaliana OX=3702 GN=NPF8.3 PE=1 SV=1 - [PTR2_ARATH]	23,68	2,22	2	1	1	1
O65390	Aspartic proteinase A1 OS=Arabidopsis thaliana OX=3702 GN=APA1 PE=1 SV=1 - [APA1_ARATH]	23,49	1,78	1	1	1	1
Q9LK73	UDP-glycosyltransferase 88A1 OS=Arabidopsis thaliana OX=3702 GN=UGT88A1 PE=2 SV=1 - [U88A1_ARATH]	23,34	1,30	2	1	1	1
Q8RWW1	Transmembrane 9 superfamily member 10 OS=Arabidopsis thaliana OX=3702 GN=TMN10 PE=2 SV=1 - [TMN10_ARATH]	22,93	1,88	1	1	1	1

Q680A5	Ribose-phosphate pyrophosphokinase 4 OS=Arabidopsis thaliana OX=3702 GN=PRS4 PE=1 SV=2 - [KPRS4_ARATH]	22,81	5,34	1	1	1	1
P54150	Peptide methionine sulfoxide reductase A4, chloroplastic OS=Arabidopsis thaliana OX=3702 GN=MSR4 PE=1 SV=2 - [MSRA4_ARATH]	22,57	3,88	1	1	1	1
Q9SE83	Dynamin-2A OS=Arabidopsis thaliana OX=3702 GN=DRP2A PE=1 SV=2 - [DRP2A_ARATH]	22,05	1,09	2	1	1	1
O04630	Threonine--tRNA ligase, mitochondrial 1 OS=Arabidopsis thaliana OX=3702 GN=THRRS PE=1 SV=3 - [SYTM1_ARATH]	22,05	1,27	1	1	1	1
P51424	60S ribosomal protein L39-1 OS=Arabidopsis thaliana OX=3702 GN=RPL39A PE=3 SV=2 - [RL391_ARATH]	21,83	19,61	1	1	1	1
P49637	60S ribosomal protein L27a-3 OS=Arabidopsis thaliana OX=3702 GN=RPL27AC PE=2 SV=2 - [R27A3_ARATH]	20,48	5,48	2	1	1	1
O64556	Putative leucine-rich repeat receptor-like serine/threonine-protein kinase At2g19230 OS=Arabidopsis thaliana OX=3702 GN=At2g19230 PE=1 SV=3 - [Y2923_ARATH]	20,47	0,80	1	1	1	1
P49201	40S ribosomal protein S23-2 OS=Arabidopsis thaliana OX=3702 GN=RPS23B PE=2 SV=2 - [RS232_ARATH]	16,75	7,75	2	1	1	1
